# Sensing and Reprogramming Surface Receptor Activation With Synthetic Transcriptional Circuits

**DOI:** 10.1002/advs.202522557

**Published:** 2026-02-23

**Authors:** Fei Liu, Mei Yuan, Li‐Juan Tang, Fenglin Wang, Xia Chu, Jian‐Hui Jiang

**Affiliations:** ^1^ State Key Laboratory of Chemo and Biosensing College of Chemistry and Chemical Engineering Hunan University Changsha P. R. China

**Keywords:** biosensors, cell‐specific therapy, RTK activity, surface receptor activation, synthetic biology

## Abstract

Cells rely on surface receptors to sense and initiate signalling cascades essential for numerous cellular processes, but engineering of synthetic genetic circuits to sense and rewire receptor activities for user‐defined cellular functions remains a challenge. Here we report a synthetic receptor‐signalling induced transcription (RESIT) circuit that enables sensing and reprogramming membrane‐localized receptor activation to pre‐defined transcriptional programs. The RESIT system is designed based on receptor activation mediated split protease complementation and release of membrane‐tethered synthetic transcriptional modules. We show that RESIT design is generally applicable to different transcriptional factors and various split viral proteases. This system is further engineered to probe Ca^2+^ entry accompanied with PIEZO1 induction and T cell activation, to detect oncogenic receptor tyrosine kinase (RTK) activities and to assess Ras activation proximal to plasma membranes. The versatility of RESIT system is repurposed to actuate diverse therapeutic functions including apoptosis induction, target protein degradation and T cell activation in cells with high RTK activities. The modularity and versatility of RESIT highlight its promise for interrogating juxtamembrane biochemical signaling and rewiring receptor activation to therapeutic functions.

## Graphical abstract

A synthetic receptor‐signalling induced transcription (RESIT) circuit is designed based on receptor activation mediated split protease complementation and release of membrane‐tethered synthetic transcriptional modules. The RESIT system enables probing Ca^2+^ entry, receptor tyrosine kinase (RTK) activities and Ras activation, and reprogramming RTK activities to diverse therapeutic programs.

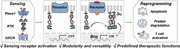

## 1 Introduction

Cells rely on surface receptors to sense extracellular cues and initiate signaling hubs proximal to the cell membranes to relay biochemical cascades that are essential for cell proliferation, differentiation and survival [1, 2]. The transduction of signaling networks can be achieved via initiating juxtamembrane messenger molecules or direct activation of receptor‐tethered enzymes [3]. For instance, Ca^2+^ entry is spatiotemporally induced upon activation of membrane‐localized receptors such as piezo‐type mechanosensitive ion channel component 1 (PIEZO1) and G‐protein coupled receptor (GPCRs) which plays pivotal roles in cellular physiology [4, 5]. Receptor tyrosine kinases (RTKs), a class of essential cellular signaling pathways in development and homeostasis, can be activated by extracellular ligand induced dimerization, creating membrane‐proximal phosphorylated docking sites for downstream signaling propagation [6, 7]. RAS, a small GTPase crucial in regulating cell migration, survival, growth and differentiation, can be activated by various surface receptors and relay information flow via binding partners [8]. Transduction of such receptor activation events through juxtamembrane signaling molecules are delicately regulated with high spatiotemporal dynamics to maintain cellular physiology. Abnormal receptor activities are implicated in various diseases including cancers and neurodegenerative diseases, making them promising therapeutic targets for drug development [9, 10]. Hence, the ability to sense receptor activation events and reprogram signaling pathways is of great significance in chemical biology and biomedicine.

Fluorescent protein (FP) based biosensors, which sense the interactions of signaling molecules through conformation changes or protein complementation with modulated fluorescence responses, have become the most common technique [11, 12, 13, 14, 15, 16]. Despite of the great potential, these FP‐based biosensors still exhibit limited signal‐to‐background (S/B) ratios [11]. Another technique is to visualize the activated molecules through multivalent protein−protein interaction mediated phase separation, which confers improved S/B ratios [17, 18]. Alternatively, synthetic transcriptional circuits, which engineer transcription factors (TFs) specific to the activated downstream signaling with the corresponding transgene reporters [19, 20, 21, 22, 23, 24] represent a distinct paradigm for receptor activation detection. They can not only transduce receptor activation into fluorescence responses through inducible expression of FP reporters, but also enable rewiring the receptor activities for therapeutic programs [20, 22]. Most of the current transcriptional circuits are developed to sense cytosolic signaling molecules, which may be compromised by lack of membrane‐localized activity information, limited sensitivity due to diffusion of the signaling molecules, or inferior selectivity ascribed to crosstalk with other signaling pathways [21]. To understand membrane‐proximal biochemical signals governing cellular receptor functions, it would be powerful to design synthetic genetic circuits that could sense juxtamembrane receptor activities. A prominent example is a compact synthetic genetic pathway, rewiring of aberrant signaling to effector release (RASER) [25], which could detect ErbB activation and co‐opt to trigger a therapeutic program via release of membrane‐tethered transcriptional or cell‐killing effectors. Despite the advances, general strategies to develop synthetic genetic circuits that can sense and rewire membrane‐proximal receptor activities for functional biochemical programs remains largely unexplored.

We thus considered a new generalized strategy for sensing receptor‐activated membrane‐proximal biochemical signals, including not only the direct receptor activation event but also its downstream signaling molecules. We hypothesized that membrane‐tethered split TF domains could be engineered to probe the membrane‐proximal biochemical signals, re‐assembled and released to initiate a user‐specified transcriptional program. Based on this hypothesis, we developed a synthetic *re*ceptor‐*s*ignalling *i*nduced *t*ranscription (RESIT) circuit, which enabled sensing membrane‐localized receptor activation or signaling molecules and reprogramming the receptor signals to transcription of a pre‐defined reporter or effector gene. The RESIT system was shown to be applicable to DNA binding domains (DBDs) from different TFs and various split viral proteases. The modularity and generality of the RESIT system was demonstrated for detecting different surface receptor activation events, including Ca^2+^ entry upon PIEZO1 induction and T cell activation, oncogenic RTK activities and Ras activation. The RESIT system was further exploited to reprogram high RTK activities to diverse customized therapeutic functions, including apoptosis induction, target protein degradation and T cell activation. The RESIT design exhibited a highly modular architecture and improved S/B ratios over their cytosol‐localized counterparts, highlighting its versatility and sensitivity for sensing membrane‐proximal signals. It could expand our arsenal for interrogating membrane‐proximal biochemical signals and rewiring dysregulated receptor activation to powerful therapeutic programs.

## 2 Results

### 2.1 Design of RESIT for Sensing Receptor Activation Through Juxtamembrane Interaction

**FIGURE 1:**
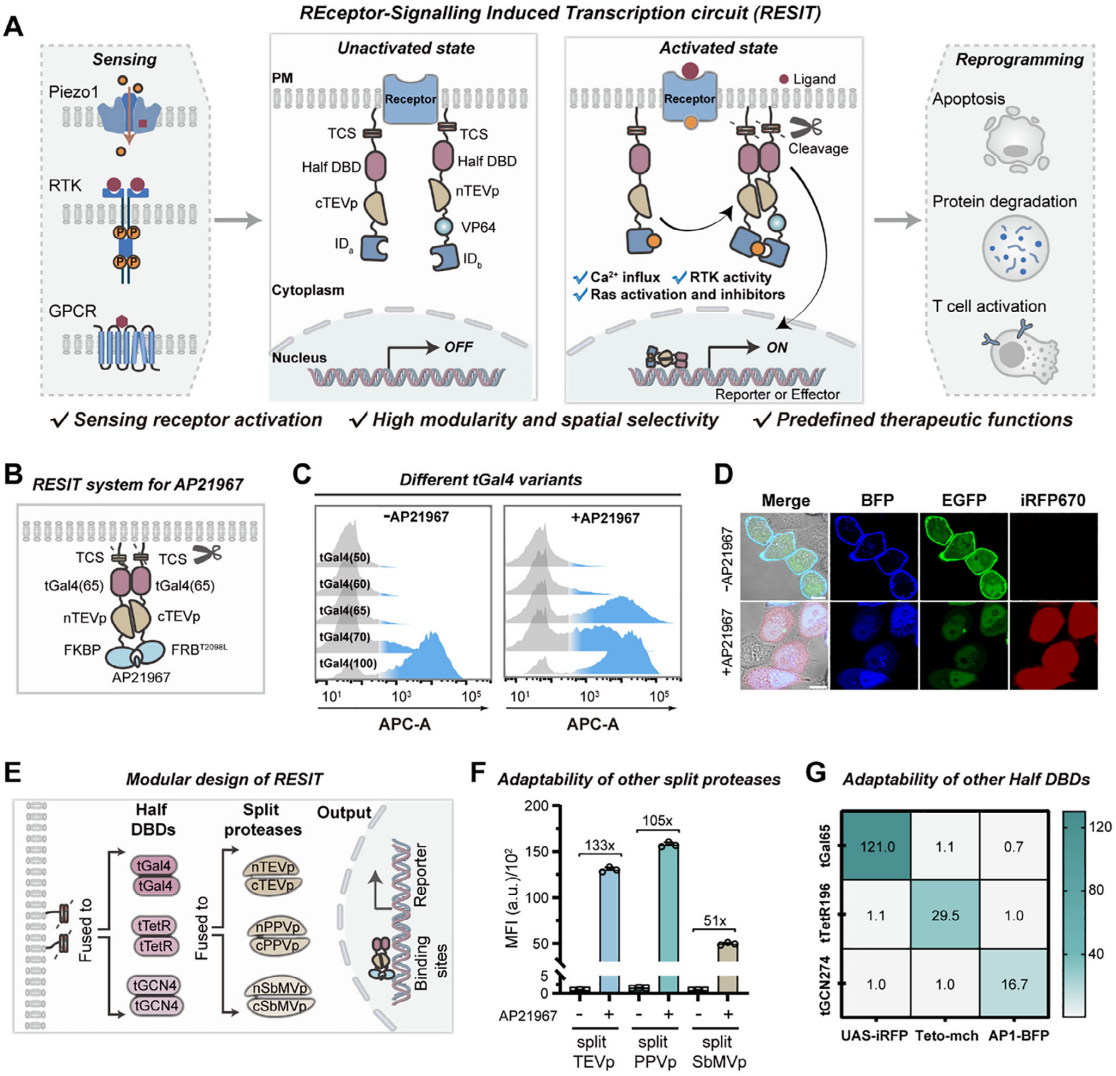
Engineer and characterization of RESIT system for sensing receptor activation. (A) Schematic of RESIT system for detecting RTK activities, Ras activation and Ca^2+^ influx accompanied with PIEZO1 induction and T cell activation. Upon receptor activation, ID_a_ and ID_b_ interaction induced complementation of split TEVp results in re‐assembly and release of membrane‐tethered transcriptional regulators to induce expression of various protein reporters or effectors. (B) Schematic of RESIT system responsive to AP21967. AP21967‐mediated interaction between FKBP and FRB^T2098L^ results in re‐assembly and release of tGal4(65) for transcription activation. (C) Flow cytometry profiles for HeLa cells transfected with RESIT with different tGal4 variants under AP21967 (1 µm) induction. (D) Representative confocal images for HeLa cells expressing RESIT system with or without AP21967 induction. iRFP670 fluorescence indicated the reporter. BFP fluorescence indicated the expression of tGal4(65)‐cTEVp‐FRB^T2098L^ module. EGFP fluorescence indicated the expression of tGal4(65)‐nTEVp‐FKBP module. Scale bar, 10 µm. (E) Modular design of the RESIT system using different split viral proteases or DBD domains from different TFs. (F) MFI of iRFP670 for HeLa cells expressing the RESIT system using split PPVp or SbMVp as the protease with or without AP21967 (1 µm) induction determined from flow cytometry profiles. (G) Heatmap showing specific activation of cognate reporters of tGal65, tTetR(196) and tGCN4(274) upon AP21967 induction. (C,D,F,G) Data were represented as mean ± s.d. of three independent measurements.

Signal transduction from activated cellular receptors is initiated through temporally juxtamembrane organization of protein−protein interactions. Considering the essential role of protein interactions in receptor signaling, we hypothesized that proximity interaction induced protease complementation was capable of probing various signaling molecules involved in receptor activation. Motivated by this hypothesis, we designed a RESIT system for sensing receptor activation through proximity induced complementation of split tobacco etch virus protease (TEVp) [19], allowing proteolytic release from membrane‐tethered transcriptional regulators to induce expression of protein reporters or effectors. This system comprised two membrane‐localized synthetic transcriptional modules, each having a N‐terminal membrane tether, a TEVp cleavage site (TCS), a half truncated inactive DBD, a split TEVp fragment and an interacting domain (ID_a_ and ID_b_), with an activator domain VP64 fused to one module (Figure 1A). Upon receptor activation, the two domains interact with each other, inducing irreversible complementation of two split TEVp fragments [23], which in turn mediated stable homo‐dimerization of the half DBDs, and release of the synthetic transcriptional modules for transcription activation.

It is noteworthy that the membrane‐tethered transcriptional circuit is highly modular, swapping of the interacting domains in the synthetic transcriptional modules enables detection of different biochemical signaling, adaptable for sensing activation of a variety of cellular receptors. Moreover, the membrane‐tethered design allows spatially selective detection of juxtamembrane signals, enabling more sensitive and specific query of the transient receptor activation states. Besides, the membrane‐tethered design confines the synthetic transcriptional modules within a 2D surface instead of in a 3D volume, increasing the effective local concentration of reactants with remarkably facilitated reaction kinetics and thus improving the sensing sensitivity [26].

To demonstrate the potential of this RESIT system, we chose to detect Ca^2+^ entry upon PIEZO1 and T cell activation, oncogenic RTK activity and Ras activation upon EGFR activation. Note that RESIT had a highly modular architecture in which only the interacting domains were swapped for sensing different signaling molecules. Hence, we preliminarily focused on engineering the invariable regions in RESIT using a model system, interaction between FK506‐binding protein (FKBP) and FKBP‐rapamycin binding domain mutant (FRB^T2098L^) [27] under a rapamycin analog AP21967 induction (Figure 1B). These two invariable regions consisted of four domains, a N‐terminal membrane tether, a TCS, a half truncated DBD, and a split TEVp fragment. To facilitate independent membrane tethering, we used a pleckstrin homology (PH) domain from phospholipase C delta 1, which was known to bind phosphatidylinositol 4,5‐bisphosphate in phospholipid membranes [28], and a N‐myristoylation (N‐myr) substrate peptide [28] for membrane targeting of two transcriptional modules, respectively. The N‐terminal truncated Gal4 (tGal4) was chosen as the half DBD. We generated different tGal4 variants by progressively shortening the α‐helix at C‐terminus, and tested AP21967‐induced transcriptional activation of iRFP670 reporter under a 5 × UAS−miniCMV promoter. It was found that the optimal variant tGal4(65) exhibited very low fluorescence background, and delivered substantial fluorescence enhancement by 103‐fold upon AP21967 induction (Figures 1C,D; S1). A control experiment with FKBP or FRB^T2098L^ deleted in one transcriptional module confirmed the specificity of transcriptional activation to FKBP−FRB^T2098L^ interaction (Figure S2). Further optimization of the plasmid amounts revealed that the highest signal‐to‐background was obtained when the ratios of the two membrane‐tethered modules and reporter were 1:3:0.2 (Figure S3). In addition, the RESIT design using a wild‐type Gal4 obtained higher iRFP670 fluorescence background without AP21967 induction (Figure S4). This result suggested that the utility of wild‐type Gal4 would lead to substantial leakage of the transcriptional modules, whereas the halved DBD design conferred the benefit in minimizing the background. Besides, the cytosolic‐localized RESIT without membrane tethering afforded significantly lower signal‐to‐background ratio (Figure S5).

We investigated the effects of protein fusion order and combinations for the membrane‐tethered transcriptional modules on signal production. We found that the combination of PH−tGal4(65)−cTEVp−FRB^T2098L^ and N‐myr− tGal4(65)−FKBP−nTEVp afforded the highest S/B ratio of 132 (Figure S6). Further investigation revealed dynamic correlation of the iRFP670 fluorescence signals to AP21967 concentrations in the range from 0.001 to 0.01 µm (Figure S7). Time‐dependent measurements showed detectable fluorescence signals at 4 h, which gradually increased through 24 to 60 h after AP21967 induction (Figure S8). Moreover, the RESIT system showed consistent results in different cell lines including MCF‐7 (human breast cancer cell line), HEK293T (human embryonic kidney cell) and NIH 3T3 (mouse embryonic fibroblast cell) (Figure S9). Besides, we used another secretary Gaussia luciferase (Gluc) as the reporter for the transcriptional circuit, and a S/B ratio of 115 was obtained (Figure S10). We also investigated the possibility of designing RESIT systems responsive to other inducers by replacing the interacting domains with NS3a and DNCR2 or ABI and PYL1 [29], which can bind their cognate inducers danoprevir or abscisic acid. We found that RESIT was specifically activated by the cognate inducers with dose dependency (Figures S11 and S12), verifying the general applicability of the RESIT system to different inducers.

We then interrogated the feasibility of adapting other split viral proteases in RESIT, including plum pox virus protease (PPVp) and soybean mosaic virus protease (SbMVp) [30] (Figure 1E). We found that the RESIT system based on split PPVp and SbMVp delivered S/B ratios of 105‐ and 51‐fold, respectively (Figures 1F; S13 and S14). We further investigated the general adaptability of RESIT to other DBD domains, including truncated TetR and GCN4 (Figure 1E). It was found that tTetR(196) and tGCN4(274) displayed the best S/B ratios of 31‐ and 16‐fold, respectively, with concentration‐dependent transcriptional activation upon AP21967 induction (Figures S15 and S16). Besides, tGal4(65), tTetR(196) and tGCN4(274) specifically activated their cognate reporters in the prescence of AP21967, showing their high orthoganality (Figure 1G). Together, these results validated the successful engineering of the RESIT system, and the optimal design was achieved using the truncated variant of Gal4 and the split TEVp.

### 2.2 RESIT System for Sensing Time‐Integrated Ca^2+^ Entry

**FIGURE 2:**
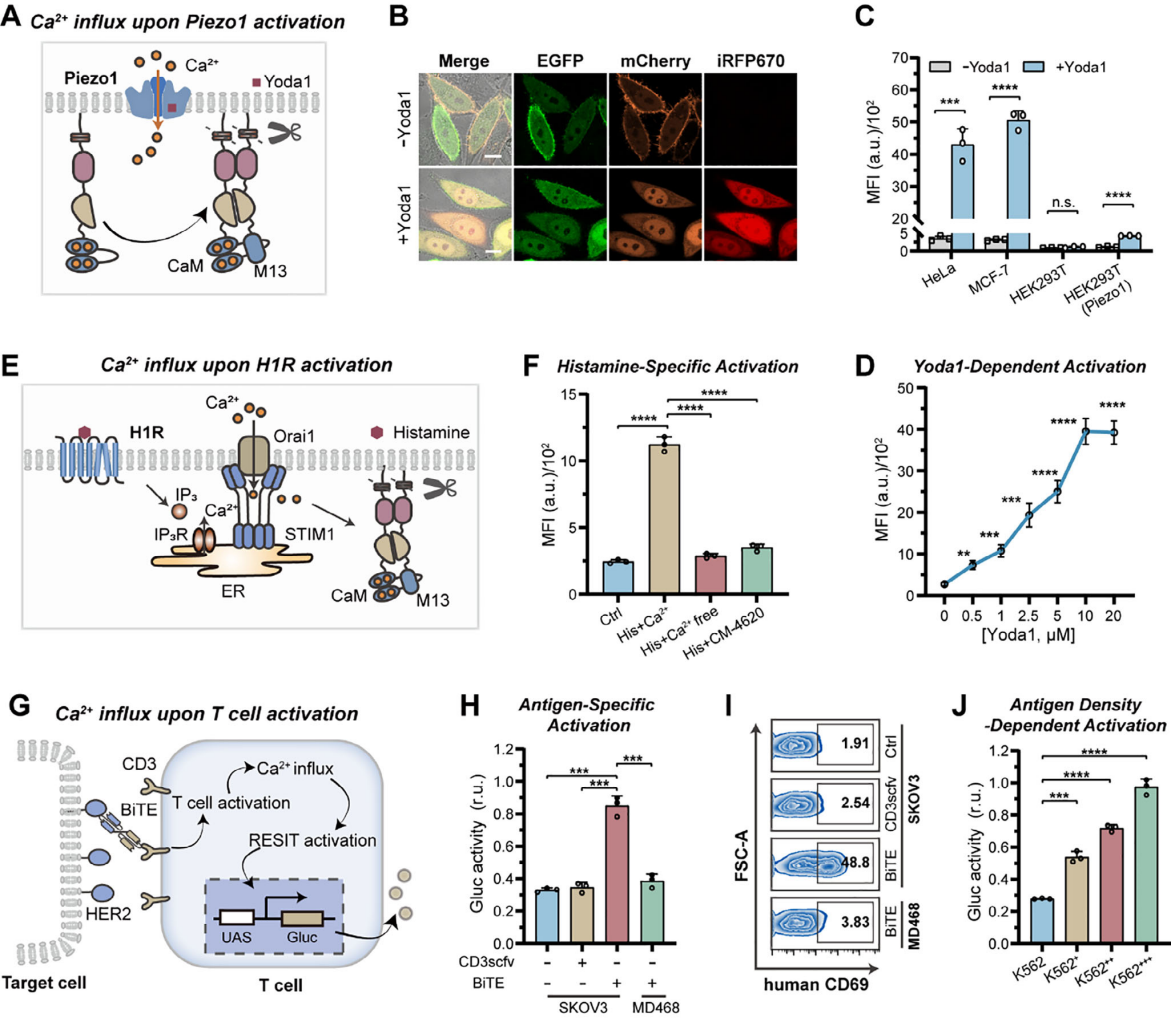
Design of RESIT system responsive to Ca^2+^ influx across plasma membrane. (A) Schematic for RESIT system responsive to Ca^2+^ influx upon Piezo1 activation. Piezo1 activation mediated Ca^2+^ influx induces CaM−M13 binding which complements split TEVp for transcriptional circuit actuation. (B) Representative confocal images for HeLa cells expressing RESIT system responsive to Ca^2+^ influx with or without Yoda1 (10 µm) induction. iRFP670 fluorescence indicated the reporter. EGFP fluorescence indicated the expression of tGal4(65)‐nTEVp‐CaM module. mCherry fluorescence indicated the expression of tGal4(65)‐cTEVp‐M13 module. Scale bar, 10 µm. (C) MFI of iRFP670 for different cell lines expressing the RESIT system with or without Yoda1 (10 µm) induction as determined from flow cytometry data. Statistical analysis was performed using a two‐tailed t‐test (Left to right: ^***^ *p* = 0.0002, ^****^ *p* < 0.0001, p = 0.1440, ^****^ *p* < 0.0001). (D) Dose‐dependent responses of the RESIT system to Yoda1 as determined by MFI of iRFP670 from flow cytometry data. The significance of differences in cells treated with different concentrations of Yoda1 vs. the control was determined using single‐factor ANOVA (Left to right: ^**^ *p* = 0.0022, ^***^ *p* = 0.0009, ^***^ *p* = 0.0006, ^****^ *p* < 0.0001). (E) Schematic of RESIT system for sensing Ca^2+^ entry triggered by histamine mediated activation of H1R. Histamine binds to H1R, which triggers Ca^2+^ release from ER and evokes Ca^2+^ entry cross the plasma membrane via the Orail channel. (F) MFI of iRFP670 for MCF‐7 cells co‐expressing H1R and the RESIT system treated with histamine (100 µm), histamine in Ca^2+^ free medium or histamine and the channel inhibitor CM4620 (1 µm). The significance of differences in cells with different treatments vs. those treated with histamine was determined using single‐factor ANOVA (^****^ *p* < 0.0001). (G) Schematic of RESIT system for sensing Ca^2+^ influx accompanied with T cell activation. (H) Gluc activity for Jurkat T cells transduced with RESIT system co‐cultured with MDA468 or SKOV3 cells in the presence of a BiTE or a CD3 scfv only. The levels of secreted Gluc in the supernatants were determined. Statistical analysis was performed using a two‐tailed t‐test (Left to right: ^***^ *p* = 0.0001, ^***^ *p* = 0.0002, ^***^ *p* = 0.0004). (I) Flow cytometry profiles for Jurkat T cells in (H) stained by APC‐labeled anti‐CD69 antibody. (J) Gluc activity for Jurkat T cells transduced with RESIT system co‐cultured with K562 cells with different HER2 densities in the presence of a BiTE. The levels of secreted Gluc in the supernatants were determined. Statistical analysis was performed using a two‐tailed *t*‐test (Left to right: ^***^ *p* = 0.0002, ^****^ *p* < 0.0001). (C, D, F, H‐J) Data were represented as mean ± s.d. of three independent measurements.

We then explored to design a RESIT system for sensing Ca^2+^ entry across the plasma membrane. Ca^2+^ influx can be triggered by a wide variety of biological processes including activation of PIEZO1 and GPCR, and Ca^2+^ release from endoplasmic reticulum (ER) [31]. Spatiotemporal regulation of Ca^2+^ entry is essential for mechanotransduction, differentiation and immunity [4]. Sensors for Ca^2+^ entry across plasma membranes could facilitate development of new therapies for cardiovascular disease, cancer and regenerative medicine. To probe Ca^2+^ signaling proximal to plasma membrane, we re‐engineered the RESIT system by replacing the interaction domains, respectively, with calmodulin (CaM) and a CaM binding peptide M13 [32]. Upon activation, Ca^2+^ entered cells through the cation channels, which mediated CaM−M13 binding accompanied with split TEVp complemented and transcriptional circuit activated (Figure 2A). As anticipated, RESIT displayed greatly enhanced iRFP fluorescence in cells treated with Ca^2+^ and ionomycin [33] a chelator that facilitated Ca^2+^ permeation (Figure S17). Further optimization of the plasmids amounts afforded the highest activation fold when the ratios for the two membrane‐tethered modules and reporter were 1:3:0.2 (Figure S18). Moreover, the iRFP dynamically increased as Ca^2+^ concentrations increased from 0.25 to 4 mM (Figure S17). This range covered the concentrations of Ca^2+^ entry under physiological induction [4], and was consistent with previously reported genetically‐encoded calcium indicators [34]. Besides, time‐dependent measurements showed that RESIT afforded evident fluorescence at 2 h after incubating with Ca^2+^ and ionomycin (Figure S19). In addition, RESIT displayed appreciable signal after incubating with Ca^2+^ and ionomycin for 30 s which increased as incubation time prolonged, suggesting the capability of RESTI for integrating a transient signal to transcriptional activation.

We further investigated the capability of this RESIT system in imaging Ca^2+^ entry upon PIEZO1 activation. As anticipated, RESIT displayed negligible iRFP fluorescence in HeLa cells (PIEZO1‐positive) without induction, but the iRFP response was remarkably increased after treatment with Yoda1, a small‐molecule PIEZO1 activator (Figures 2B,C; S20). By contrast, RESIT delivered negligible fluorescence responses in HEK293T cells (PIEZO1‐negative) under Yoda1 induction, but exhibited intense fluorescence after exogenous transfection of a PIEZO1‐expressing plasmid. This system also showed Yoda1‐induced fluorescence responses in MCF‐7 cells with positive PIEZO1 expression. An optimization of the system using different M13 variants afforded the highest activation fold of ∼15 (Figure S21), which was used for subsequent study. A control experiment with M13 deleted verified the specificity of RESIT to Ca^2+^‐mediated interaction between CaM and M13 (Figure S22). Moreover, the fluorescence activation exhibited dose‐dependency in HeLa cells under Yoda1 induction (Figure 2D). Besides, we showed that the membrane‐tethered RESIT afforded much higher fluorescence as compared to the membrane‐tether deleted counterpart, indicating the essential role of membrane‐tethering design in sensitivity enhancement (Figure S23). These results demonstrated the ability of the RESIT system for quantitatively imaging Ca^2+^ entry upon PIEZO1 activation.

We also explored the possibility of the RESIT system for imaging Ca^2+^ entry cross the plasma membrane evoked by Ca^2+^ release from ER. We found that the RESIT displayed intense fluorescence signal upon thapsigargin (TG) induction (Figure S24), which was ascribed to Ca^2+^ entry across the Ca^2+^ channel triggered by TG‐mediated Ca^2+^ releasing from ER [31]. A control experiment for cells cultured in Ca^2+^‐free medium or treated with a Ca^2+^ channel inhibitor testified the essential role of Ca^2+^ entry in TG‐evoked response (Figure S24). Interestingly, the membrane‐tether deleted counterpart of RESIT delivered much weaker signal for cells induced with TG. This result clearly demonstrated the improved sensitivity of the RESIT system in probing juxtamembrane Ca^2+^ entry as compared to its cytosolically localized counterpart.

We then employed the RESIT to detect Ca^2+^ entry triggered by histamine induced activation of a mechanosensitive GPCR, histamine H1 receptor (H1R) (Figure 2E), which mediated Ca^2+^ release from ER followed by Ca^2+^ entry across the Ca^2+^ channel [31]. There was negligible fluorescence response for RESIT in MCF‐7 cells (H1R‐negative) upon histamine addition (Figure S25). By contrast, RESIT delivered intense fluorescence for MCF‐7 cells with exogenous transfection of a H1R‐expressing plasmid upon histamine induction. Control experiments for cells cultured in Ca^2+^‐free medium or treated with a Ca^2+^ channel inhibitor also validated the specificity of histamine‐induced Ca^2+^ entry (Figure 2F). These results demonstrated the capability of the RESIT system for imaging Ca^2+^ entry accompanied with GPCR activation.

Next, we asked whether the RESIT system could image Ca^2+^ entry associated with T cell activation. It was documented that Ca^2+^ influx was accompanied with T cell activation and governs T cell functions and immune responses [4]. To detect Ca^2+^ entry in T cells, we packaged the RESIT system in two lentiviral vectors with the two membrane‐tethered modules in a lentiviral vector under promoters of CMV and EF1α, and the Gluc reporter module in another lentiviral vector under a miniCMV promoter (Figure 2G). Jurkat T cells were transduced with these two vectors, and co‐cultured with target cells in the presence of a bispecific T cell engager (BiTE) consisting of an HER2 nanobody and a CD3‐specific single‐chain variable fragments (scfv). We observed very low Gluc activity for transduced T cells co‐cultured with MD468 cells with negative expression of HER2 in the presence of BiTE (Figures 2H; S26). By contrast, the Gluc activity was remarkably increased for transduced T cells co‐cultured with SKOV3 cells with high expression of HER2, suggesting high Ca^2+^ entry. Control experiments for transduced T cells co‐cultured with SKOV3 cells in the presence of CD3 scfv only verified the specific activation of Ca^2+^ entry by BiTE. The specific activation of T cells upon co‐colturing with SKOV3 cells in the presence of the BiTE was confirmed with upregulation of CD69, a typical activation marker (Figure 2I). Moreover, transduced T cells delivered dynamically increased Gluc activities upon co‐culturing with K562 cells with increasing expressions of HER2 obtained via lentivirus transduction and fluorescence‐activated cell sorting (Figures 2J; S27). Besides, HER2 expression dependent T cell activiation was verified by CD69 levels. Together, these results confirmed the ability of RESIT to specifically sense Ca^2+^ entry upon T cell activation.

### 2.3 RESIT System for Sensing RTK Activity

**FIGURE 3:**
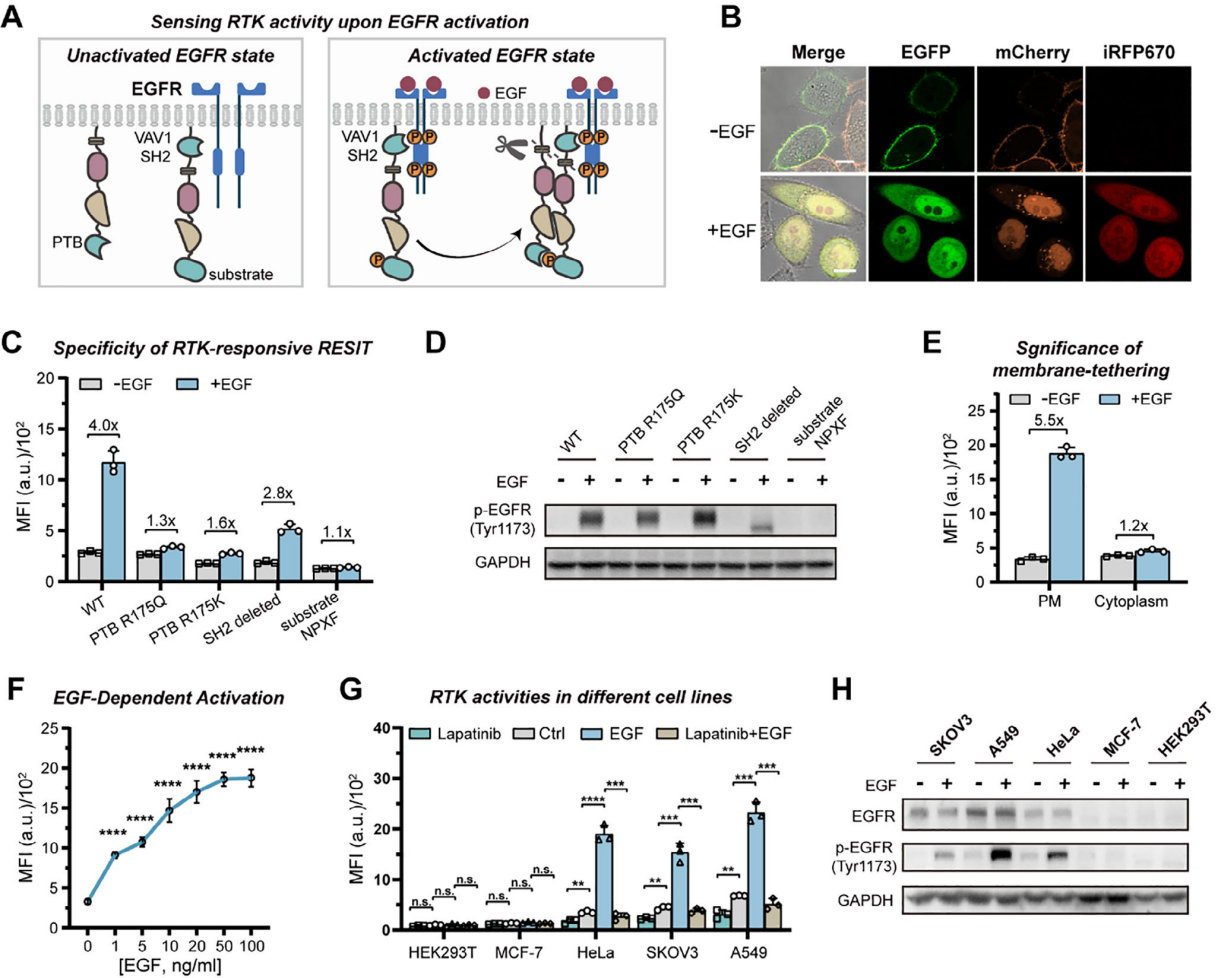
Design of RESIT system responsive to RTK activity upon EGFR activation. (A) Schematic of RESIT for sensing RTK activity upon EGFR activation. In the unactivated RTK state (left), the synthetic transcriptional modules remain unassembled and are tethered to the plasma membrane. In the activated RTK state (right), RTK phosphorylates the proximate substrate (ID_b_) and mediates interaction with the PTB domain (ID_a_), inducing split TEVp complementation and transcriptional circuit actuation. (B) Representative confocal images for HeLa cells expressing the RTK‐responsive RESIT treated with or without EGF (100 ng/mL). iRFP670 fluorescence indicated the reporters. EGFP fluorescence indicated the expression of VAV1−tGal4(65)−nTEVp−NPXY module. mCherry indicated the expression of tGal4(65)−cTEVp−PTB module. Scale bar, 10 µm. (C) Specificity of RTK‐responsive RESIT design for sensing RTK activity. MFI of iRFP670 for HeLa cells expressing the RTK‐responsive RESIT system with wide type (WT) PTB, PTB variant R175Q, PTB variant R175K, VAV1 SH2 deletion and a mutant substrate motif NPXF (Y1173F) in the sensing modules with or without EGF (100 ng/mL) induction. (D) WB analysis of phosphorylation levels of the designed substrate NPXY for cells in (C). (E) MFI of iRFP670 as determined from flow cytometric data for comparison of membrane‐tethered RESIT (PM) and cytosol‐localized RESIT (Cytoplasm) in sensing RTK activity. (F) MFI of iRFP670 for cells expressing the RTK‐responsive RESIT treated with different concentrations of EGF. The significance of differences in cells treated with different concentrations of EGF vs. the control was determined determined using single‐factor ANOVA (^****^ *p* < 0.0001). (G) MFI of iRFP670 for cell lines (HEK293T, MCF‐7, HeLa, SKOV3 and A549 cells) with different RTK activities expressing RTK‐responsive RESIT (Ctrl), treated with lapatinib (0.5 µm), EGF (50 ng/mL) or EGF and lapatinib as determined from flow cytometry data. Statistical analysis was performed using a two‐tailed t‐test (Left to right: *p* = 0.2136, *p* = 0.8655, *p* = 0.5929, *p* = 0.1729, *p* = 0.1414, *p* = 0.0876, ^**^ *p*  =  0.0038, ^****^ *p* < 0.0001, ^***^ *p*  =  0.0001, ^**^ *p*  =  0.0014, ^***^ *p*  =  0.0005, ^***^ *p*  =  0.0004, ^**^ *p*  =  0.0017, ^***^ *p*  =  0.0002, ^***^ *p*  =  0.0002). (H) WB analysis of EGFR and phosphorylated EGFR (pEGFR) levels in HEK293T, MCF‐7, HeLa, SKOV3 and A549 cells induced with or without EGF (50 ng/mL). (B,C,E–G) Data were represented as mean ± s.d. of three independent measurements.

We then engineered the system for sensing RTK activity using EGFR as a model, which was aberrantly elevated and implicated in various human cancers [6]. Detection of RTK activity could facilitate elucidating the oncogenic and drug resistance mechanisms behind EGFR mutations, affording new clues for drug discovery. To probe RTK activity, we re‐engineered two transcriptional modules in RESIT as followed (Figure 3A): substituting a peptide substrate motif (NPXY) for ID_b_ and incorporating the Src homology 2 (SH2) domain from VAV1 [25] between the N‐terminal membrane tether and TCS in one transcriptional module, and substituting the phosphotyrosine binding domain (PTB) from SHC1 [25] for ID_a_ in the other transcriptional module. The design of VAV1 SH2 domain incorporation was used for recruiting the corresponding transcriptional module in close proximity to EGFR [25]. Once EGFR activated, RTK could phosphorylate the proximate NPXY motif and mediate its interaction with the PTB domain, thereby inducing split TEVp complementation and actuating the transcriptional circuit.

We then tested the RTK‐responsive RESIT system using HeLa cell line, which had moderate EGFR expression with negligible basal EGFR activation. As anticipated, RESIT showed very low iRFP fluorescence without epidermal growth factor (EGF) induction, but exhibited intense iRFP upon EGF stimulation (Figures 3B; S28). Control experiments using a mutant substrate motif NPXF or mutant PTBs incapable of binding to the phosphorylated substrate validated that the RESIT system was selective to both substrate phosphorylation and its binding to PTB, indicating the high specificity of RESIT to RTK activity and its downstream signaling (Figure 3C). Notably, the VAV1 SH2 domain was essential in the RESIT design. A control design without the SH2 domain delivered remarkably decreased fluorescence response, suggesting that recruitment of the substrate motif to RTK was crucial for its efficient phosphorylation. Western blotting using the anti‐phosphotyrosine antibody confirmed the specific phosphorylation of the substrate (NPXY) upon EGF induction with dramatically decreased phosphorylation for the substrate without the SH2 domain (Figure 3D). Moreover, a tandem substrate design using three substrate motifs afforded enhanced fluorescence responses (Figure S29), probably ascribed to its increased substrate phosphorylation. Besides, a close interrogation of the domain fusion order in the membrane‐tethered modules revealed the optimal combinations of PH−VAV1−tGal4(65)−nTEVp−3 × NPXY and N‐myr− tGal4(65)−cTEVp−PTB, which afforded the best S/B ratio of ∼6 (Figure S30). Thus, we chose this RESIT design for further study. Notably, a control design with the membrane‐tether deleted in RESIT delivered very low fluorescence enhancement under EGF induction (Figure 3E). This remarkably decreased activation was probably due to the micromolar affinity of VAV1 to EGFR [35], resulting in limited translocation of the transcriptional modules to the plasma membrane for signal activation. A closer interrogation revealed negligible translocation of the cytosolic‐localized synthetic modules to plasma membrane after EGF induction (Figure S31). This result suggested the significance of membrane‐tethering design in the RESIT design for probing RTK activities with remarkable improvement of the S/B ratio. In addition, RESIT delivered negligible iRFP fluorescence upon induction of a non‐cognate receptor fibroblast growth factor receptor, suggesting that the signal was mainly ascribed to EGFR‐specific activation (Figure S32).

We found that this system exhibited dose‐dependent fluorescence enhancement under EGF induction, indicating the ability of the RESIT system for quantitatively detecting EGFR activation (Figure 3F). We employed the system to detect endogenous RTK activities in different cell lines including HEK293T, MCF‐7, HeLa, SKOV3 (human ovarian cancer) and A549 (human lung cancer) cells with different expression levels of EGFR (Figure S33). We found that HEK293T and MCF‐7 cells delivered negligible iRFP in the absence or presence of EGF (Figure 3G). By contrast, HeLa, SKOV3 and A549 cells delivered varying iRFP fluorescence signals in the absence of EGF, which greatly increased after EGF induction. A further control for cells pretreated with an EGFR/ErbB2 inhibitor lapatinib [25], or EGF and lapatinib delivered negligible iRFP fluorescence, indicating that the fluorescence signals were ascribed to EGFR‐specific activation. Besides, the iRFP fluorescence signals were consistent with RTK activities as determined using Western blotting (WB) (Figure 3H). Together, these results demonstrated the ability of RESIT for quantitatively detecting juxtamembrane RTK activities in different cells.

### 2.4 RESIT System for Sensing Ras Activity and Assessing Inhibitor Efficacy

**FIGURE 4:**
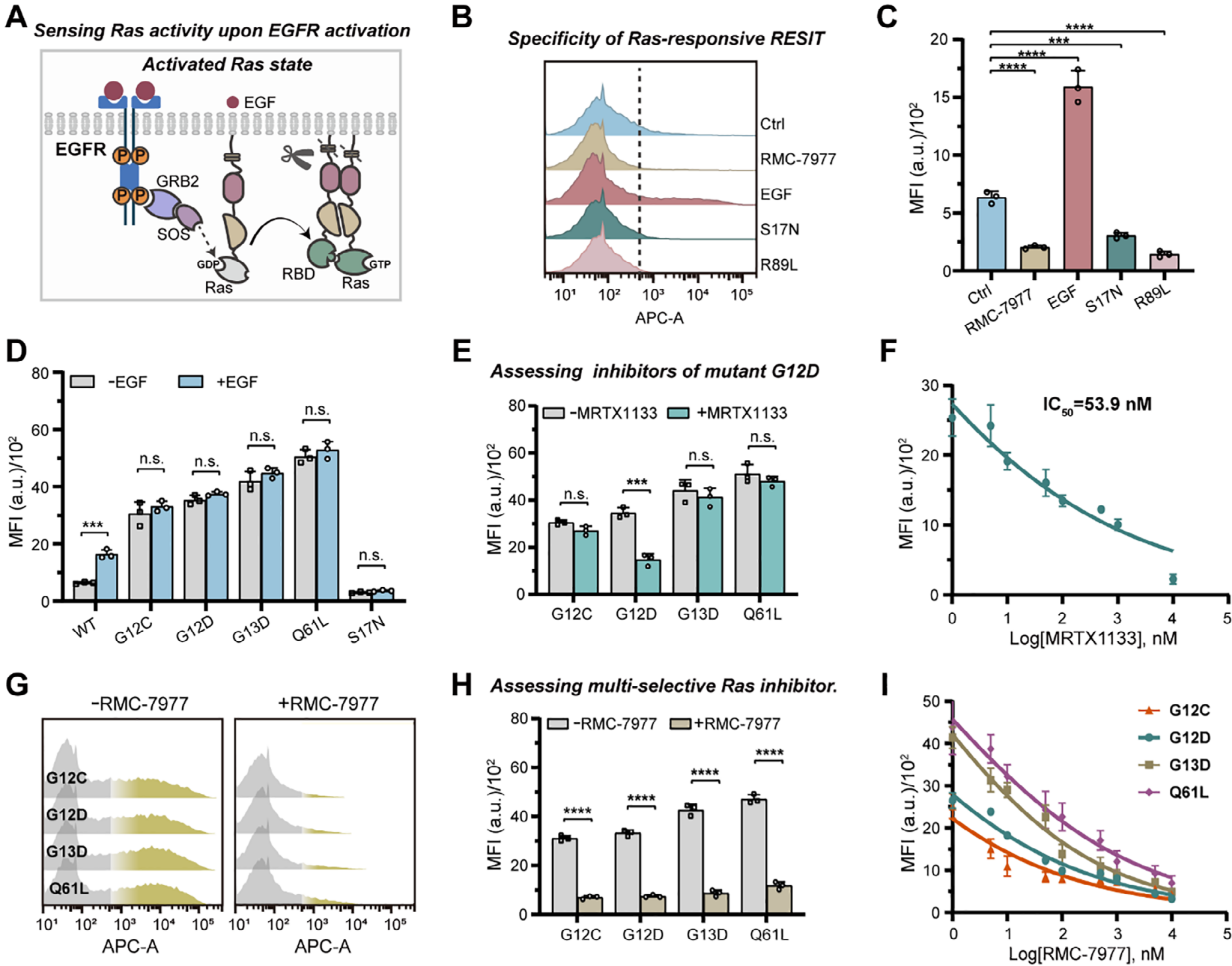
Engineer of RESIT system for sensing Ras activity and evaluating inhibitor efficacy. (A) Schematic of RESIT for sensing Ras activity. Upon RTK activation, the phosphorylated sites create docking sites for Grb2 which binds SOS1 and activates Ras. The activated Ras (ID_a_) interacts with Raf1 (ID_b_) to mediate split TEVp complementation for transcriptional circuit actuation. (B) Flow cytometry profile for cells expressing RAS‐responsive RESIT (Ctrl), treated with RMC7977 (1 µm) or EGF (50 ng/mL), and cells expressing RESIT with Ras mutant (S17N) or Raf mutant (R89L) in the sensing modules. (C) MFI of iRFP670 as determined from flow cytometric data in (B). The significance of differences in cells with different treatments vs. those expressing RESIT only was determined using single‐factor ANOVA (Left to right: ^****^ *p* < 0.0001, ^****^ *p* < 0.0001, ^***^ *p* = 0.0005, ^****^ *p* < 0.0001). (D) RESIT system in sensing the activities of Ras mutants (G12C, G12D, G13D, Q61L and S17N) with or without EGF (50 ng/mL) induction as determined by MFI of iRFP670 from flow cytometry data. Statistical analysis was performed using a two‐tailed t‐test (Left to right: ^***^ *p* = 0.0004, *p* = 0.3969, *p* = 0.1094, *p* = 0.2574, *p* = 0.3539, *p* = 0.0512). (E) RESIT system in assessing the effect of MRTX1133 (50 nm) on the activities of different Ras mutants (G12C, G12D, G13D and Q61L) in HeLa cells as determined by MFI of iRFP670 from flow cytometry data. Statistical analysis was performed using a two‐tailed t‐test (Left to right: p = 0.0508, ^***^ *p* = 0.0005, *p* = 0.4392, *p* = 0.2886). (F) Dose‐dependent effect of MRTX1133 on Ras mutant (G12D) in HeLa cells as determined by MFI of iRFP670 from flow cytometry data. (G) RESIT system in assessing the efficacy of RMC‐7977 (1 µm) in inhibiting the activities of different Ras mutants (G12C, G12D, G13D, and Q61L) as determined by flow cytometry. (H) MFI of iRFP670 for cells in (G). Statistical analysis was performed using a two‐tailed t‐test (^****^ *p* < 0.0001). (I) Dose‐dependent effect of RMC‐7977 on Ras mutants (G12C, G12D, G13D, and Q61L) in HeLa cells as determined by MFI of iRFP670 from flow cytometry data. (B–D,E–H,I) Data were represented as mean ± s.d. of three independent measurements.

We then extended the RESIT design for detecting Ras activity using a model system of EGFR activation. Upon EGFR activation, transphosphorylation of the intracellular C‐terminal tail creates docking site for growth factor receptor binding protein 2 (Grb2), which binds son of sevenless 1 (SOS1) followed by activation of Ras [8]. Apart from the downstream signaling of EGFR, Ras also acted as a critical regulator of many oncogenic cell growth and proliferation pathways [8]. Hence, detection of Ras activity was crucial for understanding Ras biology and developing Ras therapeutics. To probe Ras activity, we re‐engineered the RESIT system by replacing the interaction domains in the two transcriptional modules with Ras and the Ras binding domain Raf1, respectively (Figure 4A). It was noteworthy that C‐terminal of Ras was deleted in the corresponding transcriptional module to allow for its membrane‐tethering through the PH domain at N‐terminal [36]. Upon activation, the active RAS could interact with the Raf1 domain, mediating split TEVp complementation with concomitant actuation of the engineered transcriptional circuit.

We then investigated the RESIT system for sensing Ras activity upon EGFR activation. After transfection in HeLa cells, RESIT displayed appreciable iRFP670 fluorescence and the iRFP670 response exhibited a further 3‐fold increase upon EGF induction (Figure 4B). The iRFP fluorescence in the absence of EGF was probably ascribed to the basal Ras activity. A control experiment revealed that this basal Ras activity could be almost completely inhibited under treatment of RMC‐7997 [37], a multi‐selective inhibitor for Ras. A further control experiment showed that an inactive Ras mutant or a Raf mutant incapable of binding active Ras only delivered negligible iRFP670 responses, suggesting that RESIT was specific to interaction between active Ras and Raf1 (Figures 4C; S34). Additionally, we optimized the domain fusion order in the membrane‐tethered modules, and obtained an optimal combination PH−tGal4(65)−cTEVp−Ras and N‐myr−tGal4(65)−nTEVp−Raf1 for the transcriptional circuit (Figure S35) in subsequent study. More importantly, we also validated the essential role of membrane‐tethering design of RESIT in significant sensitivity enhancement. Indeed, the membrane‐tether deleted version, wherein the PH domain and N‐myr motif were deleted, exhibited much lower fluorescence enhancement under EGF induction (Figure S36).

Ras mutations were considered to account for various human cancers, and these mutations used to occur at hotspot codons G12, G13 or Q61 and entail gain‐of‐function missense alterations [37]. We thus interrogated the utility of the genetic circuit for sensing Ras activity in distinct Ras mutants to shed light on Ras biology and drug discovery. We engineered one of the transcriptional modules in RESIT using different Ras mutants including G12C, G12D, G13D, Q61L and S17N. After transfection of each of these Ras‐mutated genetic circuits in HeLa cells, we found strong iRFP signals for Ras mutants G12C, G12D, G13D and Q61L even without EGF induction, whereas Ras mutant S17N exhibited negligible fluorescence even after EGF induction (Figures 4D; S37). This result suggested that mutants (G12C, G12D G13D and Q61L) each exhibited constitutive activity, while mutant (S17N) was dominantly inactive, which were consistent with literature reports [38].

Next, we utilized the RESIT system for screening inhibitors of mutant G12D vs. mutants G12C, G13D and Q61L. A reported inhibitor MRTX1133 [39], which selectively bound GDP‐bound Ras G12D to prevent nucleotide exchange, was first investigated. We found that the RESIT circuit with G12D‐mutated Ras displayed remarkably decreased iRFP fluorescence under treatment with MRTX1133, but no appreciable inhibition was obtained for those systems with Ras G12C, G13D and Q61L (Figure 4E). Moreover, we observed dynamically decreased iRFP signals with increasing MRTX1133 concentrations, indicating the potential of RESIT for dose‐dependent evaluation of inhibitors for Ras G12D mutant in live cells (Figure 4F). We further evaluated RMC‐7977 [37], a multi‐selective Ras inhibitor. Upon treatment with RMC‐7977, the Ras‐mutated RESIT systems (G12C, G12D, G13D or Q61L) all displayed substantially inhibited fluorescence responses with dose dependent decrease (Figures 4G–I; S38). This result implied that MRTX1133 was an allele‐specific inhibitor for Ras G12D, while RMC‐7977 was a broad‐spectrum pan‐inhibitor. Together, these results demonstrated the ability of the RESIT system for sensing juxtamembrane Ras activity and screening potential Ras‐targeting therapies.

### 2.5 Reprogramming Oncogenic RTK Activity for Cell‐Specific Therapy Using RESIT System

**FIGURE 5:**
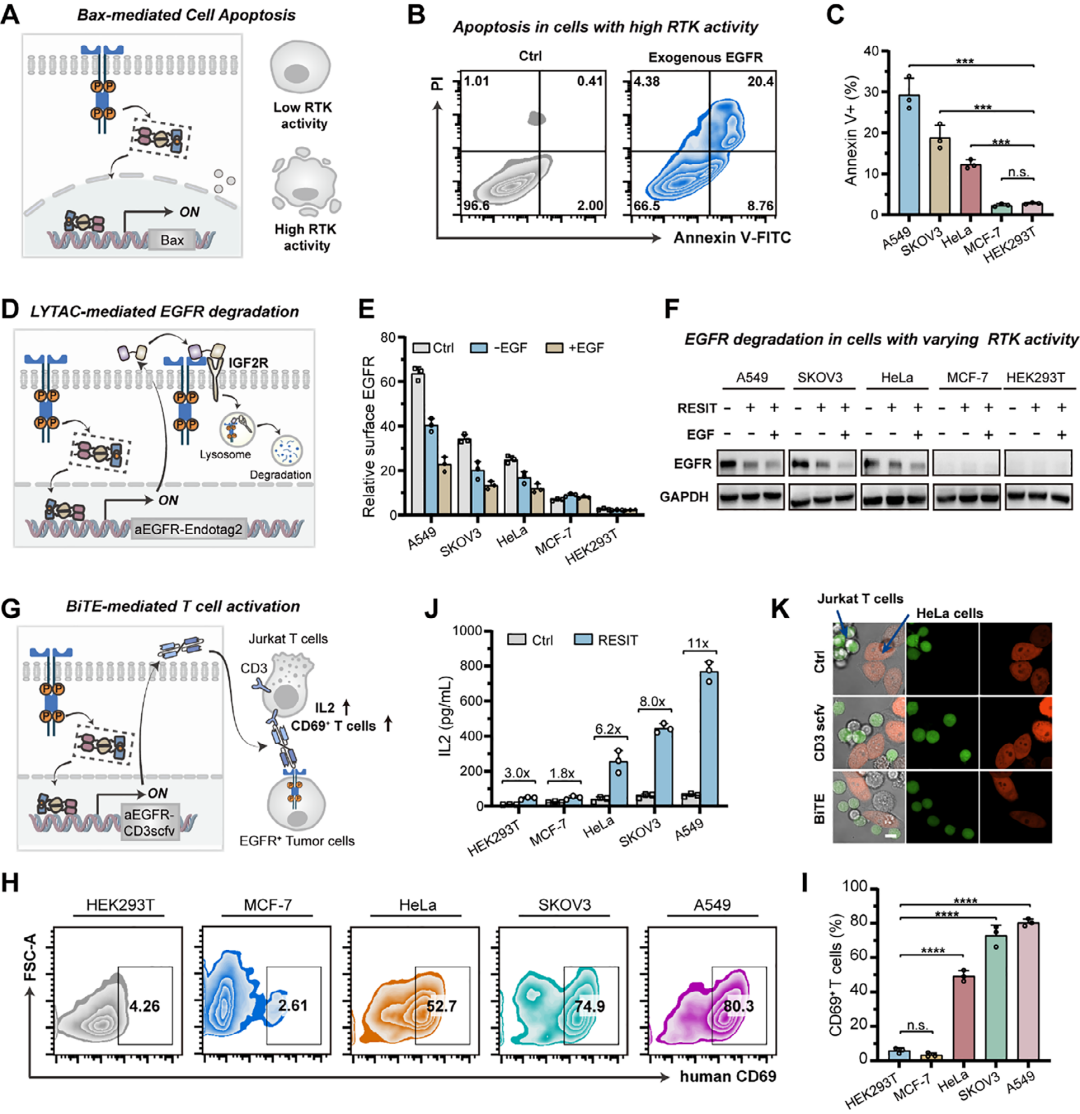
Leveraging the RESIT system to reprogram oncogenic RTK activity for cell‐specific therapy. (A) Schematic for the RTK‐responsive RESIT system using hBax as the effector for apoptosis induction. High RTK activity induced expression of hBax by the RESIT transcriptional circuit resulted in specific apoptosis. (B) Flow cytometry profiles for HEK293T cells expressing the RESIT using hBax as the effector with or without co‐expressing EGFR co‐stained with annexin V‐FITC and propidium iodide (PI). (C) Percentages of Annexin V (+) cells for different cells expressing the RESIT co‐stained with annexin V‐FITC and PI. The significance of differences in different cells vs. HEK293T cells was determined using single‐factor ANOVA (Left to right: ^***^ *p* = 0.0004, ^***^ *p* = 0.0008, ^***^ *p* = 0.0001, *p* = 0.1155). (D) Schematic for RTK‐responsive RESIT system using LYTAC as the effector for EGFR degradation. Activation of the transcriptional circuit in cells with high RTK activity induced the expression and secretion of LYTAC, which mediated EGFR degradation. (E) Levels of surface EGFR for different cells expressing the RESIT using LYTAC as the effector with or without EGF (50 ng/mL) induction. The cells under different treatments were stained by PE‐labeled anti‐EGFR antibody. (F) WB analysis of EGFR levels in different cell lines expressing the RESIT using LYTAC as the effector with or without EGF (50 ng/mL) induction. (G) Schematic for RESIT system responsive to RTK using BiTE as the effector for activation of Jurkat T cells. Activation of the transcriptional circuit in cells with high RTK activity induced the expression and secretion of BiTE, which mediated Jurkat T cells activation. (H) Flow cytometry profiles for Jurkat T cells co‐cultured with different cell lines expressing the RESIT system using BiTE as the effector stained by APC‐labeled anti‐CD69 antibody. (I) Percentages of CD69^+^ T cell in cells under different treatments in (H). The significance of differences in different cells vs. HEK293T cells was determined using single‐factor ANOVA (Left to right: *p* = 0.0817, ^****^ *p* < 0.0001). (J) IL2 levels for Jurkat T cells co‐cultured with different cell lines expressing the RESIT system using BiTE as the effector as determined by enzyme‐linked immunosorbent assay (ELISA). (K) Representative confocal images of Jurkat T cells co‐cultured with HeLa cells expressing the RESIT system using BiTE or scfv for CD3 as the effector. HeLa cells were indicated by orange fluorescence from mCherry. Jurkat T cells were stained with CellTracker green dye (CMFDA). Scale bar, 10 µm. (B, C, E, F, H, I, G, K) Data were represented as mean ± s.d. of three independent measurements.

Constitutive activation of RTK was a key hallmark in a large fraction of cancers. We then re‐engineered the RTK‐responsive RESIT system to sense aberrant RTK activity and repurpose it as a therapy program. We first reprogramed the RTK‐response RESIT circuit into cell death triggers by substituting an apoptosis‐inducing protein Bax for the iRFP reporter (Figure 5A). We initially used this system in EGFR‐overexpressed HEK293T cells by exogenous transfection of an EGFR plasmid vs. normal HEK293T cells with low basal EGFR expression. It was clear that RESIT induced substantial apoptosis in EGFR‐overexpressed cells (Annexin V+, 29.1%) as compared to normal HEK293T cells (Annexin V+, 2.41%) (Figure 5B). This result demonstrated the ability of the RTK‐responsive RESIT system to trigger apoptosis in cells with high RTK activity. We then tested the RESIT circuit for apoptosis induction specifically in cells with aberrant RTK activity. As expected, A549 and SKOV3 cells with high RTK activity showed large extent of apoptosis (Annexin V+, 27.7% and 18.6%), HeLa cells with moderate RTK activity displayed remarkable apoptosis (Annexin V+, 12.3%) and MCF‐7 cells with low RTK activity exhibited little apoptosis (Annexin V+, 2.09%) (Figures 5C; S39). The extents of induced cell apoptosis were consistent with the WB data for RTK activities in these cell lines (Figure 3H), verifying the ability of the RTK‐responsive RESIT system for exerting cell‐specific cytotoxicity.

We then utilized the RTK‐responsive RESIT system to secrete a bispecific lysosome‐targeting chimera (LYTAC) for target protein degradation in cells with high RTK activity (Figure 5D). We replaced the iRFP reporter by a secreting bispecific lysosome‐targeting chimera (LYTAC), which comprised an EGFR‐targeting nanobody and a computationally‐designed peptide binder, Endotag2 [40], toward insulin like growth factor 2 receptor (IGF2R), with a fused secreting luciferase indicator for LYTAC quantification. This system was applied to EGFR degradation in different cells. As anticipated, A549 cells and SKOV3 cells with the highest RTK activity resulted in the most secretion of LYTAC, accompanied with maximized decrease of EGFR on cell surface (Figures 5E; S40). A control experiment with endocytosis inhibitor showed restored EGFR on cell surface, suggesting that EGFR decrease on cell surface was ascribed to endocytosis‐mediated degradation (Figure S41). Upon EGF induction, we obtained substantial EGFR degradation up to 70% in A549 cells and up to 65% in SKOV3 cells, respectively (Figure 5E,F). Moreover, LYTAC secretion and EGFR degradation decreased in HeLa cells (moderate RTK activity) and became undetectable in MCF‐7 and HEK293T cells (low RTK activity), even under EGF induction. A closer interrogation showed that the EGFR decrease in A549 cells was specific to Endotag2‐incorporated LYTAC, as deletion of Endotag2 in LYTAC led to negligible EGFR degradation (Figure S42). These results demonstrated the capability of RESIT for cell‐specific therapy through evoking target protein degradation programs.

We further exploited the RTK‐responsive RESIT system to activate T cells through bispecific antibody mediated cell‐cell contact. We replaced the iRFP reporter by a secreting BiTE consisting of an EGFR nanobody and CD3 scfv (Figure 5G). Cells with varying RTK activity were transfected with the RESIT system and co‐cultured with Jurkat T cells. As anticipated, the levels of CD69 (activation biomarker) and IL2 were remarkably up‐regulated for T cells co‐cultured with HeLa cells (Figure 5H–J). A control assay for T cells co‐cultured with normal HeLa cells without transfection of RESIT only delivered negligible activation. A closer live‐cell imaging analysis revealed direct interactions between RESIT‐transfected HeLa cells and Jurkat T cells, with no close contact obtained for normal HeLa cells and Jurkat T cells (Figure 5K). Another control with RESIT‐transfected HeLa cells that secreted the CD3‐specific scfv in place of the bispecific antibody, interestingly, resulted in negligible activation of Jurkat T cells, accompanied with no substantial cell‐cell contact (Figure S43). This result indicated that bispecific antibody mediated cell‐cell contact was crucial for T cell activations, ascribed to the likely clustering of T cell receptors in the cell‐cell contact interfaces. Furthermore, we obtained varying extents of Jurkat T cell activation in different cell lines, and decreased T cell activation was obtained with cells having lower RTK activity and EGFR expressions (Figure 5I,J). Collectively, these results demonstrated the potential of the RTK‐responsive RESIT system for executing T cell therapy in tumor‐specific microenvironments.

## 3 Discussion

We developed a RESIT system for sensing membrane‐localized receptor activation and rewiring the juxtamembrane signals to pre‐defined transcriptional programs. We demonstrated that the RESIT system was generally applicable to DBDs from different TFs including Gal4, GCN4 and TetR, and various split viral proteases. The RESIT system was successfully developed for detecting Ca^2+^ entry upon PIEZO1 and T cell activation, probing oncogenic receptor RTK activity, and assessing Ras activation and inhibitors. The versatility of RESIT system was repurposed to induce apoptosis, target protein degradation and T cell activation in cells with high EGFR activities. It was demonstrated that the RESIT design exhibited greatly improved sensitivity and spatial selectivity as compared to the cytosol‐localized counterparts. We showed that a Ca^2^^+^ entry of 0.5 min was capable of triggering a measurable RESIT signal. Nonetheless, it should be aware that RESIT relies on time‐integrated transcription for signal production, which is different from other real‐time based genetically‐encoded calcium indicators [11, 41]. As the Ca^2+^‐responsive RESIT relies on CaM‐M13 interaction for activation, it is expected that its sensitivity could be tuned by choosing CaM‐M13 pairs with different affinities, similar to previously reported dimerization mediated signal production designs [42, 43].

The RESIT system relies on receptor activation mediated proximity of interacting domains for signal activation. In theory, RESIT can be engineered to sense other receptor activation receptors (e.g. GPCR and T cell receptors) that involve protein‐protein interaction for signal transduction [44, 45]. The choose of interacting domains is essential to design RESIT with high sensitivity and specificity. To enhance sensitivity and specificity, the engineered substrate modules should be recruited to the close proximity to the receptor. Besides, with the availability of interacting domains for other signaling molecules (e.g. ATP), we expect that RESIT can be possibly engineered to sense the activation of ATP‐gated ion channels [12, 46].

For engineering cell‐based therapy, RESIT that can be activated by bioorthogonal stimuli can minimize effects on endogenous signaling pathways. We showed that the RESIT design was applicable to different chemically dimerization systems, allowing specific activation by three bioorthogonal inducers. With the three engineered orthogonal TFs, it is possible to design a multiplexed RESIT system to achieve selective activation of three distinct outputs by three different inputs within an engineered cell [30, 47, 48].

Overall, the modularity and general applicability of the RESIT system could expand our arsenal for interrogating membrane‐proximal receptor signaling and rewiring dysregulated receptor activation to powerful therapeutic programs.

## 4 Experimental Section

### 4.1 Flow Cytometry Assay

To determine the responses of RESIT to AP21967, cells were transfected with the RESIT system with different tGal4 variants and incubated with AP21967 (1 µm) for 24 h. To determine dose‐dependent response of the RESIT system, the cells were treated with different concentrations of AP21967 for 24 h. To determine the ability of RESIT for detecting RTK activities, transfected cells were treated with EGF (50 ng/mL) for 24 h. To inhibit basal EGFR activity, cells were treated with lapatinib (0.5 µm) upon transfection. For detecting Ras activity, transfected cells were incubated with EGF (50 ng/mL) for 24 h. For evaluating the efficacy of Ras inhibitors, cells transfected with RESIT with different Ras mutants were treated with different concentrations of MRTX1133 or RMC‐7977 for 24 h. For determining the ability of RESIT in detecting Ca^2+^ influx upon PIEZO1 activation, the transfected cells were treated with Yoda1 (10 µm). To determine the ability of RESIT in detecting Ca^2+^ entry across the plasma membrane evoked by thapsigargin mediated Ca^2+^ releasing from ER, the transfected cells were treated with thapsigargin (100 nm), thapsigargin (100 nm) and the channel inhibitor CM4620 (1 µm) or thapsigargin (100 nm) in Ca^2+^ free medium. To determine the ability of RESIT for detecting histamine‐evoked Ca^2+^ entry, MCF‐7 cells with or without exogenous transfection of histamine H1 receptor (H1R) were treated with histamine (100 µm), histamine and the channel inhibitor CM4620 (1 µm) or histamine in Ca^2+^ free medium. To determine the ability of the RESIT in quantitatively detecting Ca^2+^, the transfected cells were treated with different concentrations of Ca^2+^ in the presence of ionomycin for 24 h. To determine the ability of RESIT in detecting Ca^2+^ influx associated with T cell activation, Jurkat T cells were transduced with RESIT for 48 h, and co‐cultured with target cells with varied expressions of HER2 in the presence of a bispecific T cell engager (BiTE) consisting of an HER2 nanobody and a CD3‐specific single‐chain variable fragments (scfv) for another 24 h. To determine the ability of RESIT for EGFR degradation, the transfected cells were treated with EGF (50 ng/mL) for 24 h. To determine the ability of RESIT for Jurkat T cell activation, the transfected cells were co‐cultured with Jurkat T cells for another 24 h.

To determine the levels of EGFR on the cell surface, different cell lines were stained with PE‐labeled anti‐human EGFR antibody. The cells were washed three times with PBS, detached with trypsin and washed twice with PBS. The cells were resuspended in 100 µL of the staining buffer prepared by mixing 1 µL of antibody with 100 µLof PBS (containing 5% FBS) and then incubated on ice in the dark for 20 min. The cells were then washed twice with the staining buffer by centrifugation at 350×g for 5 min before flow cytometry analysis. To determine the levels of CD69 on Jurkat T cells, Jurkat T cells under different treatments were stained with APC‐labeled anti‐human CD69 antibody. To determine the levels of HER2 on target cell lines, K562 cells with different HER2 densities, MDA‐MB‐468 and SKOV3 were stained with PE anti‐human CD340 (erbB2/HER‐2).

For cell apoptosis analysis, HEK293T, SKOV3 and A549 cells were seeded in 24‐well plates and grown to a confluence of 50–70%. Cells were transfected with the RTK‐responsive RESIT with hBax as the effector using Lipofectamine 3000 in Opti‐MEM medium. The culture medium was changed to complete medium at 6 h post transfection and cultured for another 24–48 h. The cells were washed three times with PBS, detached with trypsin and washed twice with PBS. The cells were incubated with annexin V‐FITC (2 µL per million cells) and propidium iodide (PI, 2 µL per million cells) in annexin V binding buffer (1 ×, 100 µL) for 15–20 min at room temperature in the dark according to the manufacturer's instructions (Elabscience). Annexin V binding buffer (1 ×, 400 µL) were added to each tube for flow cytometry analysis.

Cells under different treatments were washed three times with PBS, detached with trypsin and washed twice with PBS. The cells were suspended in PBS containing EDTA (2 mM) and bovine serum albumin (5%). Approximately 1 × 10^4^ live cells from each sample were analyzed using a FACS Celesta Flow Cytometer (BD Biosciences). Fluorescence data were acquired with the following cytometer settings: 405 nm laser with a 450/40 nm bandpass filter for BFP, 488 nm laser with a 530/30 nm bandpass filter for EGFP, 561 nm laser with a 610/20 nm bandpass filter for mCherry, 633 nm laser with a 780/60 nm bandpass filter for iRFP713. Data were compensated and analyzed using FlowJo_V10 software. Live, single cells were gated using forward and side scatter plots (FSC‐A vs. SSC‐A), and forward scatter area and forward scatter height (FSC‐A vs. FSC‐H) to exclude debris and non‐singlet events. For determining RTK‐activities in different cell lines, double‐positive cells were gated based on mCherry and EGFP fluorescence, and BFP was used to normalize the expression differences among different cells (Figure S44). For cell‐specific therapy with RESIT, positive cells were gated on EGFP, and BFP was used to normalize expression difference except for apoptosis induction in which the cells were gated by BFP. Flow cytometry data were analyzed using FlowJo_V10 software.

### 4.2 Western Blot Analysis

To determine phosphorylation levels of the designed substrate NPXY, HeLa cells were transfected with the RTK‐responsive RESIT with wild‐type PTB, PTB mutants (R175Q or R175K), VAV1 SH2 domain deletion, or the substrate mutation (NPXF). After 24 h, the cells were treated with or without EGF (50 ng/mL) for 30 min. To determine the levels of EGFR or phosphorylated EGFR (p‐EGFR) in different cells, A549, SKOV3, HeLa, MCF‐7 and HEK293T cells were seeded in 24‐well plates and grown to a confluence of 70%–90%. The cells were treated with or without 50 ng/mL EGF for 30 min. For assessing the efficacy of RTK‐responsive RESIT in EGFR degradation, different cell lines (A549, SKOV3, HeLa, MCF‐7 and HEK293T) were transfected with the RTK‐responsive RESIT using LYTAC as the effector. After 24 h, the cells were incubated with EGF (50 ng/mL) for an additional 24 h. For internal control, the housekeeping protein GAPDH was also analyzed. After washing 3 times with ice‐cold PBS, the cells were lysed on ice for 30 min using RIPA buffer comprising 50 mm Tris‐HCl (pH 7.4, 150 mm NaCl, 1% NP‐40, 0.5% sodium deoxycholate, 0.1% SDS, 5 mm EDTA and 1 mm EGTA), 1% protease inhibitor and 1% phosphatase inhibitor. Cell lysates were collected using pre‐cooled scrapers, centrifuged at 15 000 rpm for 20 min to remove cell debris. Protein concentration was determined using bicinchoninic acid assay (BCA) according to the manufacturer's instructions. The protein samples (30 µg) were incubated in 1 × loading sample buffer and denatured at 95°C for 10 min. The samples were analyzed by sodium dodecyl sulfatepolyacrylamide gel electrophoresis (SDS‐PAGE, 10%), and then transferred to a polyvinylidene fluoride (PVDF) membrane at 300 mA for 80 min. The PVDF membranes were blocked with tris‐buffered saline with Tween‐20 containing 5% BSA (1 × TBST) for 2 h at room temperature. The membranes were incubated overnight at 4°C with mouse anti‐human EGFR mAb (1:1000), rabbit anti‐human phospho‐EGFR (Tyr1173, 1:1000) and rabbit anti‐GAPDH mAb (1:1000). After washing with 1 × TBST for three times, the membranes were incubated with HRP‐conjugated goat anti‐mouse IgG antibody (1:4000) and HRP‐conjugated goat anti‐rabbit IgG antibody (1:5000) for 2 h at room temperature. Subsequently, the membranes were washed with 1 × TBST for three times and treated with the chemiluminescent HRP substrate. The protein bands were visualized using the SuperSignal ECL chemiluminescence kit (Biosharp Biotechnology).

### 4.3 Software and Statistics

Graphs and statistical analysis were prepared with GraphPad Prism 8. Values are presented as the means of at least three independent experiments ± standard deviation (s.d.). For statistical analysis, two‐tailed Student's t‐tests or single‐factor analysis of variance (ANOVA) were used to evaluate whether a significant difference exists between two groups of samples. P values: N.S. not significant, *p* ≥ 0.05; ^*^ *p* < 0.05; ^**^ *p* < 0.01; ^***^ *p* < 0.001, ^****^ *p* < 0.0001.

For more experimental details, including the plasmid construction, cell culture and plasmid transfection, lentivirus production and transduction, protein expression and purification, live cell imaging, Enzyme‐linked immunosorbent assay and Gaussia luciferase assay could be found in the Supporting Information.

## Author Contributions

J.H.J. conceived and designed the project. F.L. performed majority of the experiments. M.Y. designed the plasmid constructs. F.L., F. W. and J.H.J. conducted data analysis and interpretation. J.H.J. and F. W. wrote the manuscript and supervised the project. All authors read and approved the final manuscript.

## Funding

This work was supported by NSFC Programs (22322404, 22404050, 22274040, 22374041) and Major Basic Research Project of Hunan Province (No. 2025JC0003).

## Conflicts of Interest

The authors declare no conflicts of interest.

## Supporting information




**Supporting File**: advs74530‐sup‐0001‐SuppMat.docx.

## Data Availability

The authors declare that all relevant data supporting the findings of this study are available within the paper and its supplementary Information files. Any other relevant data are available upon reasonable request. Source data are provided with this paper.
