# Supplementary material for: Sensing and Reprogramming Surface Receptor Activation With Synthetic Transcriptional Circuits
Source: Adv Sci (Weinh). 2026 Feb 23;13(25):e22557. doi: 10.1002/advs.202522557 (PMC13137790; doi:10.1002/advs.202522557)
Supplement: Supplementary file 1 — Supporting File: advs74530‐sup‐0001‐SuppMat.docx. [file ADVS-13-e22557-s001.docx]

**Supporting Information**

**Sensing and Reprogramming Surface Receptor Activation with Synthetic Transcriptional Circuits**

Fei Liu, Mei Yuan, Li-Juan Tang, Fenglin Wang*, Xia Chu, Jian-Hui Jiang*

State Key Laboratory of Chemo and Biosensing, College of Chemistry and Chemical Engineering, Hunan University, Changsha, Hunan 410082, China

*Email: fengliw@hnu.edu.cn
*Email: jianhuijiang@hnu.edu.cn

Table of contents

[I. Materials and Methods S3](#_Toc220068365)

[II. Supplementary Figures S7](#_Toc220068366)

[III. Supplementary Tables S51](#_Toc220068367)

[IV. References S53](#_Toc220068368)

# I. Materials and Methods

**Materials and reagents.** HEK293T (human embryonic kidney cell line), HeLa (human cervical carcinoma cell line), MCF-7 (human breast cancer cell line) and A549 (human lung cancer cell line) cells were supplied by the cell bank of Central Laboratory at Xiangya Hospital (Changsha, China). SKOV3 (human ovarian cancer cell line), MDA-MB-468(human breast cancer cell line), K562 (human chronic myelogenous leukemia cell line) and Jurkat T cells (human T-cell leukemia cell) were purchased from Pricella (Wuhan, China). Dulbecco’s modified Eagle’s medium (DMEM), 100% heat-inactivated fetal bovine serum, penicillin, streptomycin, 0.25% trypsin-EDTA (1×), Opti-MEM I and Lipofectamine 3000 were obtained from Thermo Scientific HyClone (MA, USA). Dulbecco’s modified Eagle’s medium (DMEM) without Ca^2+^ (YC-2067) was obtained from Yuchun Biology (Shanghai, China). Roswell Park Memorial Institute (RPMI-1640) medium and McCoy's 5A medium were purchased from Pricella (Wuhan, China). 1×Phosphate buffered saline (PBS) was purchased from Servicebio (Wuhan, China). Calcium chloride (T64679), ionomycin ([T7285](https://www.tsbiochem.com/compound/ionomycin)), Yoda 1 ([T7506](https://www.tsbiochem.com/compound/yoda%201)), histamine (T0965), epidermal growth factor ([T8679](https://www.tsbiochem.com/compound/epidermal%20growth%20factor)) abscisic acid ([T](https://www.tsbiochem.com/compound/ionomycin)6368) and danoprevir ([T6025](https://www.tsbiochem.com/compound/yoda%201)) were purchased from Target Mol (Shanghai, China). MRTX1133 (HY-134813), RMC-7977 (HY-156498), Thapsigargin (HY-13433), CM-4620 (HY-101942), lapatinib (HY-50898) and chlorpromazine (HY-12708) were obtained from [MedChemExpress.](mailto:sales@MedChemExpress.cn) Annexin V-FITC/PI apoptosis kit (E-CK-A211) was purchased from Elabscience (Wuhan, China). Gaussia luciferase reporter gene assay kit (RG072), goat anti-mouse HRP-conjugated IgG antibody (A0216) and goat anti-rabbit HRP-conjugated IgG antibody (A0208) were obtained from Beyotime (Shanghai, China). Human IL-2 ELISA kit (88-7025) and PE-labeled anti-human EGFR antibody ([MA5-28544](https://www.tsbiochem.com/compound/epidermal%20growth%20factor)) were purchased from Thermo Scientific HyClone (MA, USA). Mouse anti-human EGFR mAb (66455-1-lg) was purchased from Proteintech. Rabbit anti-human phospho-EGF receptor (Tyr1173) (4407) and GAPDH (2118) mAbs were purchased from Cell Signaling Technology. PE anti-human CD340 (erbB2/HER-2) (324405) and APC-labeled anti-human CD69 antibody ([310910](https://www.tsbiochem.com/compound/epidermal%20growth%20factor)) were purchased from BioLegend.

**Plasmid construction.** All the plasmids were constructed using standard molecular cloning or Gibson assembly. Primers for cloning were obtained from Sangon Biotech and conventional PCR reactions were performed using PrimeSTAR Max DNA Polymerase from Takara. Sizes of PCR products were verified using standard agarose gel electrophoresis. All plasmids were amplified in the E. coli strain, grown at 37 °C and extracted using the EndoFree Mini Plasmid Kit (TIANGEN) according to the manufacturer’s protocol. DNA sequences were validated by Sanger sequencing (Sangon Biotech), and DNA was stored at −20 °C until further use. The amino sequences for main RESIT domains were provided in Table S1.

Plasmids for expressing the RESIT system were constructed using a mammalian expression vector pcDNA3.1(+). To construct plasmid N-myr−tGal4(65)−FKBP−nTEVp for expressing one of the membrane-localized transcriptional modules, cDNAs of N-myristoylation signal, tobacco etch virus protease (TEVp) cleavage site (TCS), DNA-binding domain (DBD) from truncated Gal4 (tGal4(65); 1-65 aa),^[1]^ N-terminal of TEVp (nTEVp, 1-117 aa), ^[2]^ VP64, FK506-binding protein (FKBP) and EGFP were obtained by overlap PCR and cloned into NheI and XbaI sites under the CMV promoter. To construct PH−tGal4(65)−cTEVp−FRB^T2098L^ for expressing the other membrane-localized transcriptional module, cDNAs of the pleckstrin homology (PH) domain of phospholipase C delta 1 (1-175 aa),^[3]^ TCS, tGal4(65), C-terminal of TEVp (cTEVp, 121-218 aa)_,_ FRB^T2098L^ and BFP were obtained by overlap PCR and cloned into NheI and XbaI sites under the CMV promoter. Plasmids for expressing membrane-localized transcriptional modules with different fusion combinations were constructed similarly. Plasmids for expressing with different tGal4 variants were obtained using site-directed mutation. Reporter or effector plasmids under a miniCMV promoter were constructed with a pcDNA3.1(+) vector. cDNAs of miniCMV promotor, Gal4-specific binding sequence (5×UAS) and iRFP670, Gluc, hBax, aEGFR−Endotag2 (LYTAC) or aEGFR−CD3 scfv (BiTE) were obtained by overlap PCR and cloned into the MluI and XbaI sites.

To construct plasmids for expressing RTK-responsive RESIT system, DNA sequences for PTB obtained by overlap PCR, amplified and cloned into the backbone of N-myr−tGal4(65)−cTEVp−FKBP digested by restriction enzymes AfeI and AgeI to generate plasmid N-myr−tGal4(65)−cTEVp−PTB. Similarly, plasmid PH−VAV1−tGal4(65)−nTEVp−substrate was constructed by cloning amplified VAV1 and substrate into plasmid PH−tGal4(65)−nTEVp−FRB^T2098L^. Plasmids for expressing RESIT with different PTB mutants (R175Q or R175K), substrate mutation (NPXF), SH2 or VAV1 deletion were constructed via site-directed mutation. To construct plasmids expressing RESIT responsive to Ras, Ca^2+^, danoprevir or abscisic acid, DNA sequences for Ras, M13, DNCR2 or ABI were obtained, amplified and cloned to the backbone of N-myr−tGal4(65) −cTEVp−FKBP digested by restriction enzymes AfeI and AgeI to obtain plasmid N-myr−tGal4(65)−cTEVp−Ras, N-myr−tGal4(65)−cTEVp−M13, N-myr−tGal4(65)−cTEVp−DNCR2 or N-myr−tGal4(65)−cTEVp−ABI. Plasmids for expressing RESIT system with different fusion combination modules were obtained similarly. Plasmids for expressing RESIT system without the membrane tether or with different Ras mutants (G12C, G12D, G13D or Q61L) or Raf mutant (R89L) were constructed via site-directed mutation. To construct plasmids expressing RESIT system responsive to Ca^2+^ influx in T cells, DNA sequences for N-myr−tGal4(65)−cTEVp−M13 and PH−tGal4(65)−nTEVp−CaM were obtained, amplified and cloned to the backbone of a dual-promoter lentiviral vector, with the former under the control of the CMV promoter and the latter under the control of the EF1α promoter. Gluc reporter plasmids under a miniCMV promoter were also constructed in a lentiviral vector.

To construct plasmids for RESIT system using the DBD of truncated TetR (tTetR) ^[4, 5]^ or truncated GCN4 (tGCN4),^[6]^ DNA sequences for tGCN4(274) or tTetR(196) were obtained by conventional PCR and cloned to the backbones of plasmid N-myr−tGal4(65)−FKBP−nTEVp and PH−tGal4(65)−cTEVp−FRB^T2098L^ digested by restriction enzymes BsiWI and EcoRV. Plasmids for expressing RESIT system with different tTetR and tGCN4 variants were obtained using site-directed mutation. Reporter plasmid for RESIT system using DBD of tTetR and tGCN4 under a miniCMV promoter were constructed with a pcDNA3.1(+) vector. cDNAs of TetR-specific binding sequence (7×Teto) or GCN4-specific binding sequence (5×AP1), miniCMV promotor and mCherry were obtained by overlap PCR and cloned into the MluI and XbaI sites. Teto nucleotide sequence, 5'-TCCCTATCAGTGATAGAGA-3'. AP1 nucleotide sequence, 5'-TTCCTATGACTCATCCAGTTT-3'.

**Cell culture and plasmid transfection.** HEK293T, HeLa and MCF-7 cells were cultured in DMEM supplemented with 10% fetal bovine serum (FBS), penicillin (100 U/mL) and streptomycin (100 U/mL). MDA-MB-468, K562, A549 and Jurkat T cells were cultured in RPMI-1640 medium supplemented with 10% FBS, penicillin (100 U/mL) and streptomycin (100 U/mL). SKOV3 were cultured in McCoy's 5A medium supplemented with 10% FBS, penicillin (100 U/mL) and streptomycin (100 U/mL). All the cells were incubated at 37 °C in a humidified incubator containing 5% CO_2_. For fluorescence imaging, the cells were plated on sterilized glass coverslips in 35 mm plates with 14 mm well and grown to a confluence of 50–70% in the corresponding medium at 37°C in a humidified atmosphere containing 5% CO_2_. For flow cytometry analysis, cells were seeded in 24-well plates and grown to a confluence of 50-70%.

The cells were transfected with a mixture of plasmids and Lipofectamine 3000 (0.5 μL/plasmid) in Opti-MEM medium according to the manufacturer’s instructions. The culture medium was changed to complete medium 6 h after transfection and cultured for another 24 h before further processing. To express the AP21967-responsive RESIT system, cells were transfected with 60 ng of tGal4(65)−FKBP−nTEVp module and 180 ng of tGal4(65)−cTEVp−FRB^T2098L^ module. To express RESIT system using the DBD of truncated TetR (tTetR), cells were transfected with 60 ng of tTetR(196)−FKBP−nTEVp module and 120 ng of tTetR(196)−cTEVp−FRB^T2098L^ module. For expressing RESIT system using the DBD of truncated GCN4 (tGCN4), cells were transfected with 40 ng of tGCN4(274)−FKBP−nTEVp module and 120 ng of tGCN4(274)−cTEVp−FRB^T2098L^ module. To express DNV-responsive RESIT, cells were transfected with 60 ng of tGal4(65)−cTEVp−DNCR2 module and 180 ng of tGal4(65)−nTEVp−NS3a module. To express ABA-responsive RESIT, cells were transfected with 60 ng of tGal4(65)−cTEVp−ABI module and 180 ng of tGal4(65)−nTEVp−PYL1 module. To express the Ca^2+^-responsive RESIT system, cells were transfected with 60 ng of tGal4(65)−cTEVp−M13 module and 180 ng of tGal4(65)−nTEVp−CaM module. For expressing the RTK-responsive RESIT system, cells were transfected with 60 ng of tGal4(65)−cTEVp−PTB module and 180 ng of VAV1−tGal4(65)−nTEVp-substrate module. To express the RESIT system responsive to RAS activity, cells were transfected with 60 ng of the tGal4(65)−cTEVp−Ras module and 180 ng of the tGal4(65)−nTEVp−RBD module.

For transcription actuation of reporters, cells were transfected with 10 ng of the 5×UAS−iRFP670, 5×UAS−Gluc, 7×Teto−mCherry or 5×AP1−iRFP670 reporter plasmid. To induce apoptosis, cells were transfected with 50 ng of the 5×UAS−hBax effector module. For EGFR degradation via LYTAC, cells were transfected with 100 ng of the 5×UAS−aEGFR−Endotag2 effector plasmid. To activate T cells via BiTE, cells were transfected with 10 ng of the 5×UAS−aEGFR−CD3scfv effector module. For transfection cells of varying numbers, the amount of each plasmid was adjusted to the cell counts proportionally.

**Lentivirus production and transduction.** HEK293T cells were plated in 15-cm dishes for 18 h to achieve ~70% confluence. The cells were co-transfected with a vector encoding the extracellular and transmembrane region of human HER2 (aa: 23-675) fused with an intracellular blue fluorescent protein under an SFFV promoter (15 μg), a lentiviral package vector (psPAX2, 15 μg; Addgene #12260) and a vector encoding vesicular stomatitis virus glycoprotein G pseudotyping coat protein (pMD2.G, 7.5 μg; Addgene #12259) using PEI (8 μL per 1 μg plasmids) in Opti-MEM medium. The culture medium was replaced with complete medium 6 h after transfection. The viral supernatant was collected at 48 h post-transfection, filtered through a syringe filter (0.45 μm, Pall Corporation) and centrifuged at 25,000 g for 1.5 h at 4 °C. The viral particles were resuspended in PBS (200 μL) and stored at −80 °C before use.

To establish K562 cells with stable HER2 expressions, K562 cells were plated in six-well plates in complete medium, added with polybrene (8 μg/μL, Sigma-Aldrich) and infected with 100 μL of virus-containing medium with a multiplicity of infection of ~3. The cells were sorted with a Bigfoot cell sorter (Thermo Fisher Scientific) on the basis of HER2 expression and subsequently expanded. The HER2 densities were determined by flow cytometry using PE-labeled anti-HER2 antibody (Biolegend). The engineered cells were cultured in RPMI media supplemented with 10% FBS and gentamicin.

**Protein expression and purification.** CD3 scfv-HER2 nanobody were expressed in *E. coli* BL21(DE3). In brief, the DNA fragments encoding the design sequences were assembled into pET24a vectors and further transformed into BL21 strain with heat-shock. Protein expression was induced by IPTG (0.5 mM) and proteins were purified with His-tag Purification Resin. The eluents were subjected to PBS dialysis. Protein concentrations were determined by NanoDrop (Thermo Scientific) and normalized by extinction coefficients. Protein sequences were provided in Table S1.

**Live cell imaging.** Cells (5 ×10^5^) were seeded onto sterilized glass coverslips in 35-mm plates with 14-mm wells. At a confluence of 50-70%, cells expressing RESIT system were obtained by transfection with a mixture of plasmids for 6 h. For evaluating the performance of RESIT system via fluorescence imaging, the transfected cells were treated similarly as that described in the flow cytometry assay section. To determine the ability of the RESIT system for Jurkat T cell activation via cell-cell interaction, the transfected HeLa cells were co-cultured with Jurkat T cells stained with CellTracker green dye (CMFDA) (1 μM) for another 12 h.

Cells under different treatments were washed with PBS and incubated with DMEM medium. The cells were washed and incubated in a fresh medium before imaging. All fluorescence images were collected with a Nikon TI-E+A1 SI confocal laser scanning microscope using an oil immersion objective lens of 60× or a dry objective lens of 20×. Fluorescence images were collected using the following collection channels upon excitation with lasers of different wavelengths. For blue channel, the samples were excited with a 405 nm laser, and signals were collected in the range of 425 - 475 nm. For green channel, the samples were excited with a 488 nm laser, and signals were collected in the range of 500 - 550 nm. For orange channel, the samples were excited with a 560 nm laser, and signals were collected in the range of 575 - 625 nm. For red channel, the samples were excited with a 640 nm laser, and signals were collected in the range of 650 – 700 nm. For fluorescence intensity analysis, the fluorescence signals of at least 20 cells from three independent experiments were analyzed using ImageJ.

**Enzyme-linked immunosorbent assay.** Jurkat T cells were cocultured with cells expressing the RTK-responsive RESIT system using BiTE as the effector for 48 h. The concentrations of interleukin 2 (IL-2) in the culture supernatant were quantified using a human IL-2 uncoated ELISA kit (Invitrogen). Particularly, anti-human IL-2 antibody (capture antibody) is coated to the well bottom of microplates and incubated overnight at 4°C. The wells were washed for three times with washing buffer (0.1% Tween 20 in PBS 7.4). The wells were blocked with ELISA/ELISPOT diluent (1×, 200 µL) at room temperature for 1 h. After washing for three times, cell supernatants with appropriate dilution, varying concentrations of standard IL-2, or blank control were added into the wells and incubated at room temperature for 2 h. The wells were washed for 3 times and added with biotin-conjugated anti-human IL-2 antibody at room temperature for 1 h. The wells were washed again and incubated with avidin-HRP for 0.5 h at room temperature in the dark. After washing for 5 times, the wells were developed using tetramethylbenzidine for 15 min in the dark and quenched by 1 M H_3_PO_4_. Absorbance at 450 nm was measured using a microplate reader (Molecular Devices) at 37 °C. The concentrations of IL2 were calculated based on the standard curve.

**Gaussia luciferase assay.** For RESIT-mediated Gluc secretion, cells were transfected with the AP21967 responsive RESIT system using Gluc as the reporter according to the transfection procedure described in the cell culture and plasmid transfection section. After 24 h, the cells were incubated with AP21967 (1 μM) for 24 h. For determining the secretion of Gluc in T cells, Jurkat T cells were transduced with RESIT system for 48 h, and co-cultured with target cells with varied expressions of HER2 in the presence of a bispecific T cell engager (BiTE) consisting of an HER2 nanobody and a CD3scfv for another 24 h. For determining the secretion of Gluc-LYTAC, different cell lines (A549, SKOV3, HeLa, MCF-7 and HEK293T) were transfected with the RTK-responsive RESIT system using Gluc-LYTAC as the effector. The levels of secreted Gluc in the supernatants were quantified using a Gaussia luciferase assay kit (Beyotime). Particularly, the assay reagent was prepared by combining one volume of Gaussia luciferase assay substrate with 100 volumes of assay buffer. Then, 50 μL of cell culture supernatant was mixed with 50 μL of the assay reagent in a 96-well plate and incubated for 5-10 min at room temperature. Luminescence intensity was quantified using a microplate reader (Molecular Devices).

# II. Supplementary Figures


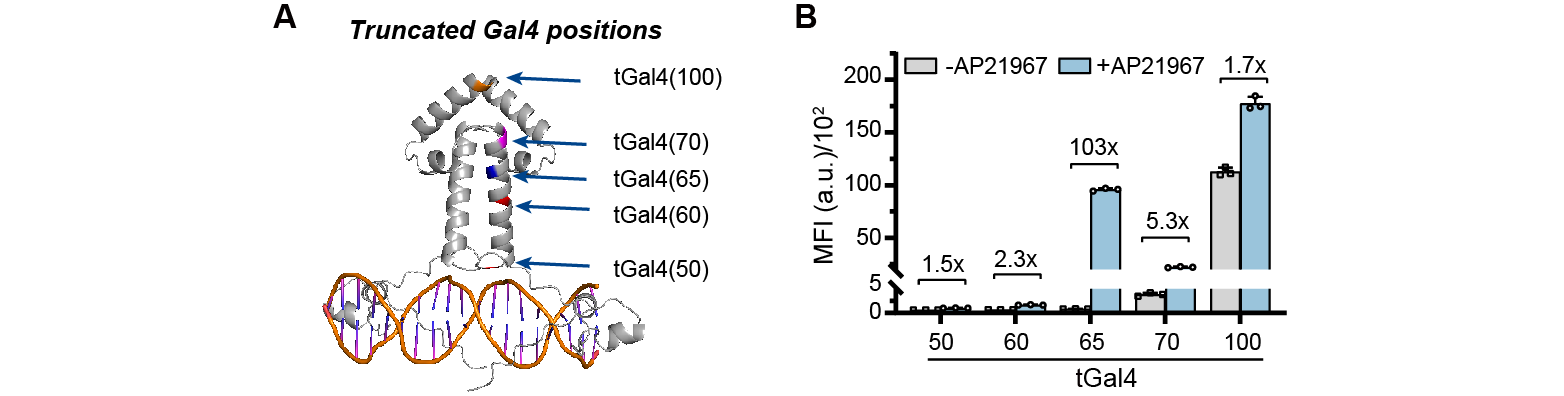


**Figure S1. Evaluating the performance of RESIT system with different truncated Gal4 variants.** (A) The superimposed structure of Gal4(1–101, gray) and DNA (orange) complex (PBD: 3COQ). Blue arrows indicated the truncated Gal4 positions. (B) Optimization of RESIT using different tGal4 variants under AP21967 (1 μM) induction determined by mean fluorescence intensities (MFI) of iRFP670 from flow cytometry profiles. Data were represented as mean ± s.d. of three independent measurements.


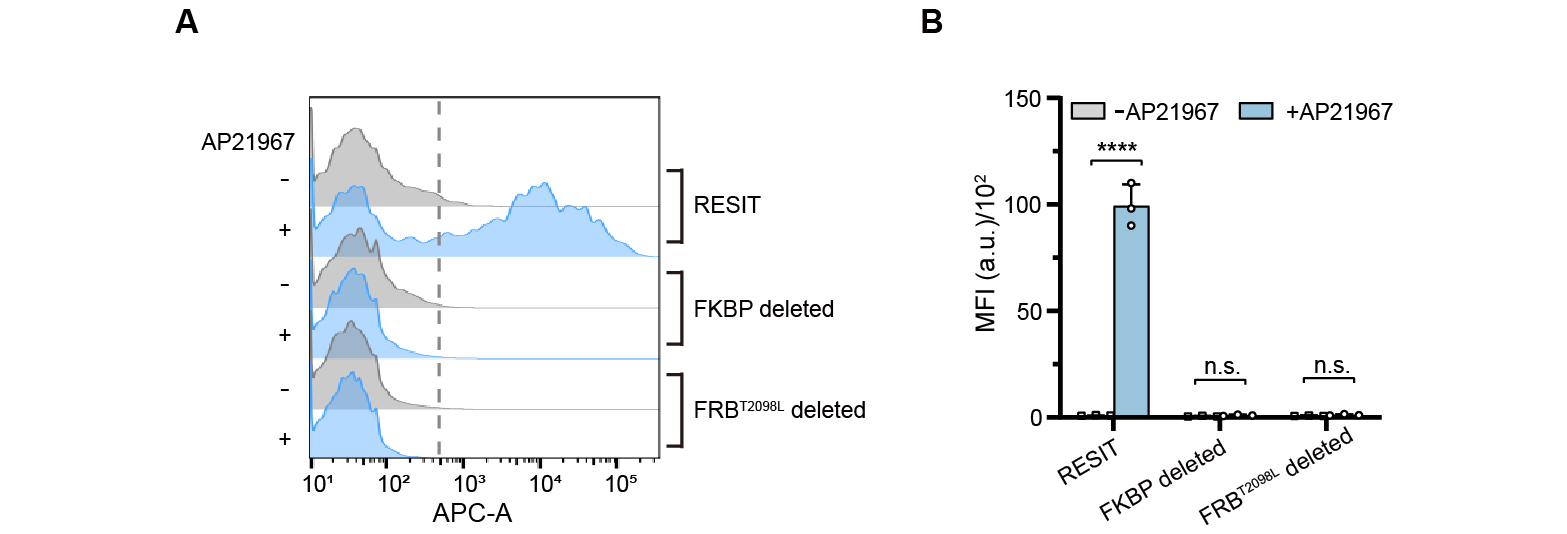


**Figure S2. Specificity of AP21967-responsive RESIT design.** (A) Flow cytometry profiles for HeLa cells expressing the RESIT system with FKBP or FRB^T2098L^ deleted in one transcriptional module with or without AP21967 induction. (B) Mean fluorescence intensity (MFI) of iRFP for cells in (A). Cells expressing the RESIT system with FKBP or FRB^T2098L^ deleted displayed negligible iRFP fluorescence with or without AP21967 induction. Statistical analysis was performed using a two-tailed t-test (Left to right: ****p < 0.0001, p = 0.0996, p = 0.1256). Error bars represented s.d. of three independent experiments.


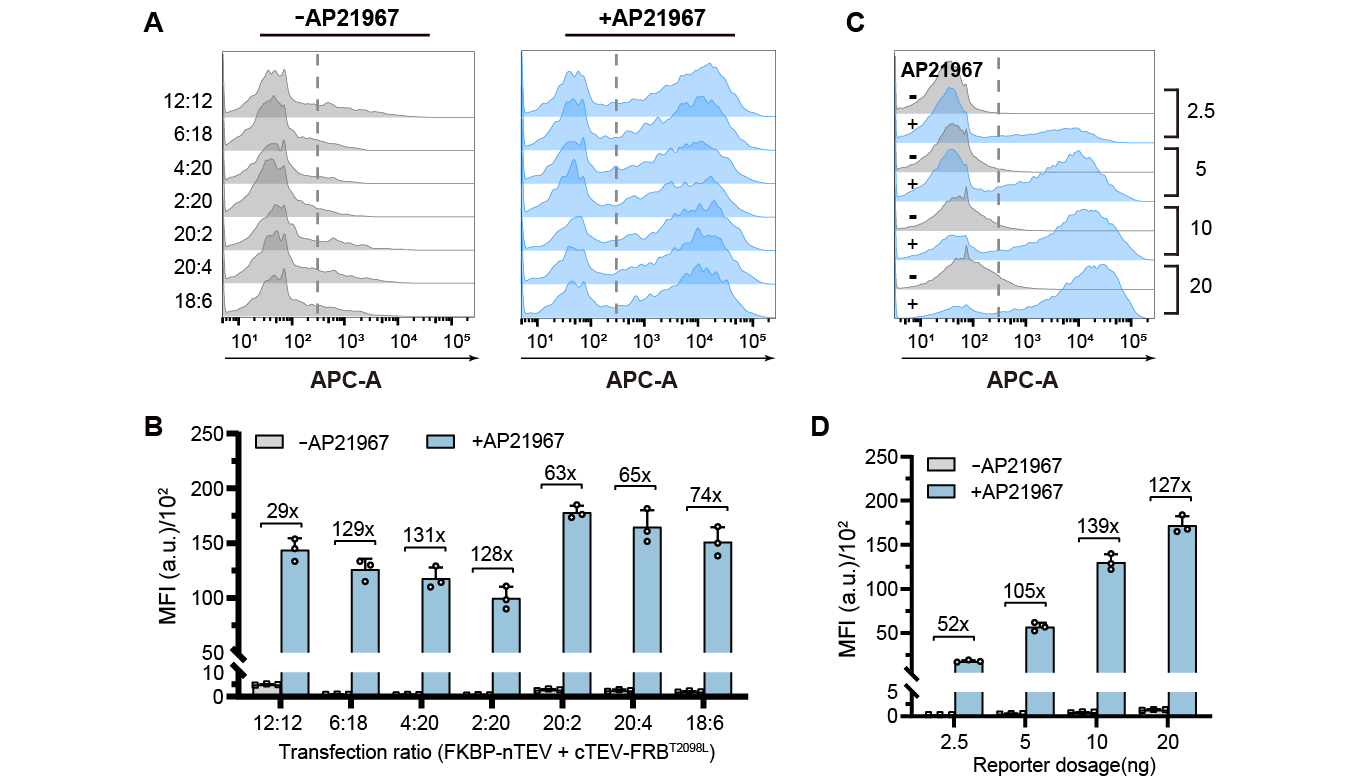


**Figure S3. Optimization the ratios of plasmid amounts for** **the AP21967-responsive RESIT system.** (A) Flow cytometry profiles for HeLa cells transfected with the membrane-tethered transcriptional modules at different ratios with a fixed reporter amount with or without AP21967 (1 μM). (B) MFI of iRFP670 for cells in (A). (C) Flow cytometry profiles for HeLa cells transfected with varying amounts of reporter plasmid while keeping the membrane-tethered transcriptional modules at a ratio of 1:3 with or without AP21967 (1 μM). (D) MFI of iRFP670 for cells in (C). Data were represented as mean ± s.d. of three independent measurements.


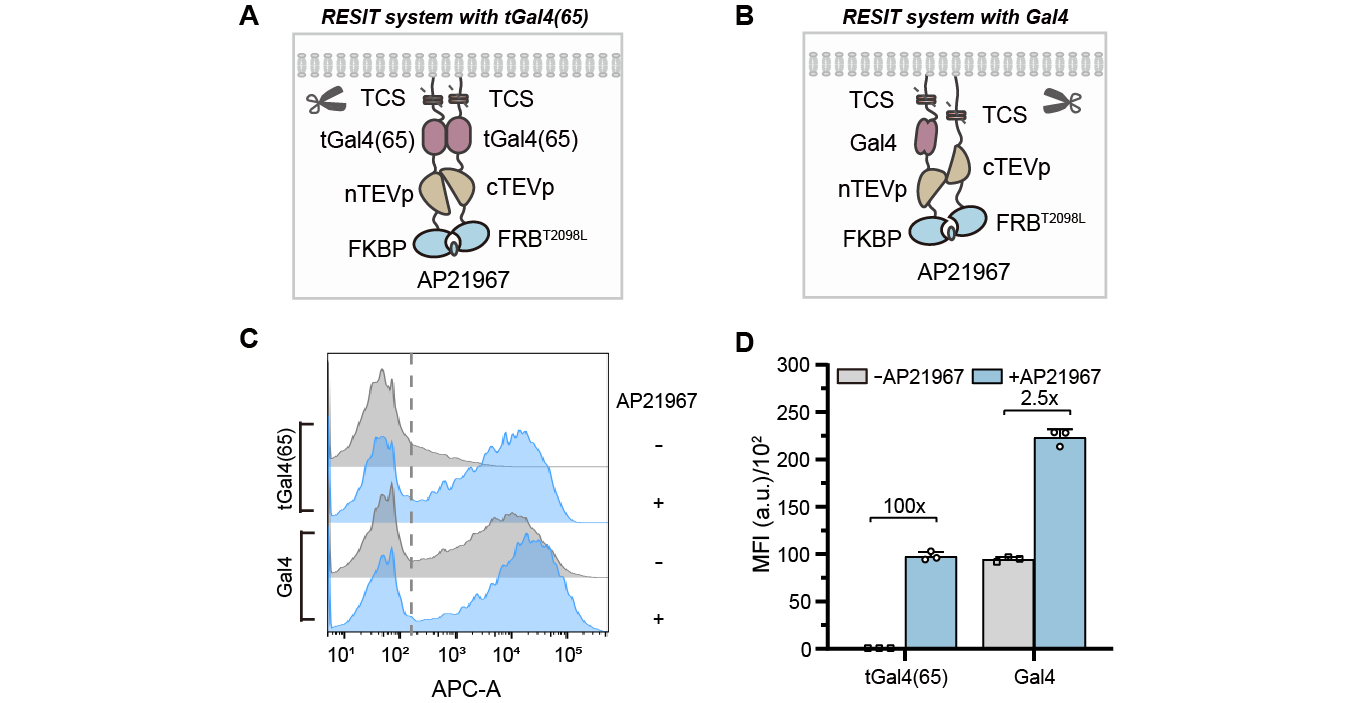


**Figure S4. Comparison of RESIT system with the half DBD tGal4(65) and wild-type Gal4 in the sensing modules.** (A) Schematic of RESIT system with the half DBD tGal4(65) in response to AP21967. (B) Schematic of RESIT system with the wild-type Gal4 in response to AP21967. (C) Flow cytometry profiles for HeLa cells expressing the RESIT system with the half DBD tGal4(65) or wild-type Gal4 with or without AP21967 induction. (D) MFI of iRFP for cells in (C). Cells expressing the wild-type Gal4 displayed high iRFP670 fluorescence background which resulted in low S/B ratio. Error bars represented s.d. of three independent experiments.


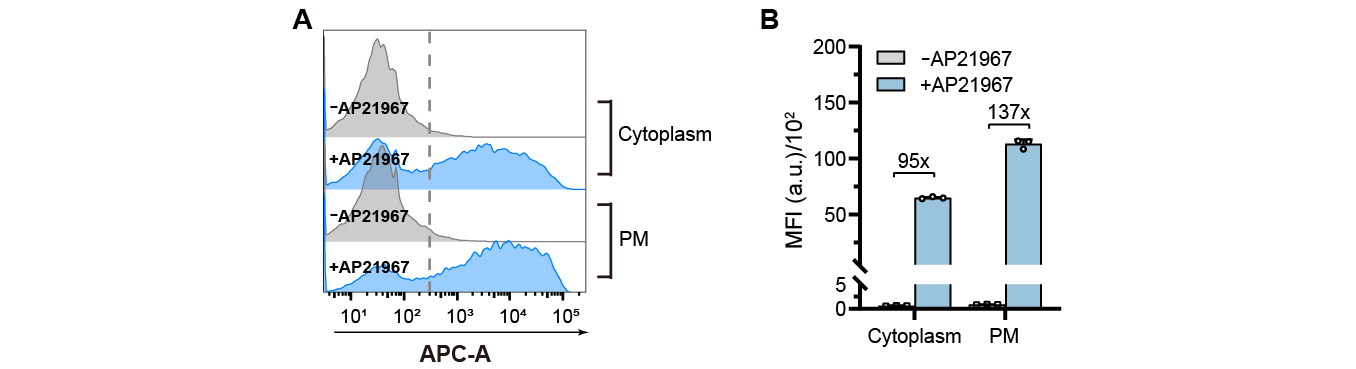


**Figure S5. Comparison between membrane-tethered RESIT (PM) or the cytosol-localized counterpart (Cytoplasm) responsive to AP21967.** (A) Flow cytometry profiles for HeLa cells expressing membrane-tethered RESIT (PM) and cytosol-localized RESIT (Cytoplasm) under AP21967 (1 μM) induction. (B) MFI of iRFP670 for cells in (A). Error bars represented s.d. of three independent experiments.


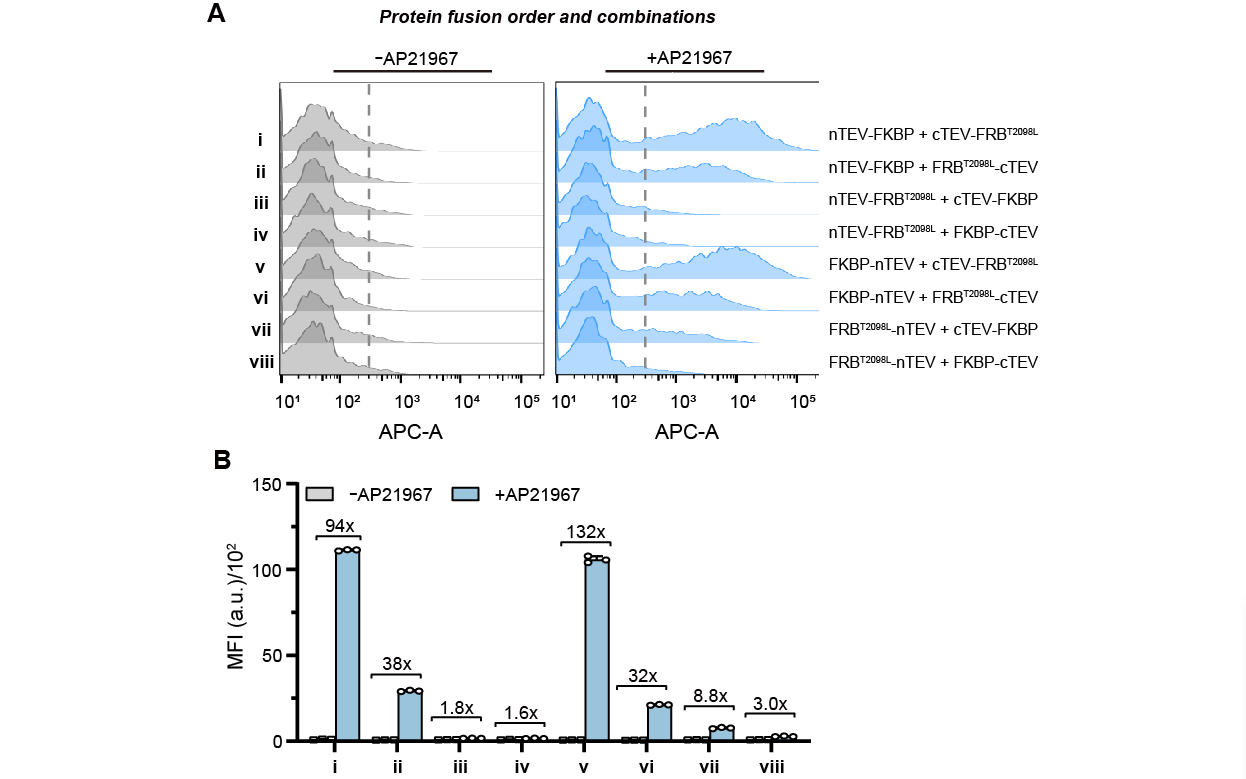


**Figure S6.** **Screening different fusion combinations of FKBP/FRB^T2098L^ domains and split TEVp fragments.** (A) Flow cytometry profiles for HeLa cells expressing the RESIT systems responsive to AP21967 with different fusion combinations. (B) MFI of iRFP670 for cells in (A). Cells expressing the RESIT system with the combination of FKBP−nTEVp and cTEVp−FRB^T2098L^ exhibited the best fold change. Error bars represented s.d. of three independent experiments.


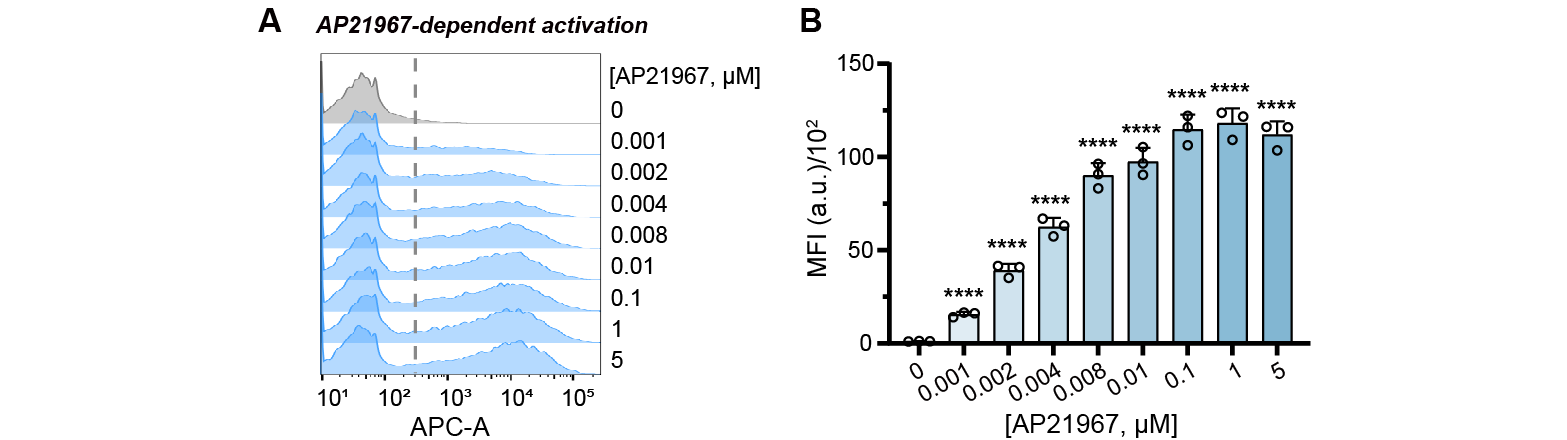


**Figure S7.** **RESIT system with dose-dependent responses to AP21967.** (A) Flow cytometry profiles for cells expressing the RESIT system treated with increasing concentrations of AP21967. (B) MFI of iRFP670 for cells in (A). The significance of differences in cells treated with different concentrations of AP21967 versus the control (0 µM) was determined using single-factor analysis of variance (ANOVA). ****p < 0.0001.


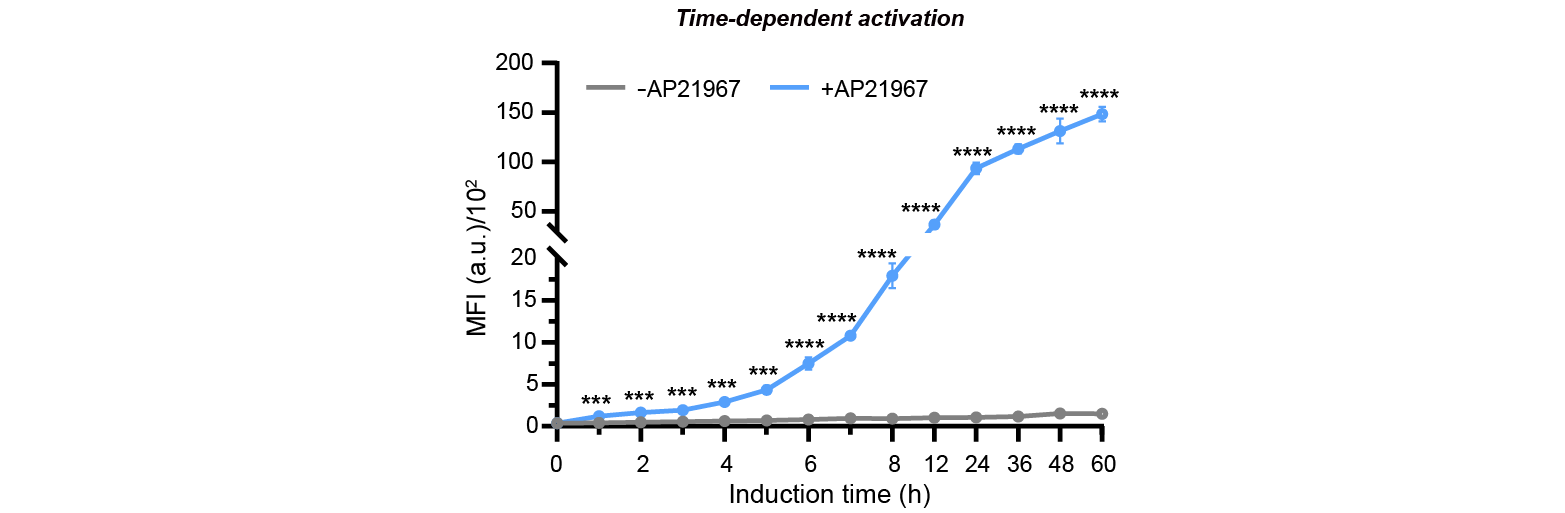


**Figure S8. Time-dependent iRFP670 expression for cells expressing AP21967-responsive RESIT system.** Time-dependent MFI of iRFP670 for cells expressing the RESIT system upon AP21967 induction. Statistical analysis was performed using a two-tailed t-test (***p = 0.0005 for AP21967 induced for 1 h. ***p = 0.0002 for AP21967 induced for 2 h, 3 h, 4 h and 5 h. ****p < 0.0001 in other time points.). Error bars represented s.d. of three independent experiments.


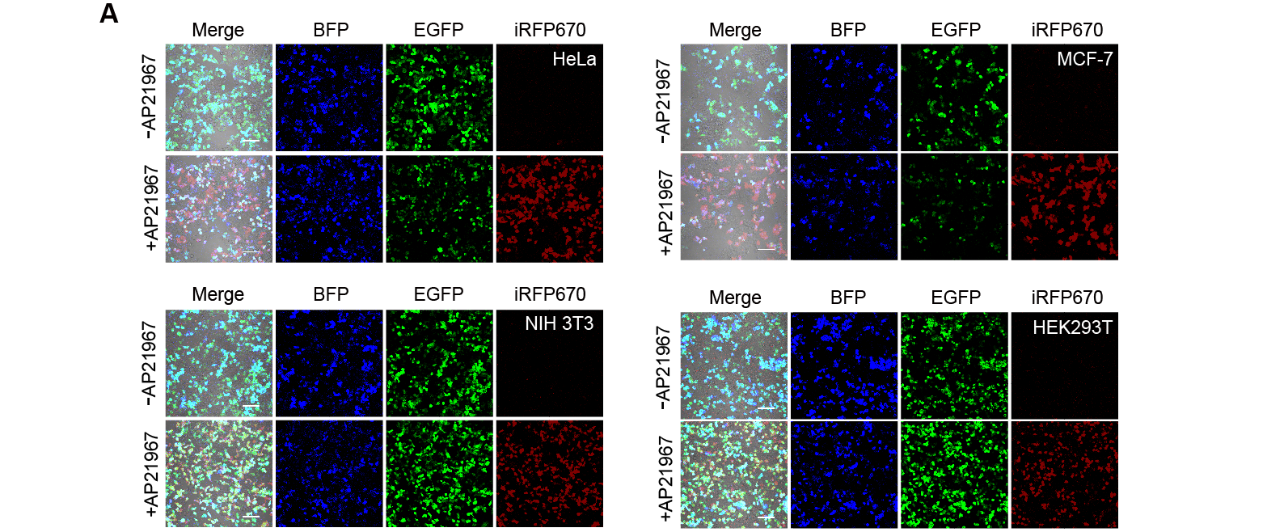

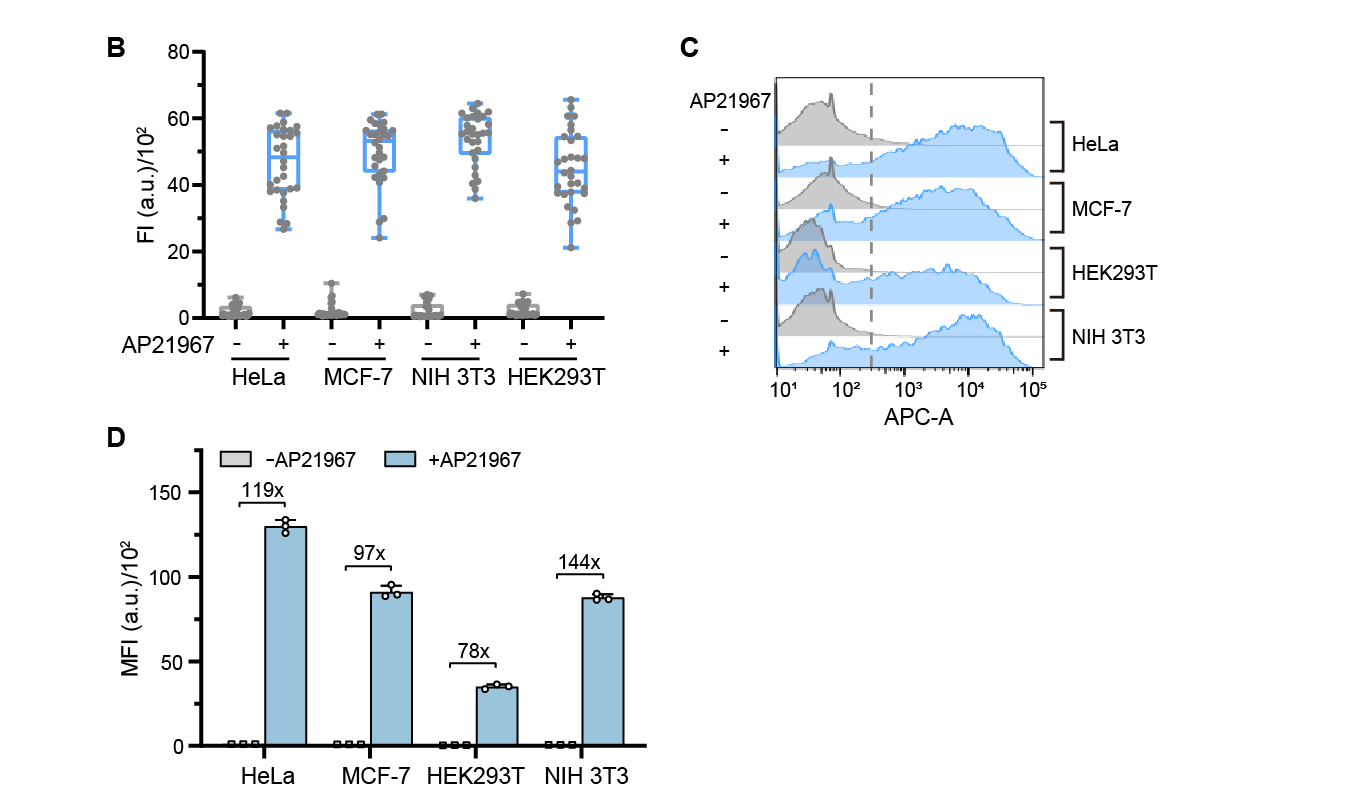


**Figure S9. The performance of AP21967-responsive RESIT system in different mammalian cell types.** (A) Representative confocal images for different cells expressing RESIT system treated with and without AP21967 (1 μM). iRFP670 fluorescence indicated the reporter. BFP fluorescence indicated the expression of tGal4(65)−cTEVp−FRB^T2098L^ module. EGFP fluorescence indicated the expression of tGal4(65)−nTEVp−FKBP module. Scale bar, 100 µm. (B) Quantitative analysis of iRFP670 fluorescence intensities for individual cells (30 cells from three independent experiments) in (A). (C) Flow cytometry profiles for different cells expressing the RESIT system with or without AP21967 induction. (D) MFI of iRFP670 as determined from flow cytometric data in (C). Error bars represented s.d. of three independent experiments.


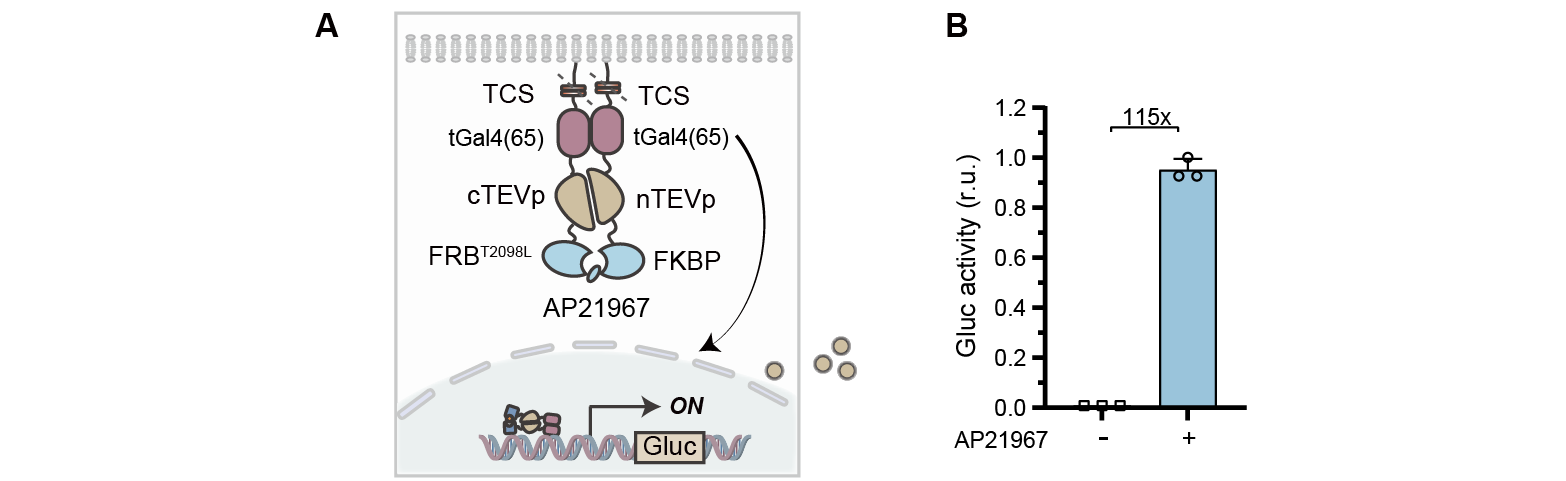


**Figure S10. Design and characterization of AP21967-responsive RESIT system using secreted Gluc as the reporter.** (A) Schematic for RESIT system responsive to AP21967 using secreted Gluc as the reporter. (B) Gluc activity for cells expressing the RESIT system with or without AP21967 (1 μM) induction. The levels of secreted Gluc in the supernatants were determined. Data were represented as mean ± s.d. of three independent measurements.


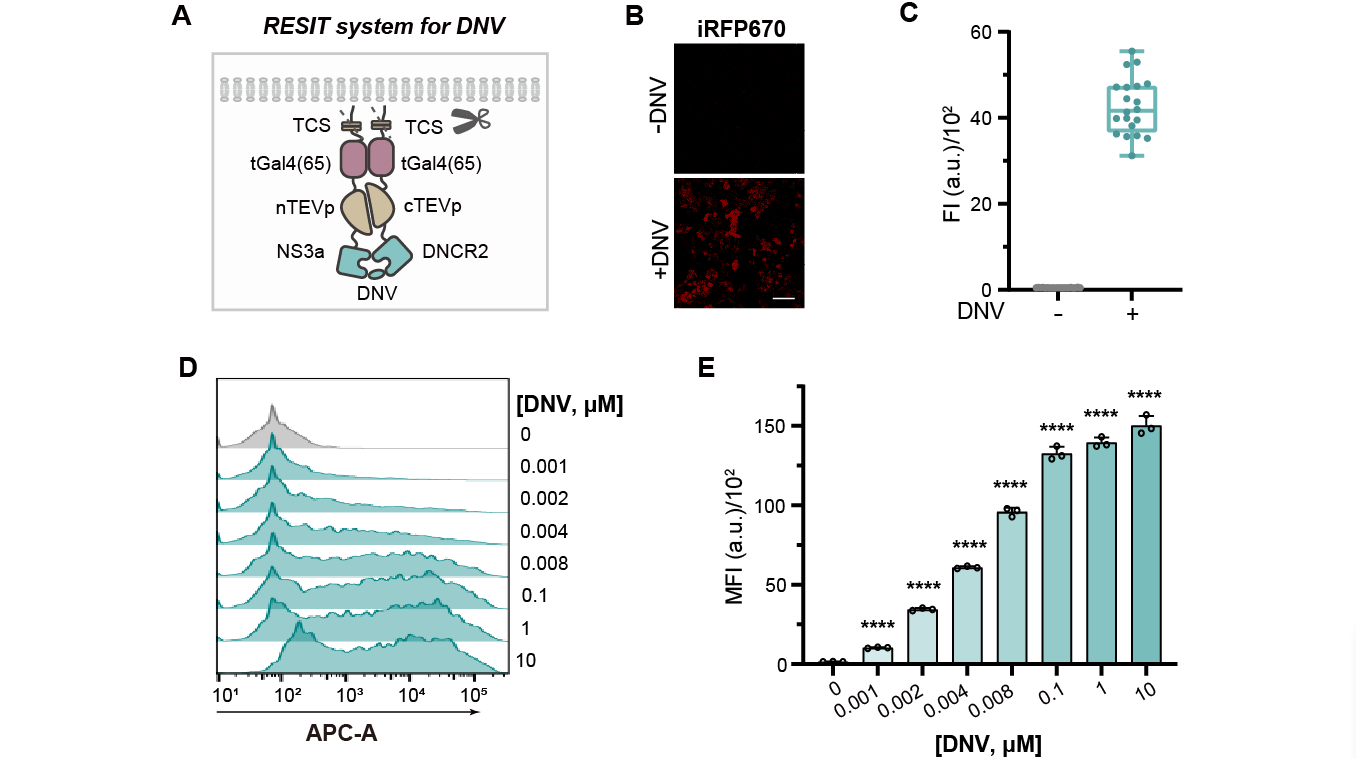


**Figure S11. Design and characterization of RESIT system responsive to danoprevir.** (A) Schematic of RESIT system responsive to danoprevir (DNV). DNV-mediated interaction between NS3a and DNCR2 results in re-assembly and release of dimerized tGal4(65) for transcription activation. (B) Representative confocal images for HeLa cells expressing RESIT system with or without DNV induction. iRFP670 fluorescence indicated the reporter. Scale bar, 100 µm. (C) Quantitative analysis of iRFP670 fluorescence intensities for individual cells (20 cells from three independent experiments) in (B). (D) Flow cytometry profiles for cells expressing the RESIT system treated with increasing concentrations of DNV. (E) MFI of iRFP670 for cells in (D). The significance of differences in cells treated with different concentrations of DNV versus the control (0 µM) was determined using single-factor analysis of variance (ANOVA). ****p < 0.0001.


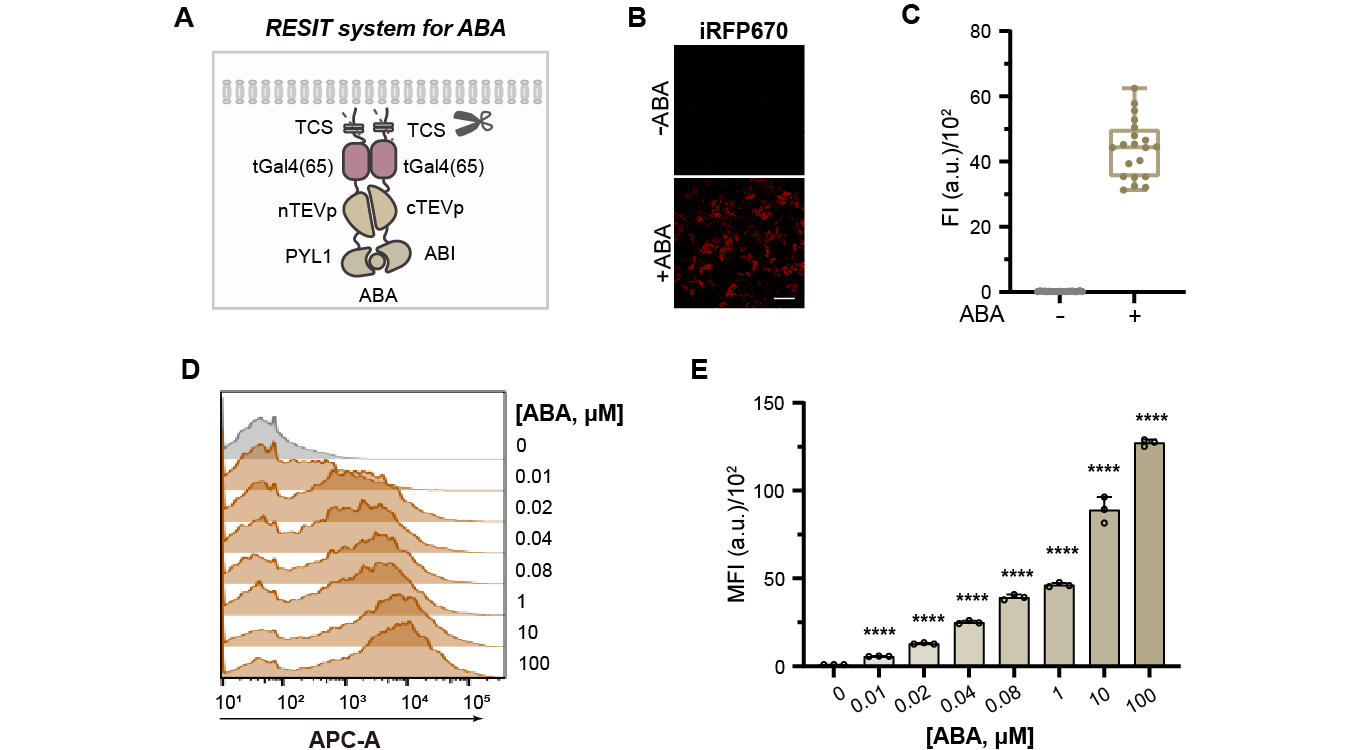


**Figure S12. Design and characterization of RESIT system responsive to abscisic acid.** (A) Schematic of RESIT system responsive to abscisic acid (ABA). ABA-mediated interaction between PYL1 and ABI results in re-assembly and release of dimerized tGal4(65) for transcription activation. (B) Representative confocal images for HeLa cells expressing RESIT system with or without ABA induction. iRFP670 fluorescence indicated the reporter. Scale bar, 100 µm. (C) Quantitative analysis of iRFP670 fluorescence intensities for individual cells (20 cells from three independent experiments) in (B). (D) Flow cytometry profiles for cells expressing the RESIT system treated with increasing concentrations of ABA. (E) MFI of iRFP670 for cells in (D). The significance of differences in cells treated with different concentrations of ABA versus the control (0 µM) was determined using single-factor analysis of variance (ANOVA). ****p < 0.0001.


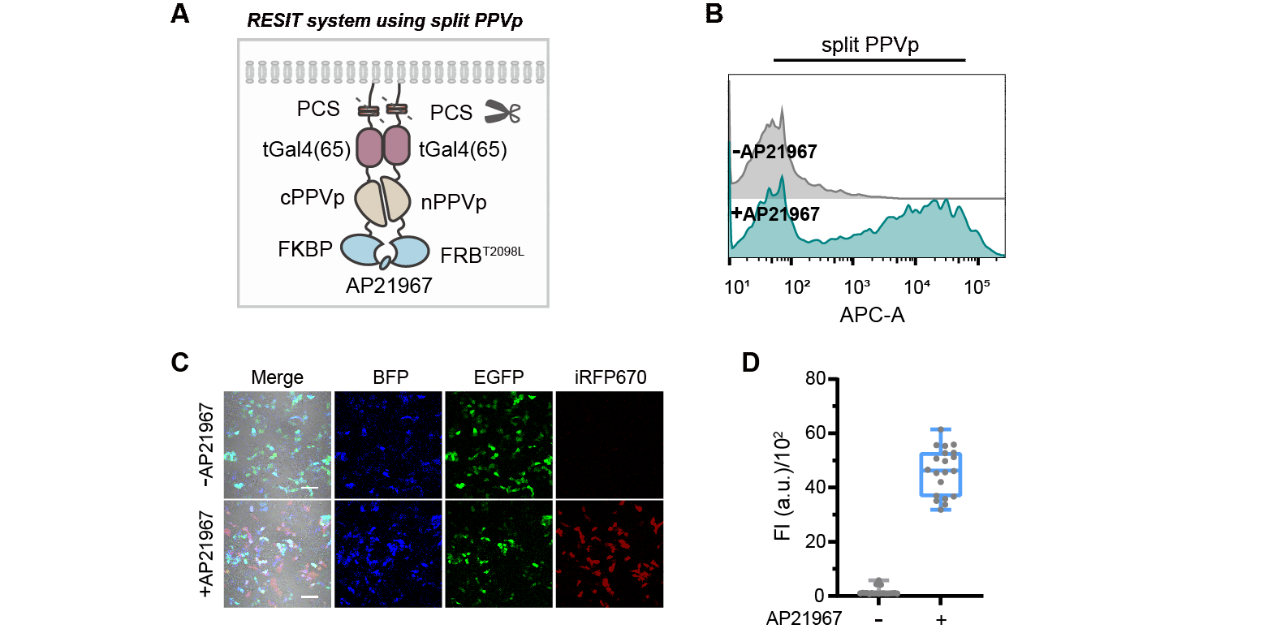


**Figure S13. Design and characterization of RESIT system using split PPVp as the protease.** (A) Schematic of the RESIT system using split PPVp as the protease. (B) Flow cytometry profiles of cells expressing the RESIT system using split PPVp as the protease with or without AP21967 (1 μM) induction. (C) Representative confocal images for cells expressing the RESIT system using split PPVp as the protease with or without AP21967 (1 μM). iRFP670 fluorescence indicated the reporter. BFP fluorescence indicated the expression of tGal4(65)−nPPVp−FRB^T2098L^ module. EGFP fluorescence indicated the expression of tGal4(65)−cPPVp−FKBP module. The iRFP670 fluorescence remarkably increased upon AP21967 induction. Scale bar, 100 µm. (D) Quantitative analysis of iRFP670 fluorescence intensities for individual cells (20 cells from three independent experiments) in (C).


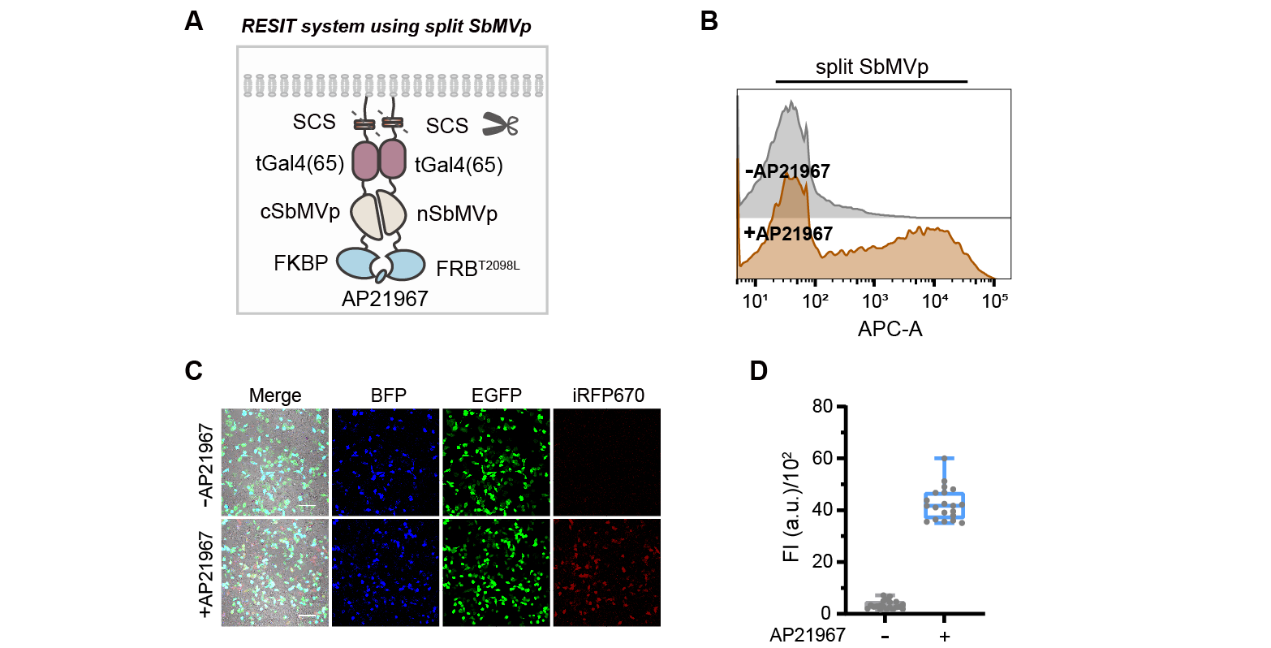


**Figure S14. Design and characterization of RESIT system using split SbMVp as the protease.** (A) Schematic of the RESIT system using split SbMVp as the protease. (B) Flow cytometry profiles of cells expressing the RESIT system using split SbMVp as the protease with or without AP21967 (1 μM) induction. (C) Representative confocal images for cells expressing the RESIT system using split SbMVp as the protease with or without AP21967 (1 μM). iRFP670 fluorescence indicated the reporter. BFP fluorescence indicated the expression of tGal4(65)−nSbMVp−FRB^T2098L^ module. EGFP fluorescence indicated the expression of tGal4(65)−cSbMVp−FKBP module. The iRFP670 fluorescence remarkably increased upon AP21967 induction. Scale bar, 100 µm. (D) Quantitative analysis of iRFP670 fluorescence intensities for individual cells (20 cells from three independent experiments) in (C).


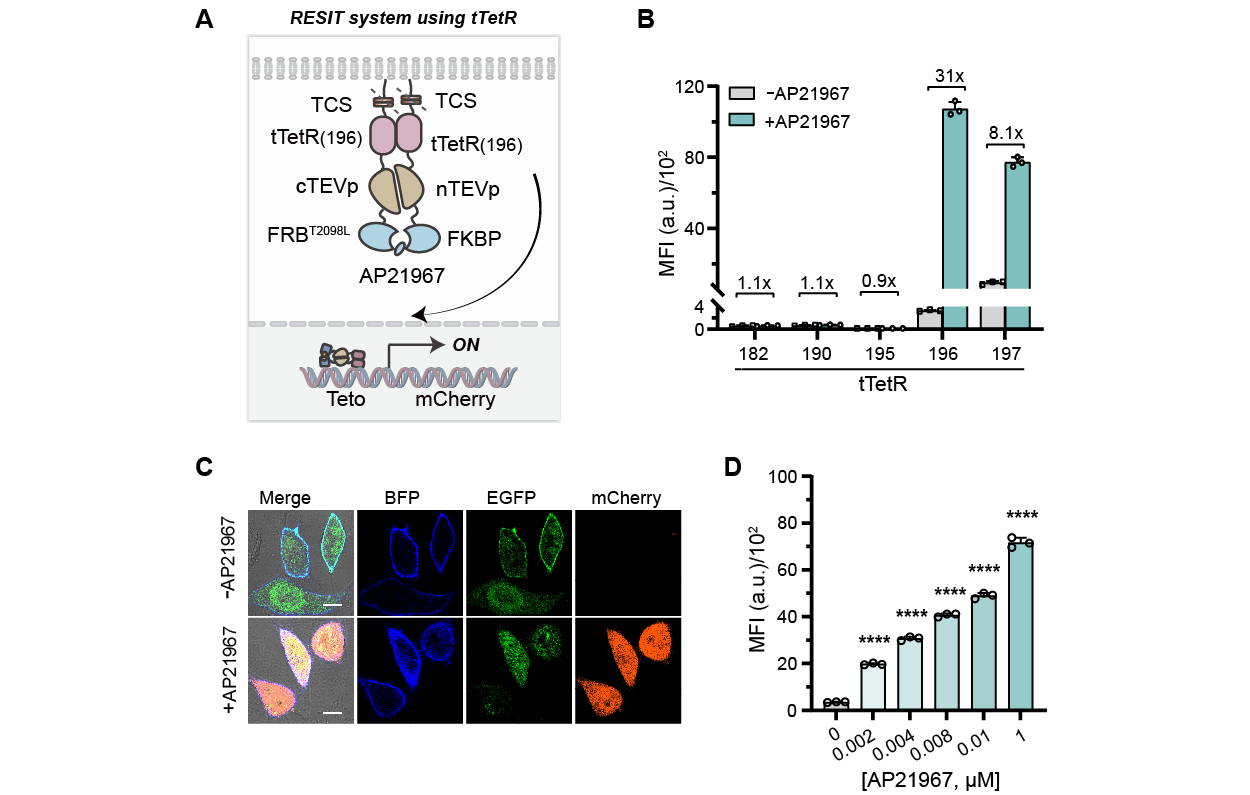


**Figure S15.** **Design and characterization of RESIT system using truncated DBD of TetR.** (A) Schematic of the RESIT system using the DBD of truncated TetR (tTetR). AP21967 mediated partnering between FKBP and FRB^T2098L^ results in re-assembly and release of tTetR(196) for transcription activation of mCherry. (B) Optimization of RESIT using different tTetR variants under AP21967 (1 µM) induction determined by MFI of mCherry from flow cytometry profiles. (C) Representative confocal images for cells expressing the RESIT system using tTetR(196) as the DBD with or without AP21967 (1 μM) induction. iRFP670 fluorescence indicated the reporter. BFP fluorescence indicated the expression of tTetR(196)−cTEVp−FRB^T2098L^ module. EGFP fluorescence indicated the expression of tTetR(196)−nTEVp−FKBP module. Scale bar, 10 µm. (D) MFI of mCherry for cells expressing the RESIT system using tTetR(196) as the DBD treated with different concentrations of AP21967. The significance of differences in cells treated with different concentrations of AP21967 versus the control (0 µM) was determined using single-factor ANOVA. ****p < 0.0001.


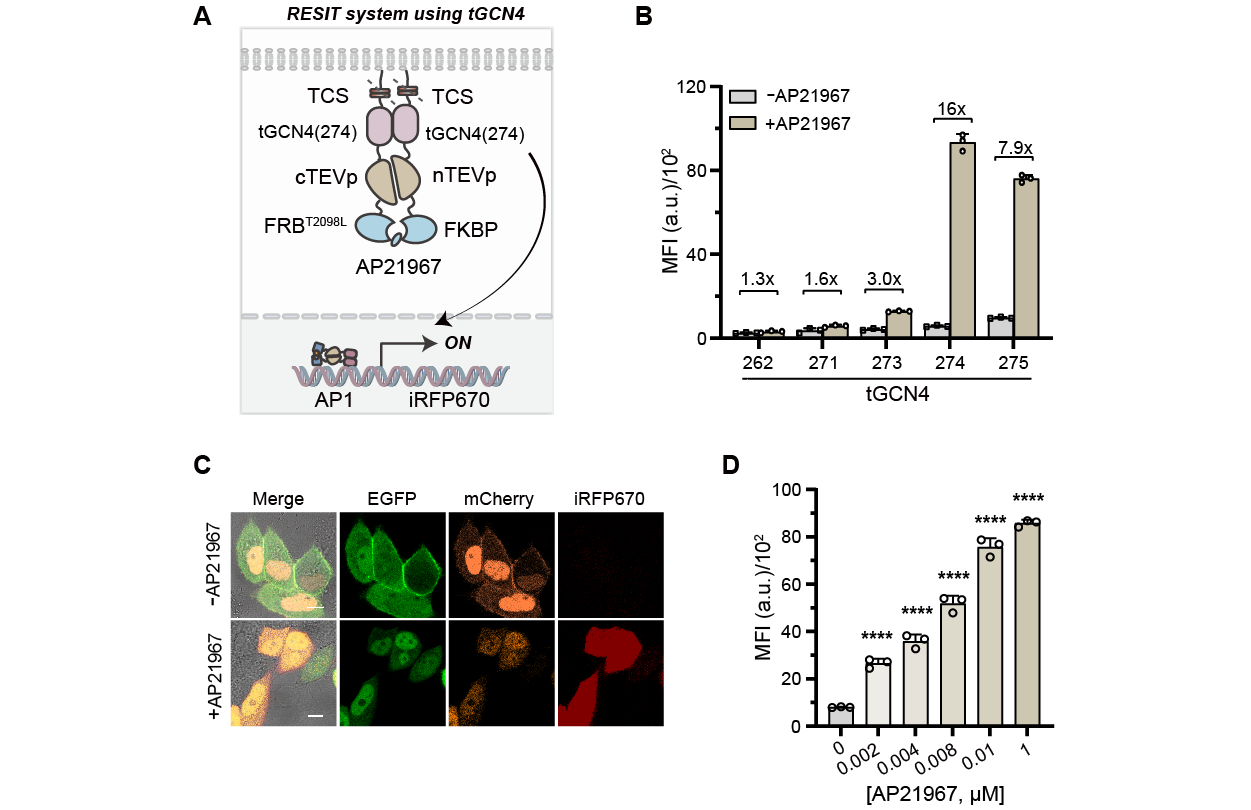


**Figure S16. Design and characterization of RESIT system using truncated DBD of GCN4.** (A) Schematic of the RESIT system using the DBD of truncated GCN4 (tGCN4). AP21967 mediated partnering between FKBP and FRB^T2098L^ results in re-assembly and release of tGCN4(274) for transcription activation of iRFP670. (B) Optimization of the RESIT using different tGCN4 variants under AP21967 (1 μM) induction determined by MFI of iRFP670 from flow cytometry profiles. (C) Representative confocal images for cells expressing the RESIT system using tGCN4(274) as the DBD with or without AP21967 (1 μM) induction. iRFP670 fluorescence indicated the reporter. EGFP fluorescence indicated the expression of tGCN4(274)−cTEVp−FRB^T2098L^ module. mCherry fluorescence indicated the expression of tGCN4(274)−nTEVp−FKBP module. Scale bar, 10 µm. (D) MFI of iRFP670 for cells expressing the RESIT system using tGCN4(274) as the DBD treated with different concentrations of AP21967. The significance of differences in cells treated with different concentrations of AP21967 versus the control (0 µM) was determined using single-factor ANOVA. ****p < 0.0001.


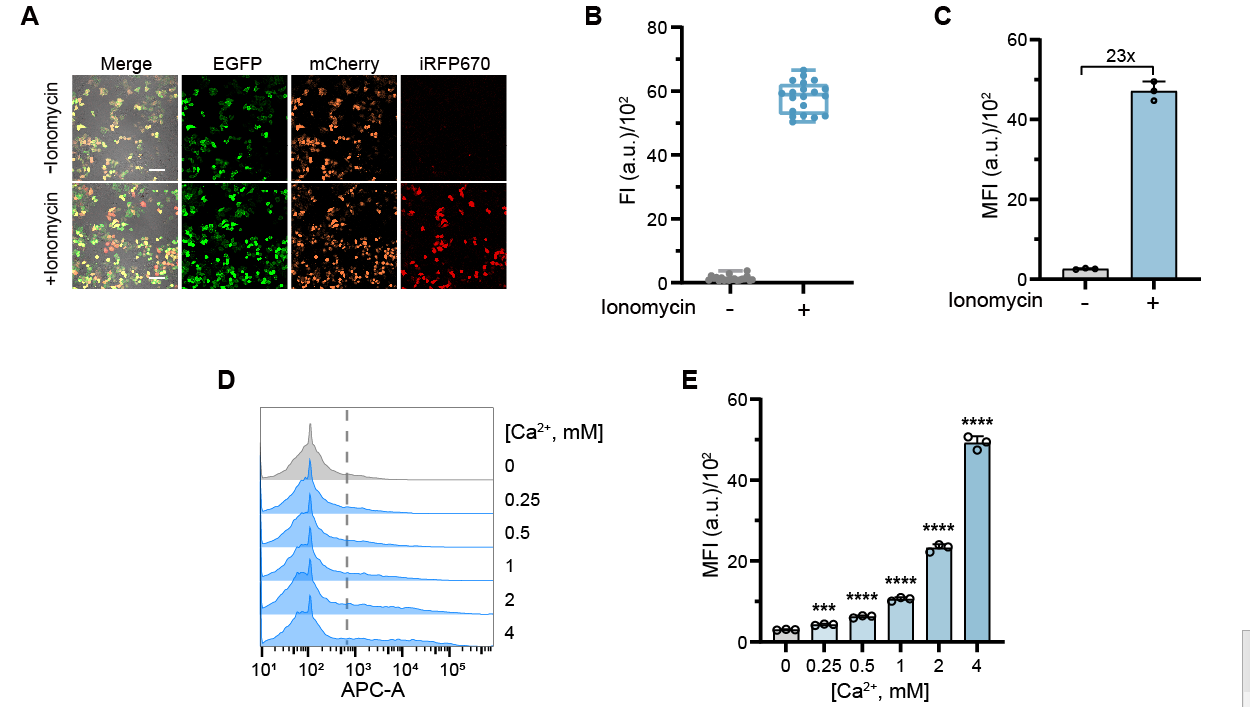


**Figure S17. Characterization of Ca^2+^-responsive RESIT system upon ionomycin induction.** (A) Representative confocal images for HeLa cells expressing the RESIT system responsive to Ca^2+^ treated with or without Ca^2+^ (4 mM) and ionomycin (2 μM). iRFP670 indicated the reporter. EGFP fluorescence indicated the expression of tGal4(65)−nTEVp−CaM module, while the mCherry fluorescence indicated the expression of tGal4(65)−cTEVp−M13 module. Scale bar, 100 µm. (B) Quantitative analysis of iRFP670 fluorescence intensities for individual cells (20 cells from three independent experiments) in (A). (C) MFI of iRFP670 for cells in (A). Error bars represented s.d. of three independent experiments. (D) Flow cytometry profiles for HeLa cells expressing the Ca^2+^-responsive RESIT system treated with increasing concentrations of Ca^2+^ in the presence of ionomycin. (E) MFI of iRFP670 for cells in (D). The significance of differences for different concentrations versus without Ca^2+^ was determined using single-factor ANOVA (***p = 0.006, ****p < 0.0001).


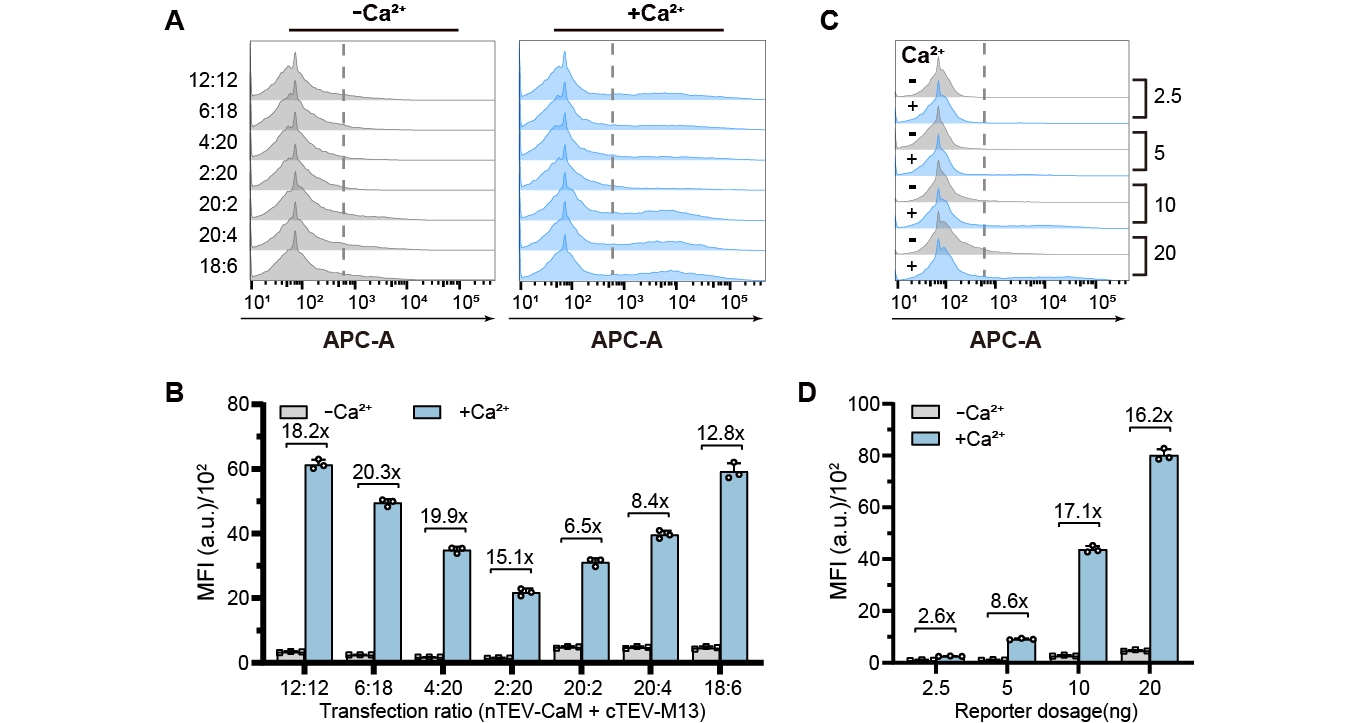


**Figure S18. Optimization of the ratios of plasmid amounts for Ca^2+^-responsive RESIT system.** (A) Flow cytometry profiles for HeLa cells transfected with the membrane-tethered transcriptional modules at different ratios with a fixed reporter amount with or without Ca^2+^ (4 mM) and ionomycin (2 μM) treatment. (B) MFI of iRFP670 for cells in (A). (C) Flow cytometry profiles for HeLa cells transfected with varying amounts of reporter plasmid while keeping the membrane-tethered transcriptional modules at a ratio of 1:3 with or without Ca^2+^ (4 mM) and ionomycin (2 μM) treatment. (D) MFI of iRFP670 for cells in (C). (B, D) Statistical analysis was performed using a two-tailed t-test (Left to right: ****p < 0.0001). Data were represented as mean ± s.d. of three independent measurements.


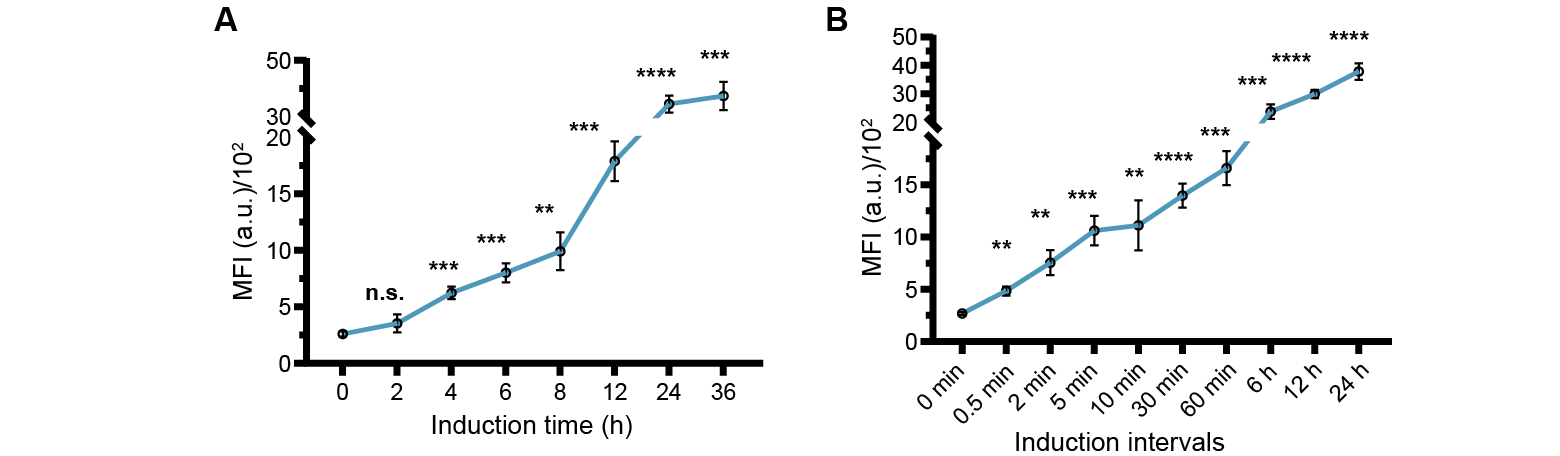


**Figure S19. Characterization the response time and effective detection window for Ca^2+^-responsive RESIT system.** (A) MFI of iRFP670 for HeLa cells expressing Ca^2+^-responsive RESIT system upon induction with Ca^2+^ (4 mM) and ionomycin (2 μM) for different periods as determined by flow cytometry. HeLa cells were transfected with the Ca²⁺-responsive RESIT system for 24 h, stimulated with Ca²⁺ for various time periods, and then subjected to flow cytometry analysis. Statistical analysis was performed using a two-tailed t-test (Left to right: p = 0.1200, ***p = 0.0004, ***p = 0.0004, **p = 0.0017, ***p = 0.0001, ****p < 0.0001, ***p = 0.0003). (B) MFI of iRFP670 for HeLa cells expressing Ca^2+^-responsive RESIT system at different time intervals upon induction with Ca^2+^ (4 mM) and ionomycin (2 μM) as determined by flow cytometry. HeLa cells were transfected with the Ca²⁺-responsive RESIT system for 24 h, stimulated with Ca²⁺ for various time periods, incubated for another 24 h prior to flow cytometry. Statistical analysis was performed using a two-tailed t-test (Left to right: **p = 0.0011, **p = 0.0023, ***p = 0.0006, **p = 0.0036, ****p < 0.0001, ***p = 0.0001, ***p = 0.0001, ****p < 0.0001).


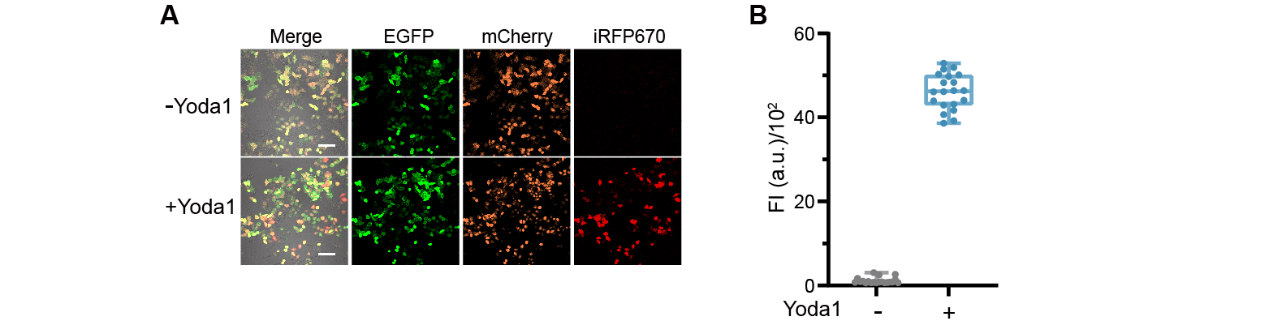


**Figure S20. Confocal images for cells expressing Ca^2+^-responsive RESIT system.** (A) Representative confocal images for HeLa cells expressing the RESIT system responsive to Ca^2+^ with or without Yoda1 (10 μM) induction. iRFP670 indicated the reporter. EGFP fluorescence indicated the expression of tGal4(65)−nTEVp−CaM module, and mCherry fluorescence indicated the expression of tGal4(65)−cTEVp−M13 module. Scale bar, 100 µm. (B) Quantitative analysis of iRFP670 fluorescence intensities for individual cells (20 cells from three independent experiments) in (A).


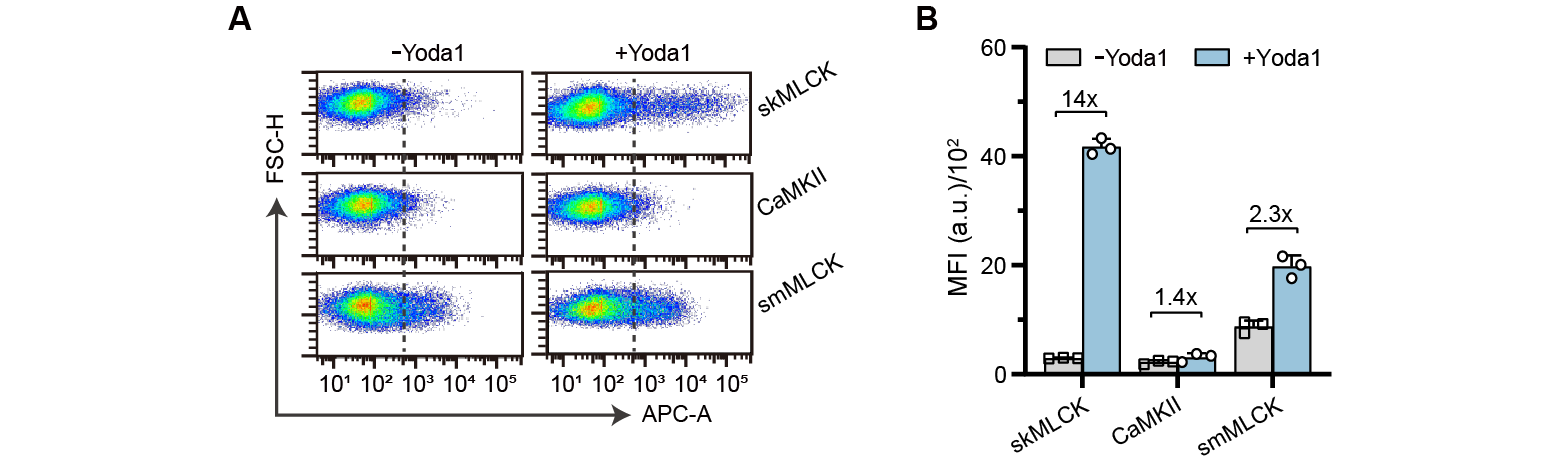


**Figure S21. RESIT optimization using different M13 variants.** (A) Optimization of the RESIT using different M13 variants. Flow cytometry profile for HeLa cells expressing the RESIT with different M13 variants with or without Yoda1 (10 μM) induction. (B) MFI of iRFP670 for cells in (A).


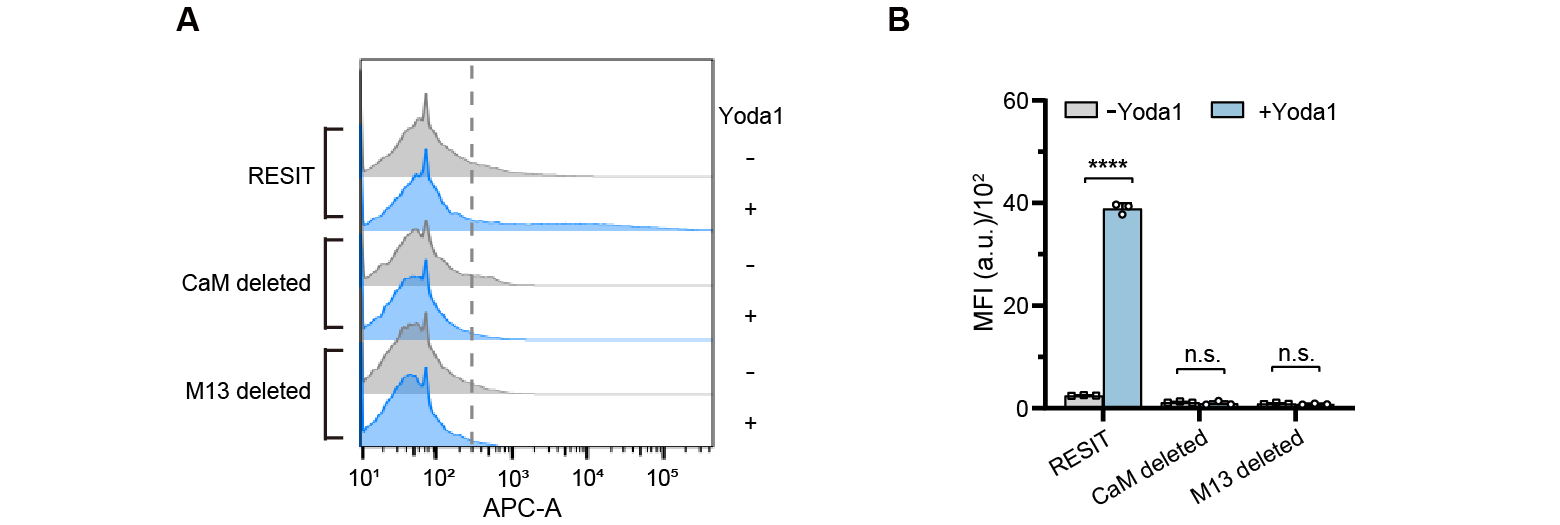


**Figure S22. Specificity of RESIT design responsive to Ca^2+^ influx.** (A) Flow cytometry profiles for cells expressing the Ca^2+^-responsive RESIT system with CaM or M13 deleted with or without Yoda1 induction. (B) MFI of iRFP670 fluorescence for cells in (A). Negligible iRFP670 fluorescence was obtained for cells expressing the RESIT system with CaM or M13 deleted after Yoda1 induction. Statistical analysis was performed using a two-tailed t-test (****p < 0.0001, n.s., p = 0.5313 for the system with CaM deleted and p = 0.2731 for the system with M13 deleted). Error bars represented s.d. of three independent experiments.


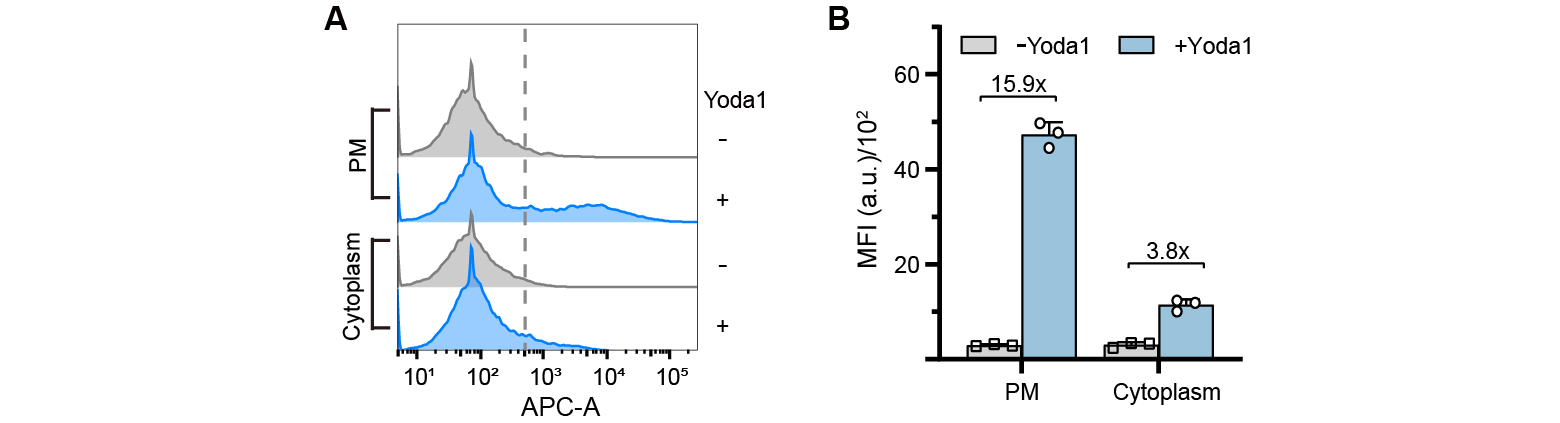


**Figure S23. Comparison between membrane-tethered RESIT (PM) or the cytosol-localized counterpart (Cytoplasm) in sensing Ca^2+^ influx upon Yoda1 induction.** (A) Flow cytometry profiles for comparison of membrane-tethered RESIT (PM) and cytosol-localized RESIT (Cytoplasm) in sensing Ca^2+^ in HeLa cells under Yoda1 (10 μM) induction. (B) MFI of iRFP670 for cells in (A).


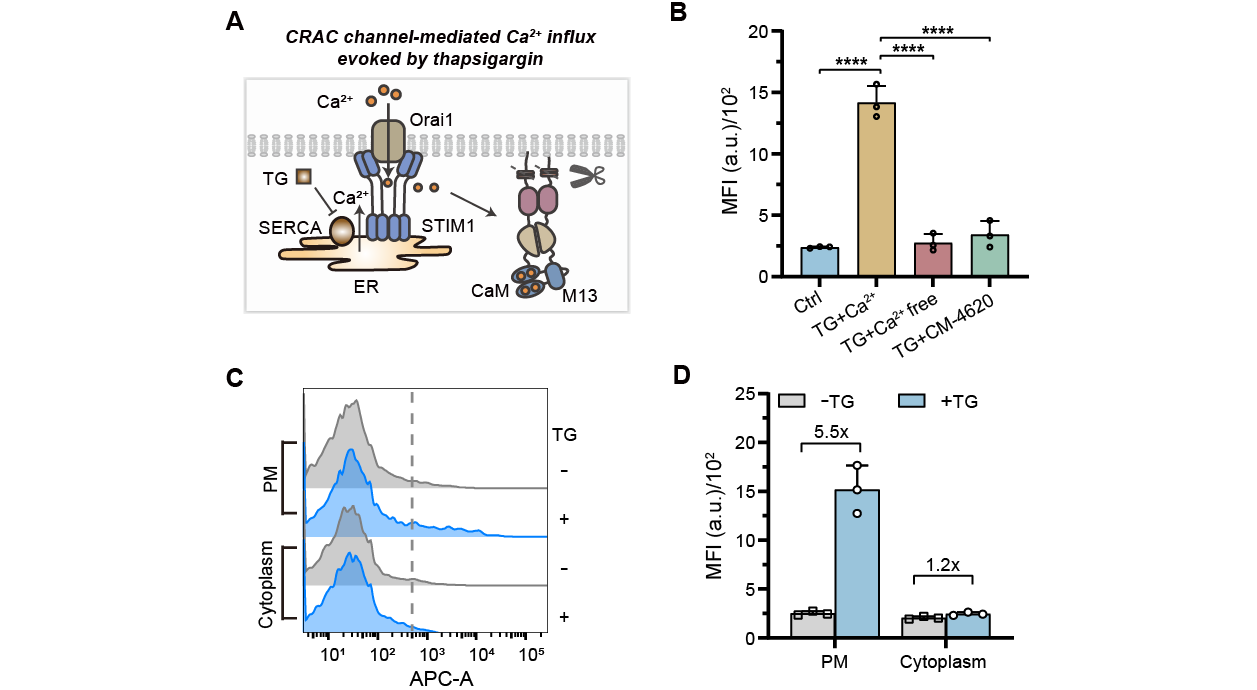


**Figure S24. Design and characterization of RESIT system responsive to Ca^2+^ influx upon TG induction.** (A) Schematic of RESIT system for sensing Ca^2+^ entry across the Ca^2+^ channel triggered by thapsigargin (TG) mediated Ca^2+^ release from endoplasmic reticulum (ER). TG is a sarco/endoplasmic reticulum Ca^2+^-ATPase (SERCA) pump inhibitor that passively depletes Ca^2+^ in the ER store. Upon Ca²⁺ release from the ER, STIM1 oligomerizes and subsequently moves to the ER-PM junctions, binds and activates Orai1 channels for Ca²⁺ entry. (B) MFI of iRFP670 for HeLa cells expressing the RESIT system treated with TG (100 nM), TG and Ca^2+^ free medium or TG and the channel inhibitor CM4620 (1 μM). The significance of differences in cells with different treatments versus those treated with TG was determined using single-factor ANOVA (****p < 0.0001). (C) Flow cytometry profiles for HeLa cells expressing the membrane-tethered Ca^2+^-responsive RESIT system (PM) or the cytosol-localized counterpart (Cytoplasm) with or without TG (100 nM) induction. (D) MFI of iRFP670 for cells in (C).


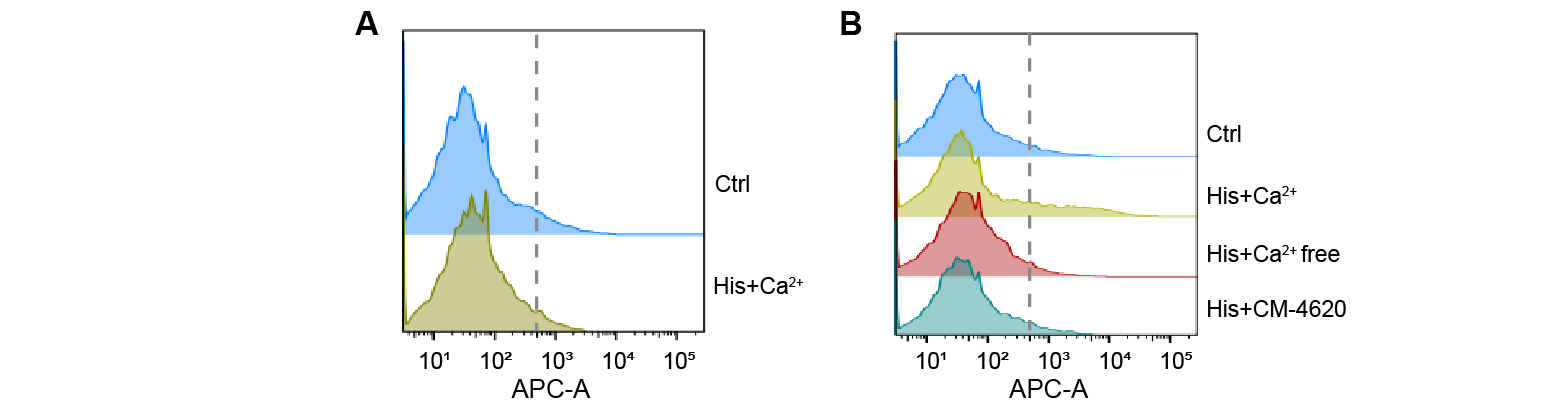


**Figure S25.** **Design and characterization of RESIT system responsive to Ca^2+^ influx upon H1R activation.** (A) Flow cytometry profiles for MCF-7 cells expressing the RESIT system treated with or without histamine (His, 100 μM). (B) Flow cytometry profiles for MCF-7 cells co-expressing H1R and the RESIT system treated with histamine (100 μM), histamine in Ca^2+^ free medium or histamine and the channel inhibitor CM4620 (1 μM).


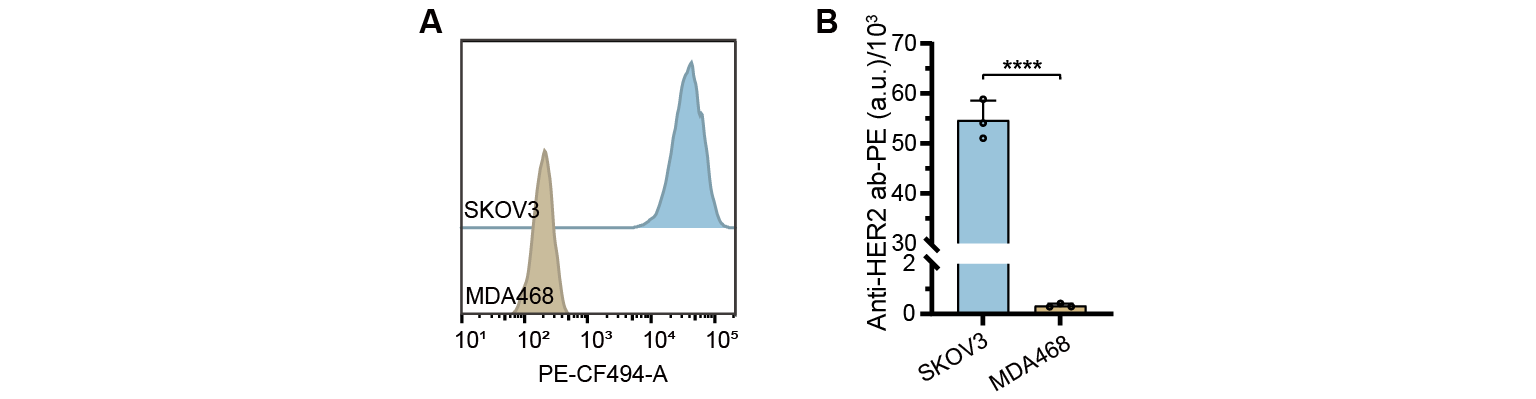


**Figure S26. Detection of HER2 densities on different cell lines.** (A) Flow cytometry profiles for different cell lines stained by PE-labeled anti-HER2 antibody. (B) MFI of mCherry for cells in (A). Statistical analysis was performed using a two-tailed t-test (Left to right: ****p < 0.0001). Data were represented as mean ± s.d. of three independent measurements.


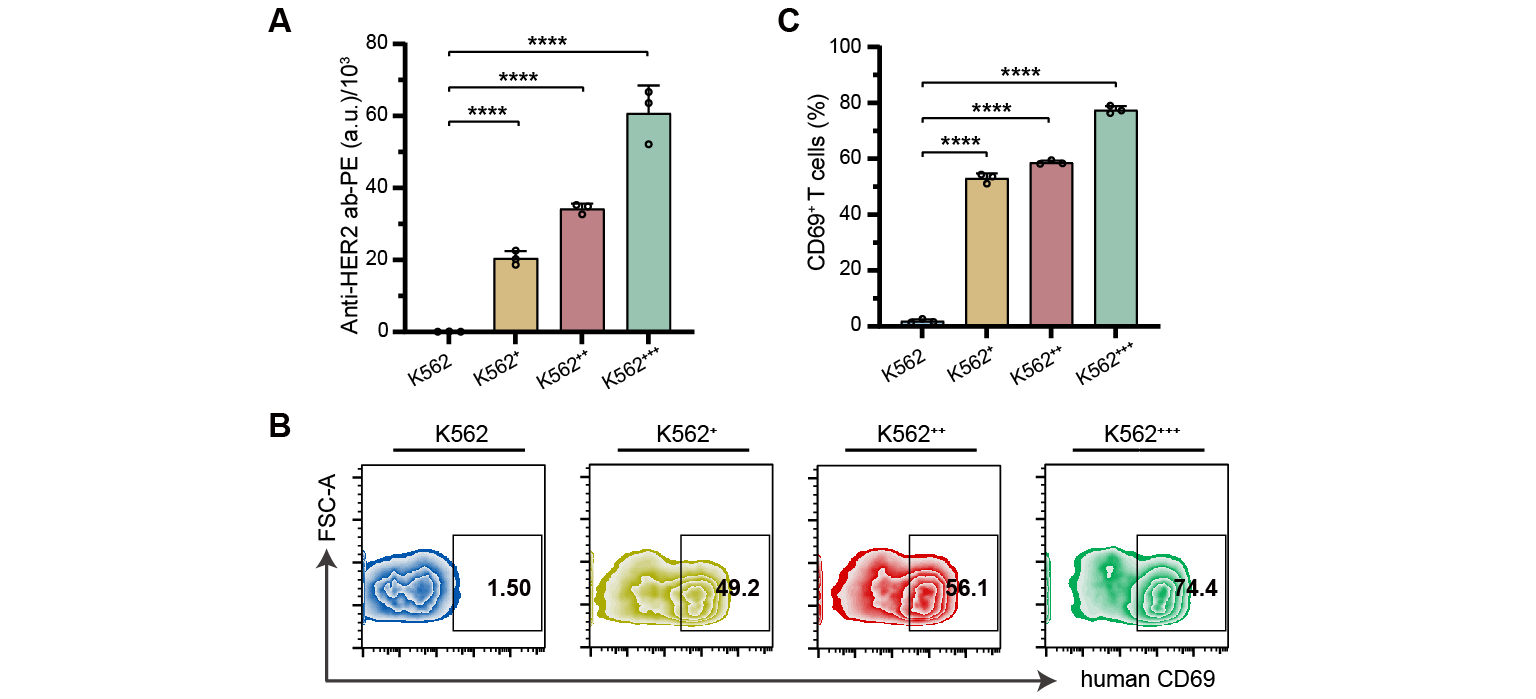


**Figure S27. The RESIT system for sensing Ca^2+^ influx in T cells co-cultured with K562 cells expressing different HER2 densities.** (A) MFI of mCherry from flow cytometry profiles for K562 cells with different HER2 densities stained by PE-labeled anti-HER2 antibody. Statistical analysis was performed using a two-tailed t-test (****p < 0.0001). (B) Flow cytometry profiles for Jurkat T cells transduced with RESIT system co-cultured with K562 cells with different HER2 densities stained by APC-labeled anti-CD69 antibody. (C) Percentages of CD69^+^ T cell in cells under different treatments in (B). Statistical analysis was performed using a two-tailed t-test (****p < 0.0001).


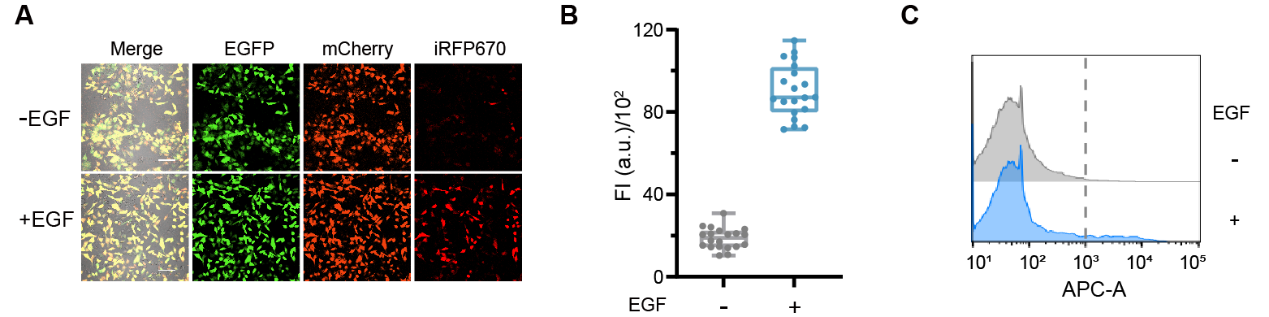


**Figure S28. Responses of cells expressing RTK-responsive RESIT system upon EGF induction.** (A) Representative confocal images for cells expressing the RTK-responsive RESIT system treated with or without EGF (100 ng/ml). iRFP670 fluorescence indicated the reporter. EGFP fluorescence indicated the expression of VAV1−tGal4(65)−nTEVp−NPXY module. mCherry indicated the expression of tGal4(65)−cTEVp−PTB module. Scale bar, 100 µm. (B) Quantitative analysis of iRFP670 fluorescence intensities for individual cells (20 cells from three independent experiments) in (A). (C) Flow cytometry profiles for cells in (A).


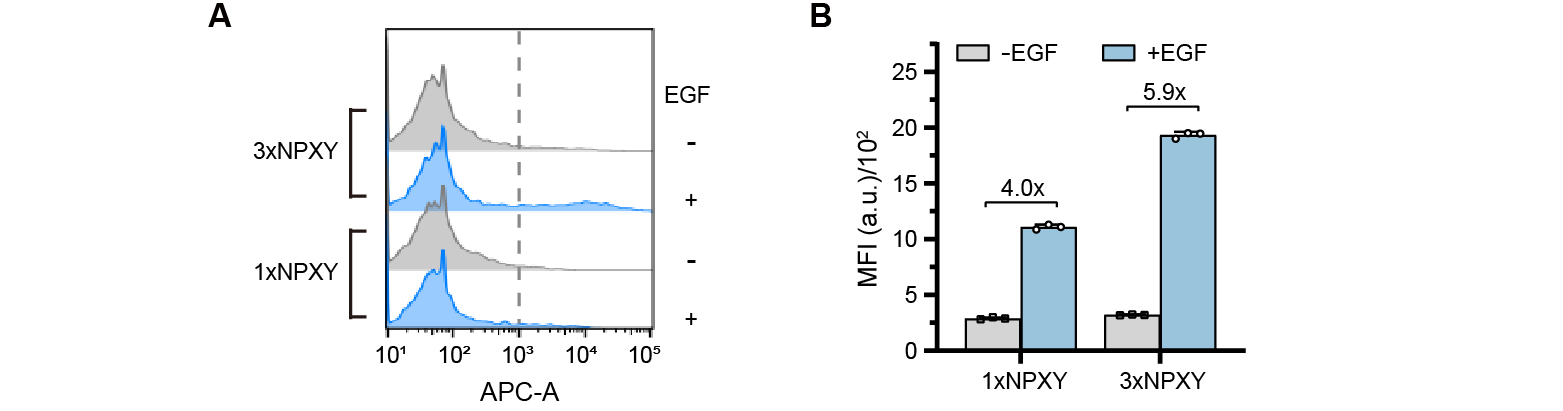


**Figure S29. Comparison of RTK-responsive RESIT with one substrate motif and three tandem substrate motifs.** (A) Flow cytometry profiles for cells expressing the RTK-responsive RESIT system with one substrate motif (1×NPXY) and three tandem substrate motifs (3×NPXY) in the sensing modules with or without EGF (100 ng/ml) induction. (B) MFI of iRFP670 for cells in (A). Data were represented as mean ± s.d. of three independent measurements.


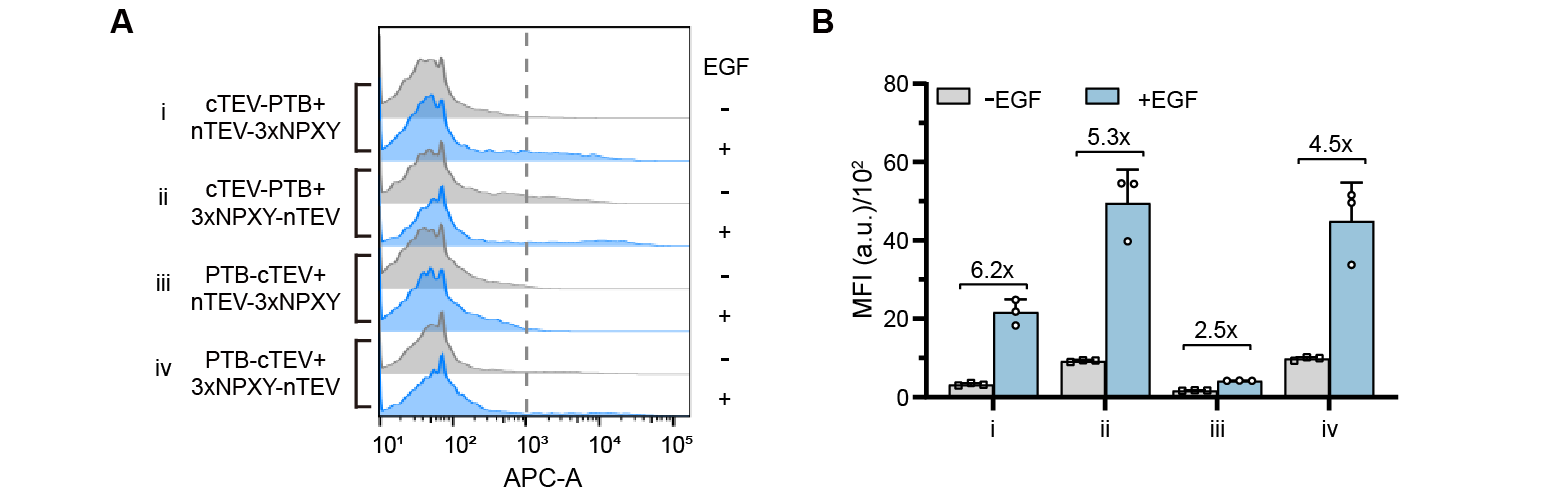


**Figure S30. Screening different fusion combinations of PTB/3xNPXY domains and split TEVp fragments.** (A) Flow cytometry profiles for HeLa cells expressing the RTK-responsive RESIT system with different fusion combinations with or without EGF induction. (B) MFI of iRFP670 for cells in (A). Cells expressing the RESIT system with the combination of VAV1−nTEVp−3×NPXY and cTEVp−PTB exhibited the best fold change. Error bars represented s.d. of three independent experiments.


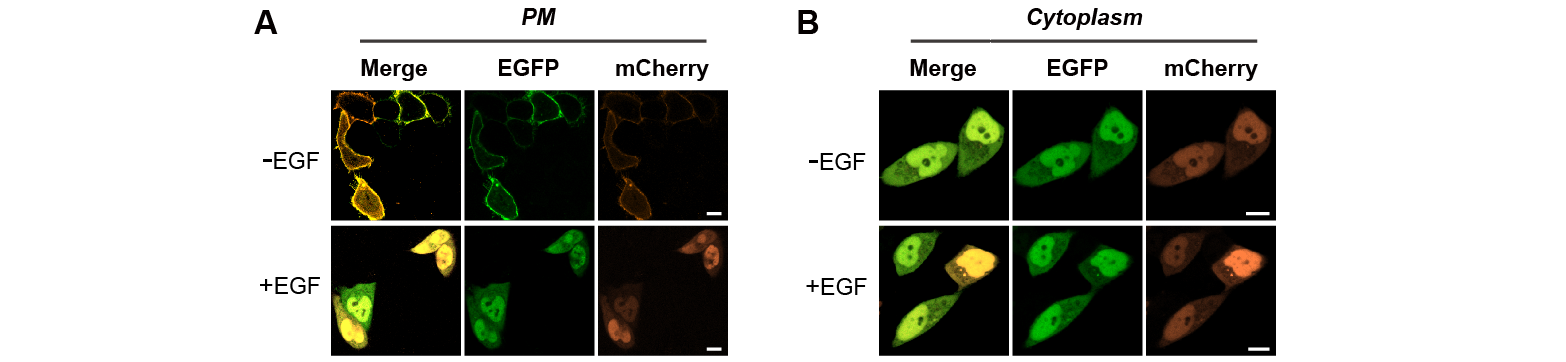
**Figure S31. Comparison of localization differences between membrane-tethered and cytosol-localized RTK-responsive RESIT upon induction.** (A) Confocal images for cells expressing the membrane-tethered RTK-responsive RESIT before and after induction with EGF for 24 h. The transcriptional modules were predominantly localized to the plasma membrane before EGF induction. Appreciable release and translocation of the transcriptional modules to the cytosol and nuclei was observed after EGF induction. Scale bar, 10 µm. (B) Confocal images for cells expressing the cytosol-localized RTK-responsive RESIT before and after treatment with EGF for 24 h. No appreciable localization of the transcriptional modules to the plasma membrane was observed before and after EGF induction. Scale bar, 10 µm.


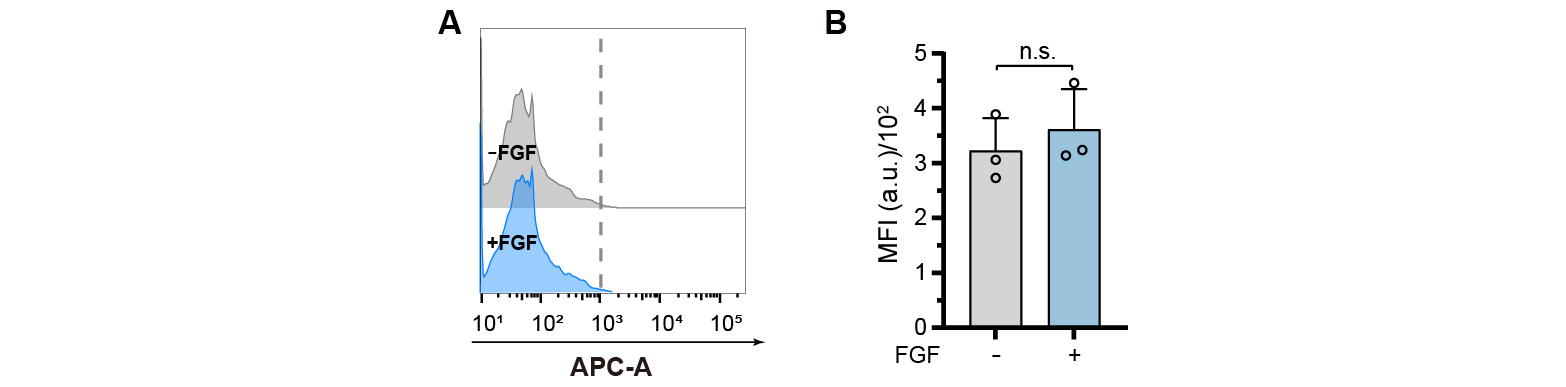


**Figure S32. Response of RTK-responsive RESIT upon FGFR activation.** (A) Flow cytometry profiles for RTK-responsive RESIT expressing HEK293T cells with exogenous transfection of FGFR upon induction with FGF for 24 h. (B) MFI of iRFP670 for cells in (A). Statistical analysis was performed using a two-tailed t-test (p = 0.5186). Error bars represented s.d. of three independent experiments.


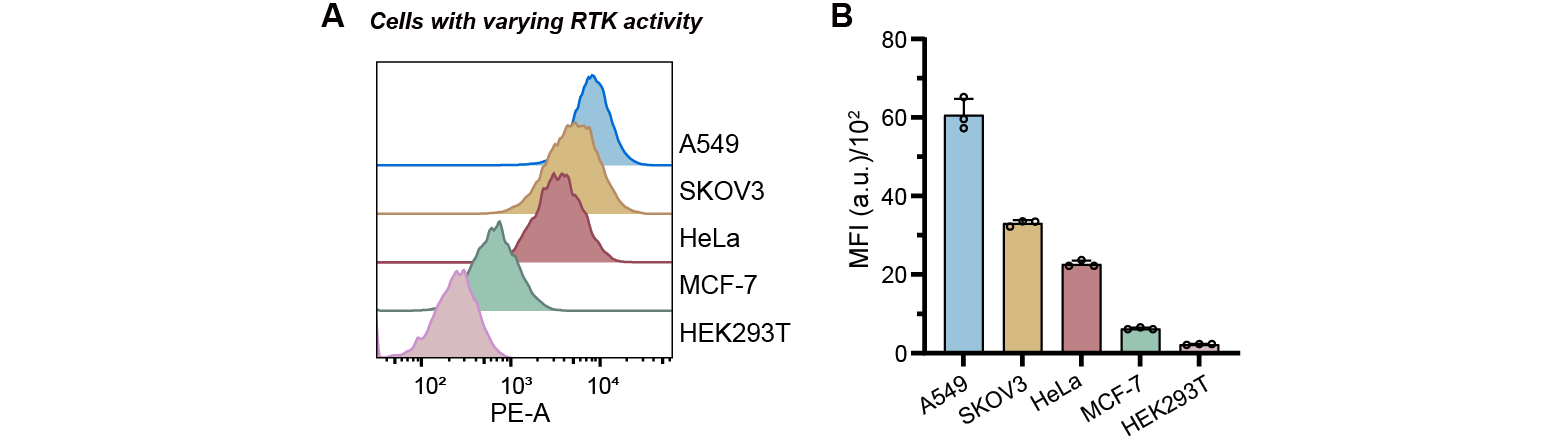


**Figure S33.** **Detection of EGFR densities on different cell lines.** (A) Flow cytometry profiles for different cell lines stained by PE-labeled anti-EGFR antibody. (B) MFI of mCherry for cells in (A). A549 cells showed the highest level of EGFR on the surface, while HEK293T cells had very low expression of surface EGFR.


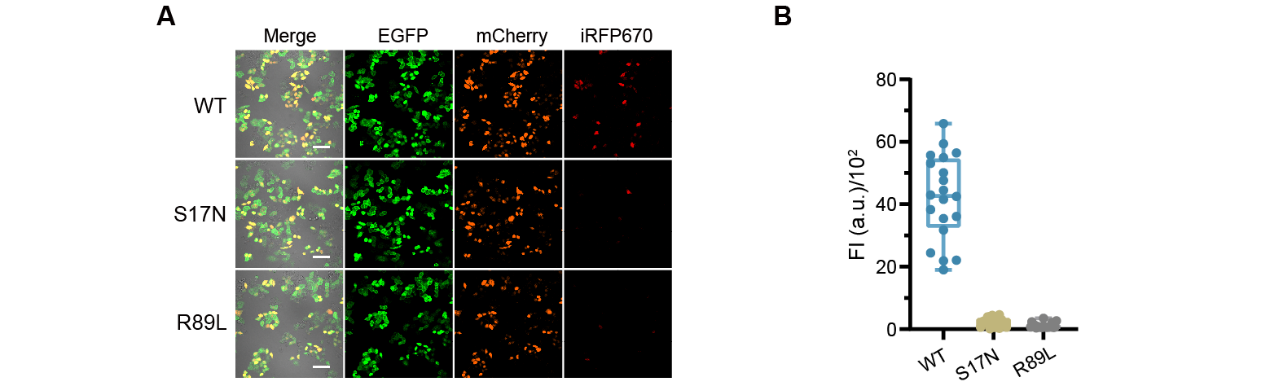


**Figure S34. Specificity of RESIT design for sensing RAS activities.** (A) Representative confocal images for HeLa cells expressing RAS-responsive RESIT system with wide type (WT) Ras, Ras mutant (S17N) or Raf mutant (R89L) in the sensing modules. iRFP670 fluorescence indicated the reporter. EGFP fluorescence indicated the expression of tGal4(65)−nTEVp−RBD. mCherry indicated the expression of tGal4(65)−cTEVp−KRas. Scale bar, 100 µm. (B) Quantitative analysis of iRFP670 fluorescence intensities for individual cells (20 cells from three independent experiments) in (A).


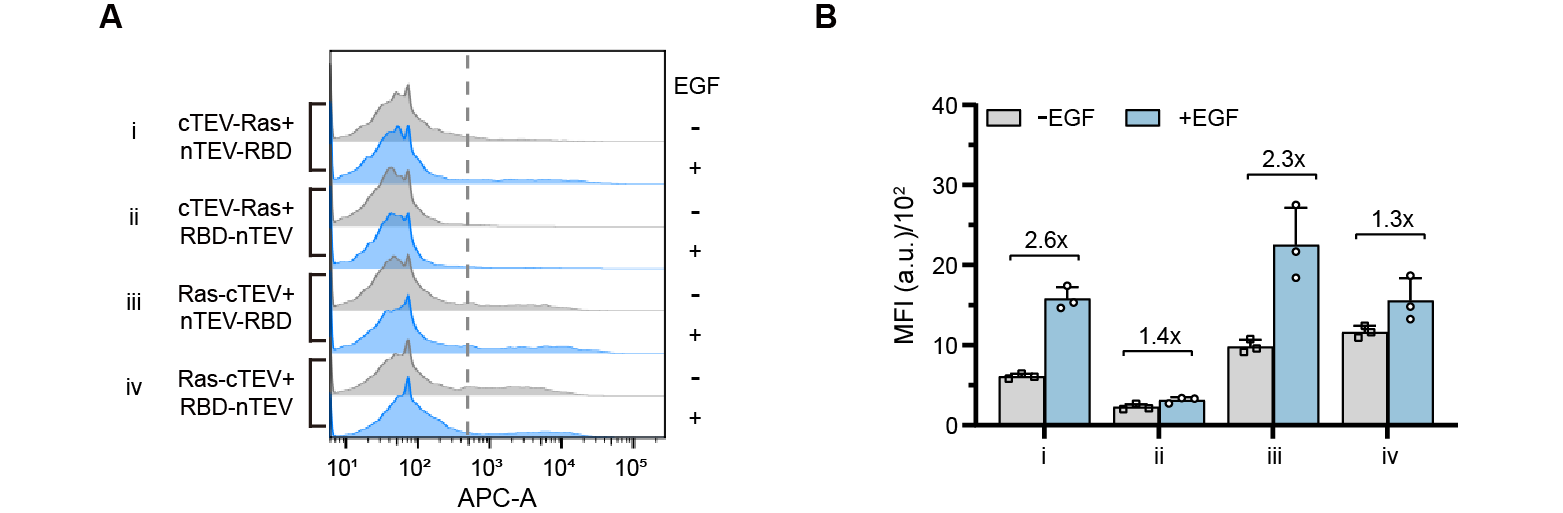


**Figure S35. Screening different fusion combinations of Ras/RBD domains and split TEVp fragments.** (A) Flow cytometry profiles for HeLa cells expressing RAS-responsive RESIT system with different fusion combinations with or without EGF (50 ng/ml) induction. (B) MFI of iRFP670 for cells in (A). Cells expressing the RESIT system with the combination of nTEVp−RBD and cTEVp−RAS exhibited the best fold change. Error bars represented s.d. of three independent experiments.


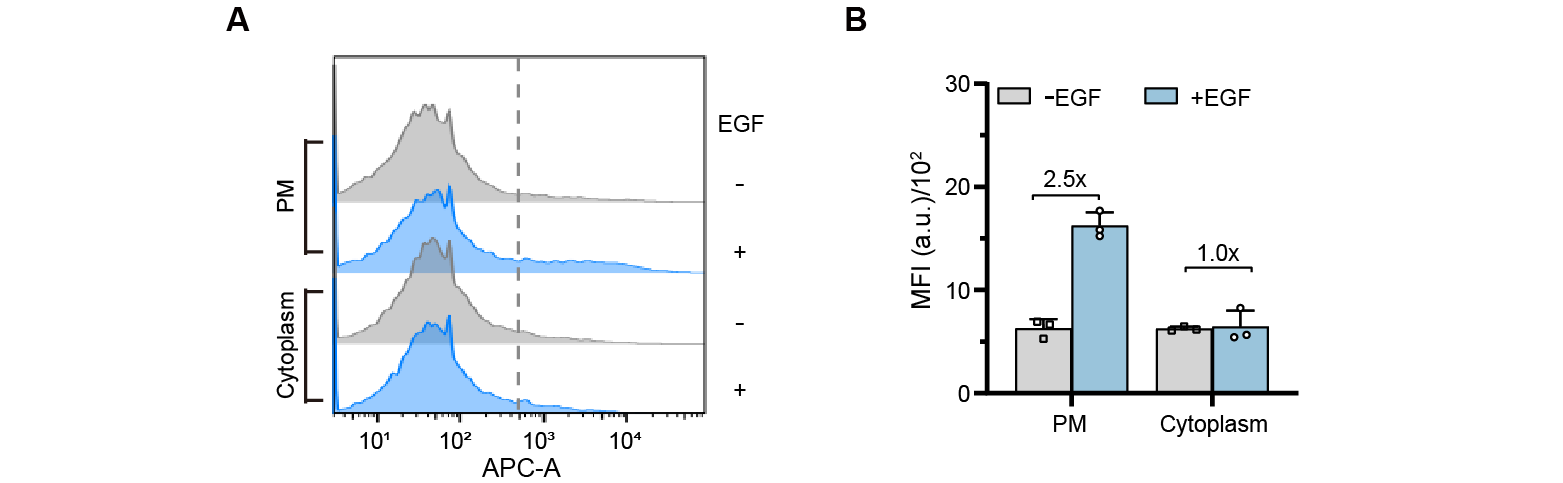


**Figure S36.** **Comparison of membrane-tethered RESIT and cytosol-localized RESIT in sensing Ras activity modules.** (A) Flow cytometry profiles for HeLa cells expressing the membrane-tethered RAS-responsive RESIT system (PM) or the cytosol-localized counterpart (Cytoplasm) with or without EGF (50 ng/ml) induction. (B) MFI of iRFP670 for cells in (A). Error bars represented s.d. of three independent experiments.


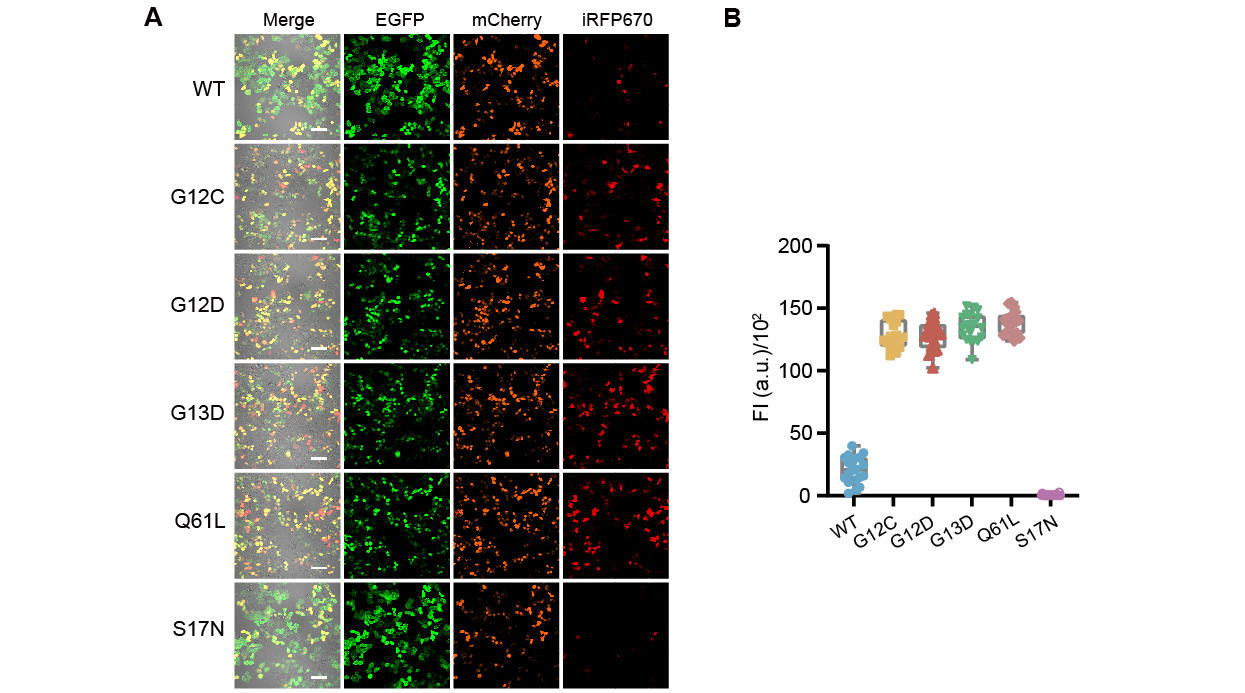


**Figure S37.** **Confocal images for cells expressing RESIT system in sensing the activities of Ras mutants.** (A) Representative confocal images for HeLa cells expressing RAS-responsive RESIT system with wide type (WT) or Ras mutants (G12C, G12D, G13D, Q61L and S17N) in the sensing modules. iRFP670 fluorescence indicated the reporters. EGFP fluorescence indicated the expression of tGal4(65) −nTEVp−RBD. mCherry indicated the expression of tGal4(65)−cTEVp−Ras. Scale bar, 100 µm. (B) Quantitative analysis of iRFP670 fluorescence intensities for individual cells (20 cells from three independent experiments) in (A).


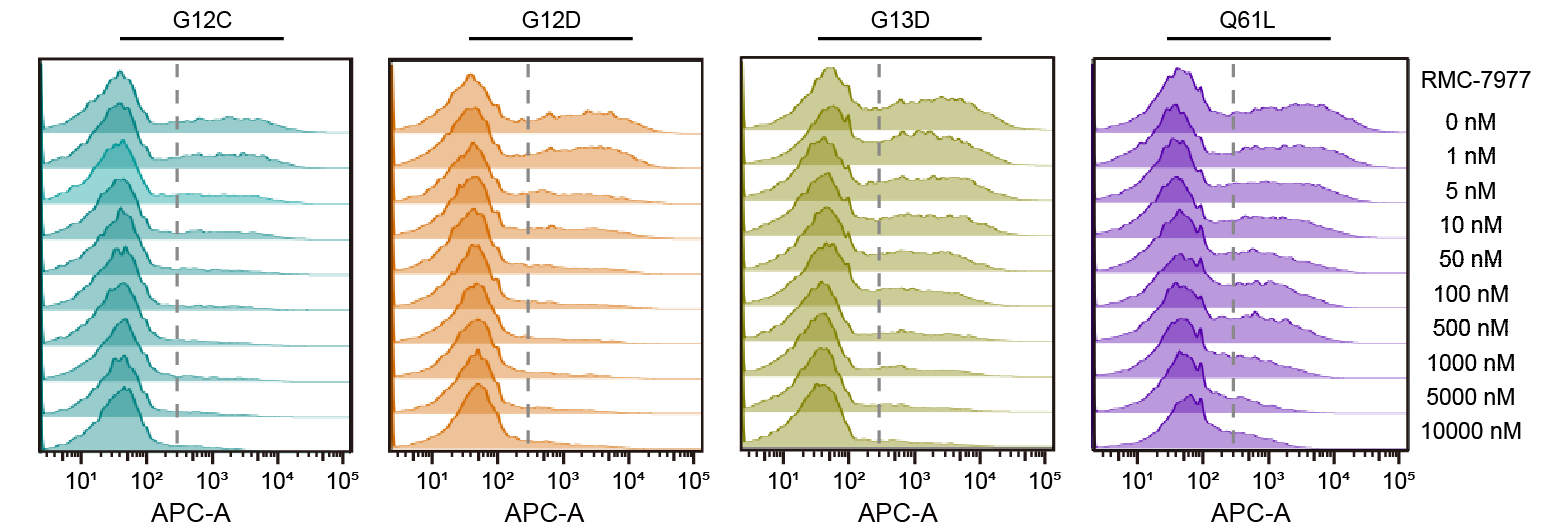


**Figure S38.** **Dose-dependent impact of RMC-7977 on the activities of different Ras mutants.** Dose-dependent effect of RMC-7977 on Ras mutants (G12C, G12D, G13D, and Q61L) in HeLa cells. Flow cytometry profile for cells expressing the RESIT systems with different Ras mutants (G12C, G12D, G13D, and Q61L) treated with different concentrations of RMC-7977.


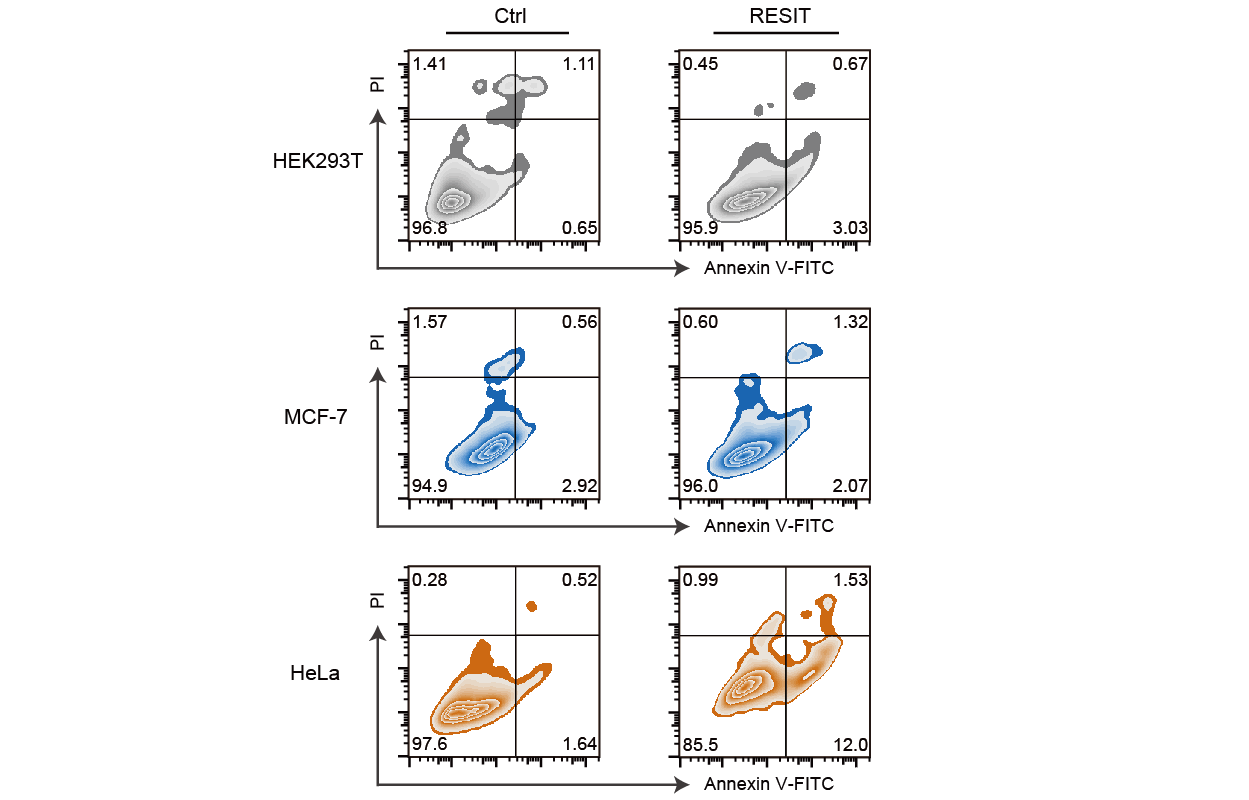

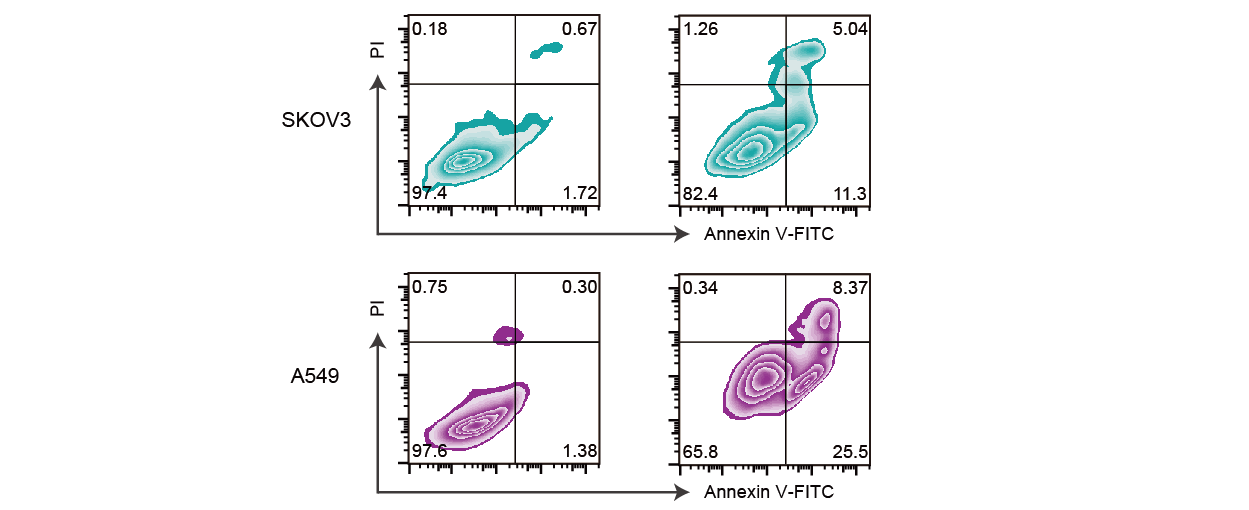


**Figure S39.** **RESIT system for apoptosis induction in cells with different RTK activities.** Flow cytometry profiles for different cells (HEK293T, MCF-7, HeLa, SKOV3 and A549) expressing the RTK-responsive RESIT system using hBax as the effector costained with annexin V-FITC and PI. Ctrl, cells without transfection. A549 and SKOV3 cells with the highest RTK activity exhibited substantial apoptosis, HeLa cells with moderate RTK activity displayed remarkable apoptosis, and MCF-7 and HEK293T cells with the lowest RTK activity exhibited little apoptosis.


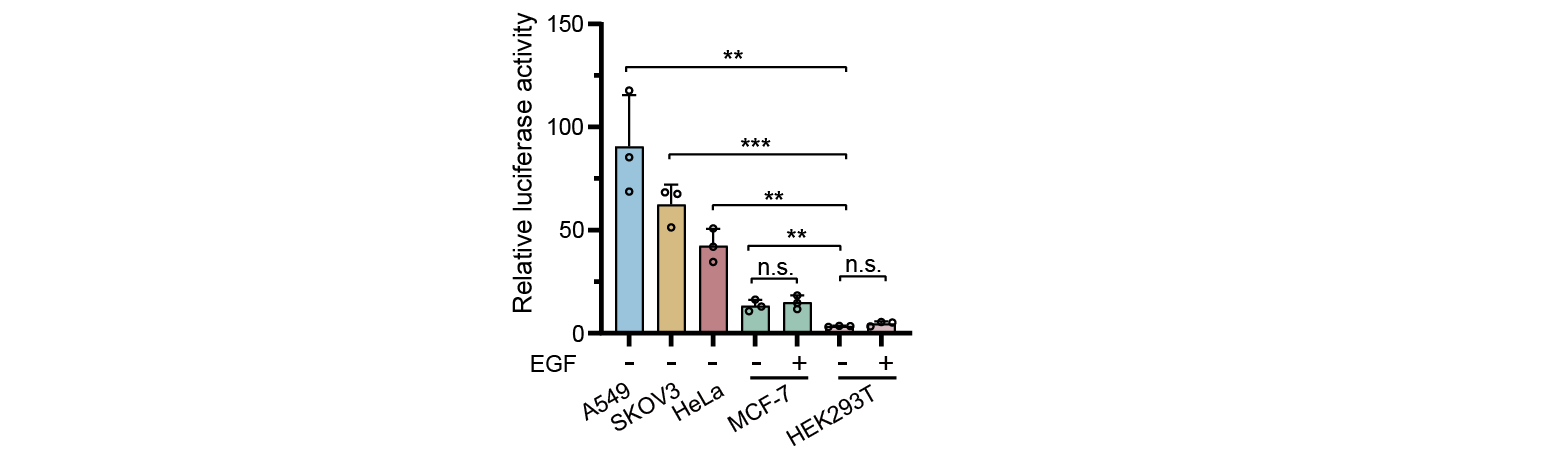


**Figure S40. Secretion of LYTAC in cells with different RTK activities.** Secretion of LYTAC for different cell lines (A549, SKOV3, HeLa, MCF-7 and HEK293T) expressing the RTK-responsive RESIT system using Gluc-LYTAC as the effector with or without EGF induction. The levels of secreted Gluc were measured in the supernatants of cultured cells. Data are represented as mean ± s.d. of three independent measurements. A549 and SKOV3 cells with the highest RTK activity exhibited the most secretion of LYTAC, HeLa cells with moderate RTK activity displayed remarkable secretion of LYTAC, and MCF-7 and HEK293T cells with lowest RTK activity showed negligible secretion of LYTAC. Statistical analysis was performed using a two-tailed t-test (**p = 0.0038 for A549 cells, ***p = 0.0004 for SKOV3 cells, **p = 0.0011 for Hela cells and **p = 0.0038 for MCF-7 cells, respectively, as compared to HEK293T cells. p = 0.5728 for MCF-7 cells with or without EGF induction, and p = 0.1344 for HEK293T cells with or without EGF induction).


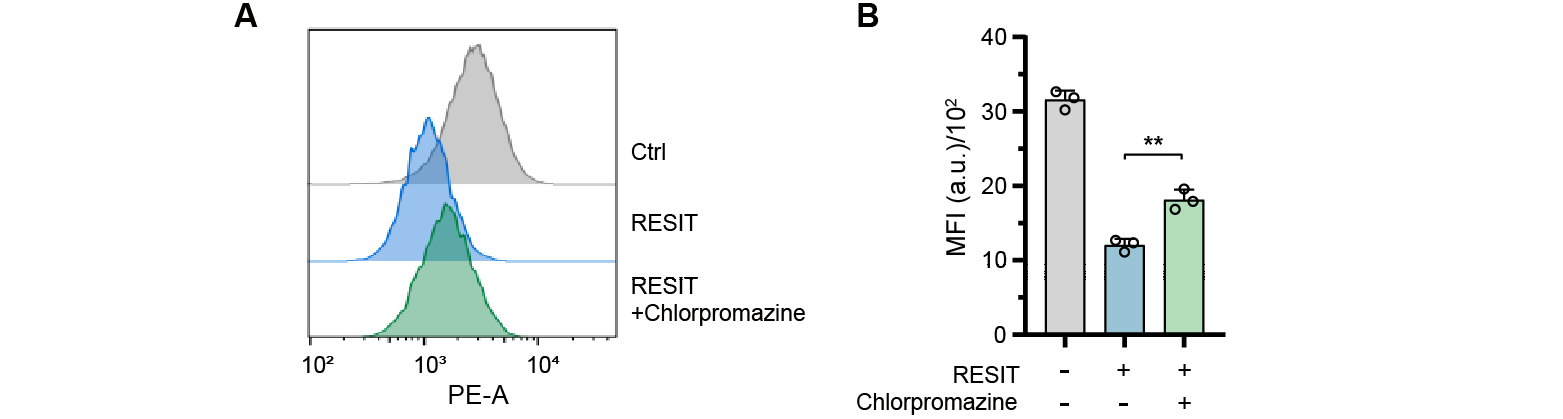


**Figure S41. Effect of endocytosis inhibitor on EGFR degradation for cells expressing RTK-responsive RESIT system.** (A) Flow cytometry profiles for EGFR levels on cell surface of SKOV3 cells expressing the RTK-responsive RESIT system using LYTAC as the effector treated with or without the endocytosis inhibitor chlorpromazine (1 μM). The cells under different treatments were stained by PE-labeled anti-EGFR antibody. (B) MFI of mCherry for cells in (A). The surface EGFR level was restored for cells treated with chlorpromazine, indicating endocytosis-mediated degradation. Data are represented as mean ± s.d. of three independent measurements. Statistical analysis was performed using a two-tailed t-test (**p = 0.0028).


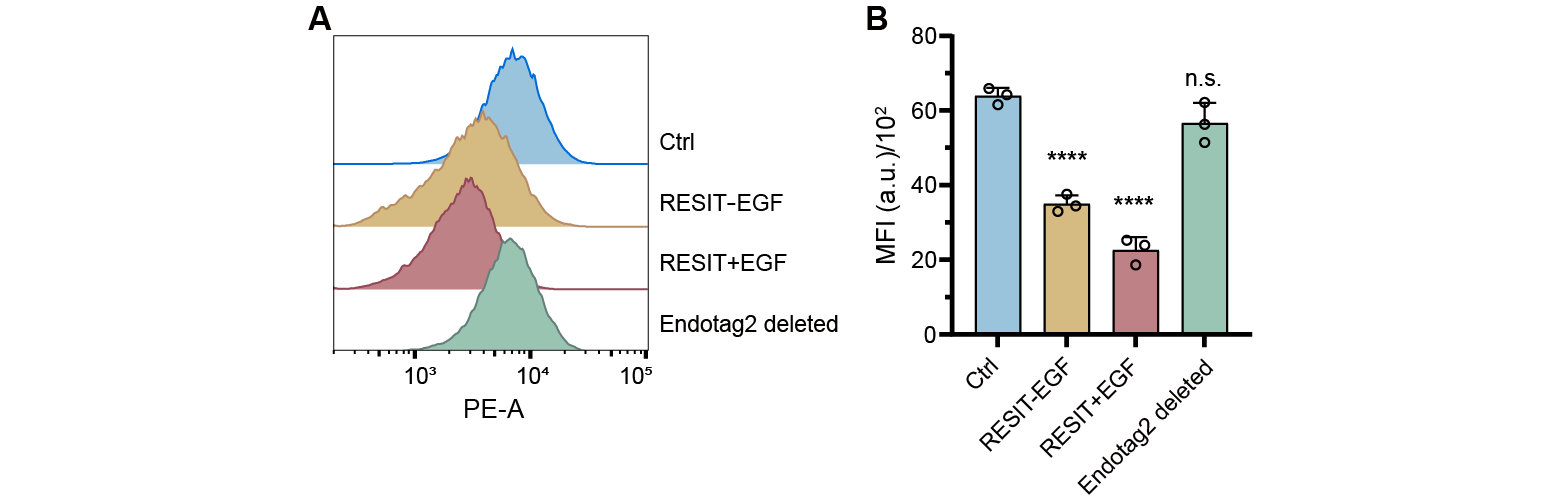


**Figure S42. Specificity of RTK-responsive RESIT system using LYTAC as the effector protein.** (A) Flow cytometry profile for EGFR levels on cell surface of A549 cells expressing RTK-responsive RESIT system using LYTAC as the effector protein with Endotag2 deleted under EGF induction. Ctrl, cells without transfection. The cells under different treatment were stained by PE-labeled anti-EGFR antibody. (B) MFI of mCherry signals for cells under different treatments in (A). Negligible EGFR degradation was detected for RESIT system using LYTAC as the effector protein with Endotag2 deleted, verifying the specific degradation of EGFR via LYTAC. Data are represented as mean ± s.d. of three independent measurements. The significance of differences for cells under different treatments versus Ctrl was determined using single-factor ANOVA (Left to right: ****p < 0.0001, ****p < 0.0001, p = 0.0921).


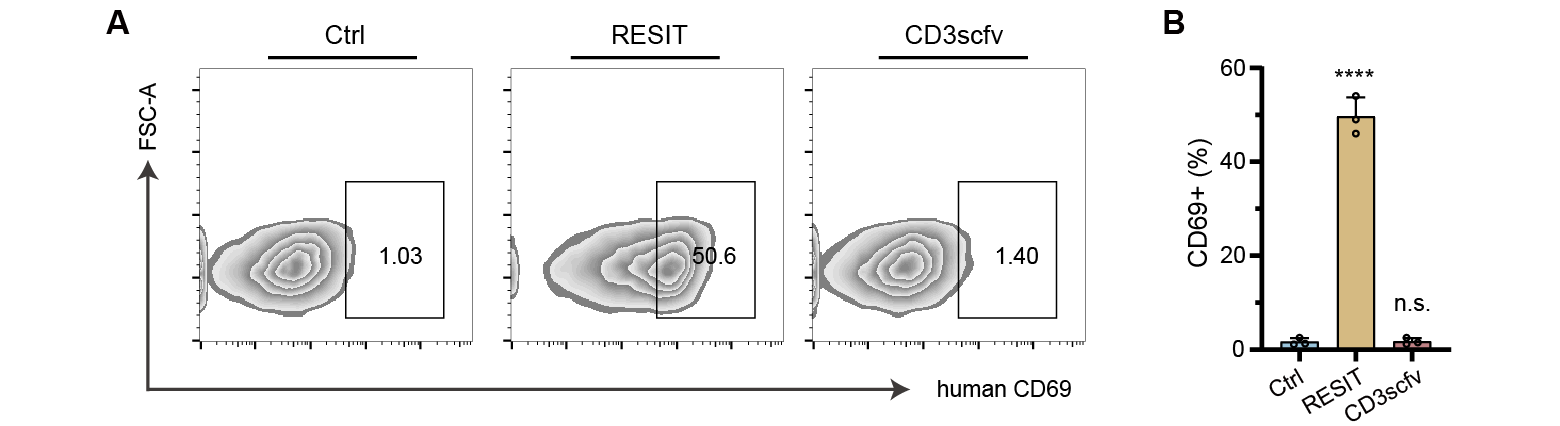


**Figure S43. Specificity of RTK-responsive RESIT system using BiTE as the effector protein.** (A) Flow cytometry profiles for Jurkat T cells cocultured with HeLa cells expressing RTK-responsive RESIT system using BiTE (RESIT) or scfv for CD3 only (CD3scfv) as the effector co-stained with APC-labeled anti-CD69 antibody. (B) Percentages of CD69^+^ T cell for cells in (A). Negligible activation of Jurkat T cells was observed after co-culturing with HeLa cells expressing RESIT system using CD3scfv as the effector, verifying the specific activation of RESIT by BiTE. The significance of differences for cells under different treatments versus blank cells without any treatment was tested single-factor ANOVA (Left to right: ****p < 0.0001, p = 0.9986).


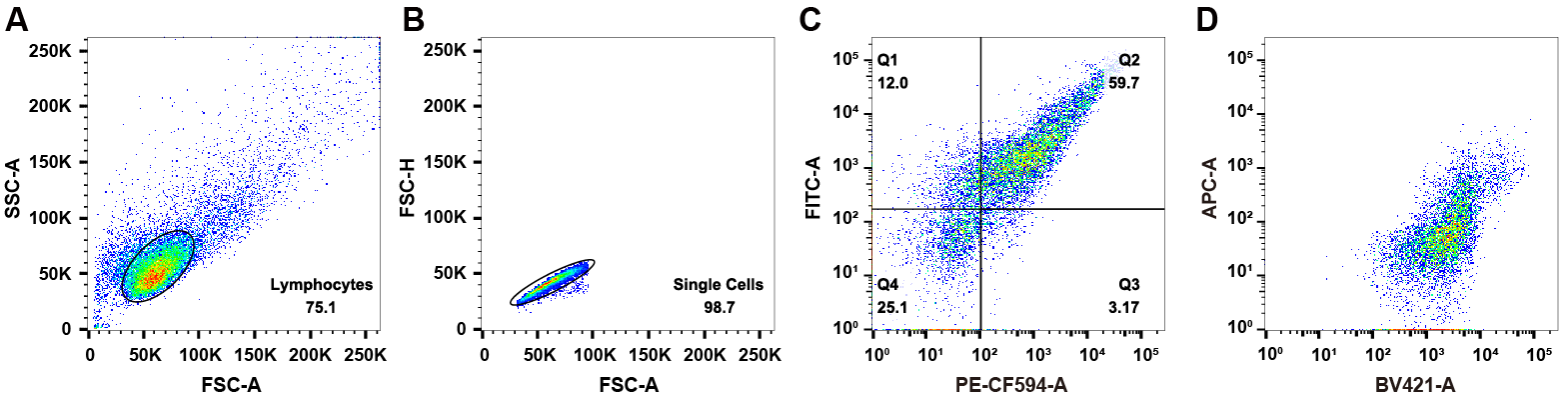


**Figure S44. Representative gating settings for flow cytometry analysis.** (A) Cell events are gated based on side versus forward scatter (SSC-A versus FSC-A) to identify cell population. (B) Single cells are gated based on forward scatter height versus forward scatter area (FSC-H versus FSC-A). (C) mCherry/EGFP double-positive cells are gated based on PE-CF594-A (mCherry) and FITC-A (EGFP) fluorescence intensities. (D) The internal control BFP is used to normalize expression differences among different cell lines.

# III. Supplementary Tables

**Table S1.** Amino acid sequences for main RESIT domains.

| **Protein** | **Amino acid sequences** |
| --- | --- |
| N-myr | MGSSKSKPKDPSQR |
| PH domain | MDSGRDFLTLHGLQDDEDLQALLKGSQLLKVKSSSWRRERFYKLQEDCKTIWQESRKVMRTPESQLFSIEDIQEVRMGHRTEGLEKFARDVPEDRCFSIVFKDQRNTLDLIAPSPADAQHWVLGLHKIIHHSGSMDQRQKLQHWIHSCLRKADKNKDNKMSFKELQNFLKELNIQ |
| FKBP | MGVQVETISPGDGRTFPKRGQTCVVHYTGMLEDGKKFDSSRDRNKPFKFMLGKQEVIRGWEEGVAQMSVGQRAKLTISPDYAYGATGHPGIIPPHATLVFDVELLKLE |
| FRB^T2098L^ | ILWHEMWHEGLEEASRLYFGERNVKGMFEVLEPLHAMMERGPQTLKETSFNQAYGRDLMEAQEWCRKYMKSGNVKDLLQAWDLYYHVFRRISK |
| Gal4 | MKLLSSIEQACDICRLKKLKCSKEKPKCAKCLKNNWECRYSPKTKRSPLTRAHLTEVESRLERLEQLFLLIFPREDLDMILKMDSLQDIKALLTGLFVQDNVNKDAVTDRLASVETDMPLTLRQHRISATSSSEESSNKGQRQLTVS |
| tGal4(65) | MKLLSSIEQACDICRLKKLKCSKEKPKCAKCLKNNWECRYSPKTKRSPLTRAHLTEVESRLERLE |
| GCN4 | MKDPAALKRARNTEAARRSRARKLQRMKQLEDKVEELLSKNYHLENEVARLKKLVGER |
| tGCN4(274) | MKDPAALKRARNTEAARRSRARKLQRMKQLEDKVEELLSKNYHLENEVARL |
| TetR | MSRLDKSKVINSALELLNEVGIEGLTTRKLAQKLGVEQPTLYWHVKNKRALLDALAIEMLDRHHTHFCPLEGESWQDFLRNNAKSFRCALLSHRDGAKVHLGTRPTEKQYETLENQLAFLCQQGFSLENALYALSAVGHFTLGCVLEDQEHQVAKEERETPTTDSMPPLLRQAIELFDHQGAEPAFLFGLELIICGLEKQLKCESGS |
| tTetR(196) | MSRLDKSKVINSALELLNEVGIEGLTTRKLAQKLGVEQPTLYWHVKNKRALLDALAIEMLDRHHTHFCPLEGESWQDFLRNNAKSFRCALLSHRDGAKVHLGTRPTEKQYETLENQLAFLCQQGFSLENALYALSAVGHFTLGCVLEDQEHQVAKEERETPTTDSMPPLLRQAIELFDHQGAEPAFLFGLELIICG |
| nTEVp | GESLFKGPRDYNPISSTICHLTNESDGHTTSLYGIGFGPFIITNKHLFRRNNGTLLVQSLHGVFKVKNTTTLQQHLIDGRDMIIIRMPKDFPPFPQKLKFREPQREERICLVTTNFQ |
| cTEVp | MSSMVSDTSCTFPSSDGIFWKHWIQTKDGQCGSPLVSTRDGFIVGIHSASNFTNTNNYFTSVPKNFMELLTNQEAQQWVSGWRLNADSVLWGGHKVFM |
| nPPVp | MSKSLFRGLRDYNPIASSICQLNNSSGARQSEMFGLGFGGLIVTNQHLFKRNDGELTIRSHHGEFVVKDTKTLKLLPCKGRDIVIIRLPKDFPPFPKRLQFRTPTTEDRVCLIGSNFQT |
| cPPVp | KSISSTMSETSATYPVDNSHFWKHWISTKDGHCGLPIVSTRDGSILGLHSLANSTNTQNFYAAFPDNFETTYLSNQDNDNWIKQWRYNPDEVCWGSLQLKRDIPQSPFTICKLLTDLDGEFVYTQ |
| nSbMVp | SKSVYKGLRDYSGISTLICQLTNSSDGHKETMFGVGYGSFIITNGHLFRRNNGMLTVKTWHGEFVIHNTTQLKIHFIQGRDVILIRMPKDFPPFGKRNLFRQPKREERVCMVGTNFQE |
| cSbMVp | KSLRATVSESSMILPEGKGSFWIHWITTQDGFCGLPLVSVNDGHIVGIHGLTSNDSEKNFFVPLTDGFEKEYLENADNLSWDKHWFWEPSKIAWGSLNLVEEQPKEEFKISKLVSDLFGNTVTVQ |
| PTB | MGKPLHPNDKVMGPGVSYLVRYMGCVEVLQSMRALDFNTRTQVTREAISLVCEAVPGAKGATRRRKPCSRPLSSILGRSNLKFAGMPITLTVSTSSLNLMAADCKQIIANHHMQSISFASGGDPDTAEYVAYVAKDPVNQRACHILECPEGLAQDVISTIGQAFELRFKQYLRDIEQVPQQPTLK |
| VAV1 SH2 | GPPQDLSVHLWYAGPMERAGAESILANRSDGTFLVRQRVKDAAEFAISIKYNVEVKHIKIMTAEGLYRITEKKAFRGLTELVEFYQQNSLKDCFKSLDTTLQFPFKEPEKRGSR |
| 3x substrate | RPAGSVQNPVYHNQPLNPAPSRDPHYQDPHSTAVGNPEYLNTVQPTCVNSTFDSPAHWAQKGSHQISLDNPDYQQDFFPKEAK |
| KRas | MTEYKLVVVGAGGVGKSALTIQLIQNHFVDEYDPTIEDSYRKQVVIDGETCLLDILDTAGQEEYSAMRDQYMRTGEGFLCVFAINNTKSFEDIHHYREQIKRVKDSEDVPMVLVGNKCDLPSRTVDTKQAQDLARSYGIPFIETSAKTRQGVDDAFYTLVREIRKHKEKMSKDG |
| RAF-RBD | SSNTIRVFLPNKQRTVVNVRNGMSLHDCLMKALKVRGLQPECCAVFRLLHEHKGKKARLDWNTDAASLIGEELQVDFL |
| CaM | PDQLTEEQIAEFKEAFSLFDKDGDGTITTKELGTVMRSLGQNPTEAELQDMINEVDADGDGTIDFPEFLTMMARKMKYRDTEEEIREAFGVFDKDGNGYISAAELRHVMTNLGEKLTDEEVDEMIREADIDGDGQVNYEEFVQMMTAK |
| skMLCK | KRRWKKNFIAVSAANRFKKISSSGAL |
| CaMKII | FNARRKLAGAILFTMLATRNFS |
| smMLCK | RRKWNKTGHAVRAIGRLSS |
| GLuc | MGVKVLFALICIAVAEAKPTENNEDFNIVAVASNFATTDLDADRGKLPGKKLPLEVLKEMEANARKAGCTRGCLICLSHIKCTPKMKKFIPGRCHTYEGDKESAQGGIGEAIVDIPEIPGFKDLEPMEQFIAQVDLCVDCTTGCLKGLANVQCSDLLKKWLPQRCATFASKIQGQVDKIKGAGGD |
| Bax | MDGSGEQPRGGGPTSSEQIMKTGALLLQGFIQDRAGRMGGEAPELALDPVPQDASTKKLSECLKRIGDELDSNMELQRMIAAVDTDSPREVFFRVAADMFSDGNFNWGRVVALFYFASKLVLKALCTKVPELIRTIMGWTLDFLRERLLGWIQDQGGWDGLLSYFGTPTWQTVTIFVAGVLTASLTIWKKMG |
| aEGFR | QVKLEESGGGSVQTGGSLRLTCAASGRTSRSYGMGWFRQAPGKEREFVSGISWRGDSTGYADSVKGRFTISRDNAKNTVDLQMNSLKPEDTAIYYCAAAAGSAWYGTLYEYDYWGQGTQVTVSS |
| HER2 nanobody | MHVDNKFNKEMRNAYWEIALLPNLNNQQKRAFIRSLYDDPSQSANLLAEAKKLNDAQAPK |
| Endotag2 | MVEASLWLLIWDAGELVERGNPEEARKVLEEAREIAERNNREEFLKELEVLLEKLEGGSTERRVIQVLEQILEDEDPEVVEKMLEILLEILEEAGDPARKLVEEILRVVRKNLEEARELVRRLS |
| CD3 scfv | DIQMTQTTSSLSASLGDRVTISCRASQDIRNYLNWYQQKPDGTVKLLIYYTSRLHSGVPSKFSGSGSGTDYSLTISNLEQEDIATYFCQQGNTLPWTFAGGTKLEIKGGGSGGGSGGGSGGGSEVQLQQSGPELVKPGASMKISCKASGYSFTGYTMNWVKQSHGKNLEWMGLINPYKGVSTYNQKFKDKATLTVDKSSSTAYMELLSLTSEDSAVYYCARSGYYGDSDWYFDVWGAGTTVTVSS |

# IV. References

1. X. Wang, X. Chen, Y. Yang, “Spatiotemporal control of gene expression by a light-switchable transgene system,” *Nature Methods* 9 (2012): 266, <https://doi.org/10.1038/nmeth.1892>.

2. M. Cui, S. Lee, S. H. Ban, J. R. Ryu, M. Shen, S. H. Yang, J. Y. Kim, S. K. Choi, J. Han, Y. Kim, K. Han, D. Lee, W. Sun, H. B. Kwon, D. Lee, “A single-component, light-assisted uncaging switch for endoproteolytic release,” *Nature Chemical Biology* 20 (2024): 353, <https://doi.org/10.1038/s41589-023-01480-6>.

3. H. D. Wu, M. Kikuchi, O. Dagliyan, A. K. Aragaki, H. Nakamura, N. V. Dokholyan, T. Umehara, T. Inoue, “Rational design and implementation of a chemically inducible heterotrimerization system,” *Nature Methods* 17 (2020): 928, <https://doi.org/10.1038/s41592-020-0913-x>.

4. S. Kedracka-Krok, Z. Wasylewski, “Kinetics and equilibrium studies of Tet repressor-operator interaction,” *Journal of Protein Chemistry* 18 (1999): 117, <https://doi.org/10.1023/a:1020611919599>.

5. P. Orth, D. Schnappinger, W. Hillen, W. Saenger, W. Hinrichs, “Structural basis of gene regulation by the tetracycline inducible Tet repressor-operator system,” *Nature Structural & Molecular Biology* 7 (2000): 215, <https://doi.org/10.1038/73324>.

6. T. E. Ellenberger, C. J. Brandl, K. Struhl, S. C. Harrison, “The GCN4 basic region leucine zipper binds DNA as a dimer of uninterrupted alpha helices: crystal structure of the protein-DNA complex,” *Cell* 71 (1992): 1223, <https://doi.org/10.1016/s0092-8674(05)80070-4>.
